# Rapid Global Fitting of Large Fluorescence Lifetime Imaging Microscopy Datasets

**DOI:** 10.1371/journal.pone.0070687

**Published:** 2013-08-05

**Authors:** Sean C. Warren, Anca Margineanu, Dominic Alibhai, Douglas J. Kelly, Clifford Talbot, Yuriy Alexandrov, Ian Munro, Matilda Katan, Chris Dunsby, Paul M. W. French

**Affiliations:** 1 Institute for Chemical Biology, Department of Chemistry Imperial College London, London, United Kingdom; 2 Photonics Group, Department of Physics, Imperial College London, London, United Kingdom; 3 Department of Structural and Molecular Biology, University College London, London, United Kingdom; 4 Department of Histopathology, Imperial College London, London, United Kingdom; University of California, Berkeley, United States of America

## Abstract

Fluorescence lifetime imaging (FLIM) is widely applied to obtain quantitative information from fluorescence signals, particularly using Förster Resonant Energy Transfer (FRET) measurements to map, for example, protein-protein interactions. Extracting FRET efficiencies or population fractions typically entails fitting data to complex fluorescence decay models but such experiments are frequently photon constrained, particularly for live cell or in vivo imaging, and this leads to unacceptable errors when analysing data on a pixel-wise basis. Lifetimes and population fractions may, however, be more robustly extracted using global analysis to simultaneously fit the fluorescence decay data of all pixels in an image or dataset to a multi-exponential model under the assumption that the lifetime components are invariant across the image (dataset). This approach is often considered to be prohibitively slow and/or computationally expensive but we present here a computationally efficient global analysis algorithm for the analysis of time-correlated single photon counting (TCSPC) or time-gated FLIM data based on variable projection. It makes efficient use of both computer processor and memory resources, requiring less than a minute to analyse time series and multiwell plate datasets with hundreds of FLIM images on standard personal computers. This lifetime analysis takes account of repetitive excitation, including fluorescence photons excited by earlier pulses contributing to the fit, and is able to accommodate time-varying backgrounds and instrument response functions. We demonstrate that this global approach allows us to readily fit time-resolved fluorescence data to complex models including a four-exponential model of a FRET system, for which the FRET efficiencies of the two species of a bi-exponential donor are linked, and polarisation-resolved lifetime data, where a fluorescence intensity and bi-exponential anisotropy decay model is applied to the analysis of live cell homo-FRET data. A software package implementing this algorithm, FLIMfit, is available under an open source licence through the Open Microscopy Environment.

## Introduction

### Background

Imaging of Förster Resonant Energy Transfer (FRET) between proteins conjugated with suitable fluorophores has become a powerful tool for biologists to study cellular processes with spatial and temporal resolution [1], [2]. The efficiency of FRET varies as the inverse sixth power of distance between fluorophores, typically reaching 50% at 2–8 nm, and this strong distance dependence allows the detection and/or quantification of protein-protein interactions or changes in protein conformation. There are many reported approaches to detect and quantify FRET, of which the most widely used imaging modalities are probably spectral ratiometric imaging of donor and acceptor fluorophore emission, fluorescent lifetime imaging (FLIM) of the donor emission and fluorescence anisotropy imaging of the acceptor emission. FLIM, which maps the decrease in donor fluorescence lifetime due to FRET, has a number of advantages, particularly for imaging in living cells and organisms. The changes in donor lifetime upon FRET are generally independent of the fluorophore concentration, the excitation and detection efficiencies and scattering and sample absorption. Fluorescence lifetime measurements are also relatively robust in the presence of spectral crosstalk and are relatively insensitive to donor–acceptor stoichiometry, since it is only the donor fluorescence that is measured. They therefore do not require parallel spectral calibration measurements and are independent of the optical system (instrument and sample), which is particularly important for *in vivo* applications. Fluorescence lifetime can also be used to distinguish between different fluorophores and to read out variations in the local fluorophore environment [3].

FLIM may be implemented in the time domain using periodic pulsed excitation or in the frequency domain using sinusoidally modulated or pulsed excitation [4]. This paper is concerned with time domain analysis, for which fluorescence decay profiles are typically measured using time-correlated single photon counting (TCSPC) in laser scanning microscopes or time-gated detection in wide-field microscopes. For TCSPC, histograms are constructed from single photon detection events across equally spaced time bins that sample the whole decay profile, while for time-gated imaging the decay profiles can be sampled at periodic or arbitrary delays after excitation with equal or varying widths of time gate or image integration time [5]. Fluorescence lifetime parameters may be analytically determined from time-gated data using rapid lifetime determination with either a mono- or bi-exponential model, however higher precision may be obtained at lower signal levels using nonlinear fitting [6]. Analysis of TCSPC data and optimal precision with time-gated data requires the use of nonlinear fitting [6].

For frequency domain FLIM, the change in phase and modulation depth of the fluorescence signal with respect to the excitation signal is measured at one or more modulation frequencies. Again, lifetimes of mono-exponential decay profiles may be calculated using simple analytical approaches while complex decay profiles can be analysed using nonlinear fitting algorithms.

Alternatively, FLIM data may be analysed graphically, e.g. using the increasingly popular ‘phasor’ approach [7], [8] that provides an immediate indication of the complexity of fluorescence decay profiles and can yield lifetime values for mono- or bi-exponential decays by linear fitting to distributions of points on the phasor plots – which can be done by inspection for ‘well behaved’ data. This approach is directly applicable to data acquired in the frequency domain, to data acquired using TCSPC and also to periodically sampled time-gated data [9] but has yet to be extended to the more photon efficient time-gating strategies employing non-periodically sampled data or overlapping time gates [6], [10].

A major challenge for the quantification of FLIM (and FLIM FRET) is the need to acquire sufficient photons to achieve the precision in lifetime determination required to distinguish and quantify different FRET states. In general FLIM-FRET decay profiles are not well described by a mono-exponential model; in the simplest example of FRET between two fluorophores with mono-exponential decay profiles conjugated to interacting proteins, there will be a mixture of interacting and non-interacting populations leading to a bi-exponential decay profile. The decay profile will be considerably more complex if the donor itself exhibits a multi-exponential decay, as is the case for, e.g. ECFP [11]. It has been calculated that tens of thousands of photons are required to accurately fit a bi-exponential decay [4], [12]; this is at least an order of magnitude greater the number available for each pixel from typical live cell or *in vivo* FLIM-FRET experiments. The rate at which fluorescence photons can be practically detected is usually limited by photobleaching and/or phototoxicity and so the minimum detected photon number requirements for FLIM can constrain the (time lapse) resolution of time-course experiments and can make image acquisition times unacceptably long for high content/throughput assays.

To address the issue of FLIM measurements with low numbers of detected photons per pixel, it is possible to ameliorate the fitting challenge by assuming that there are only a limited number of fluorescence lifetime components across an entire image or dataset. This could result from, for example, a FRET biosensor such as the Cameleon calcium sensor [13] that can be assumed to be in an ‘open’ (low FRET) or ‘closed’ (high FRET) conformation, depending on whether it is bound to a calcium ion. Similarly, a donor-labelled protein can be considered to be bound or not bound to its acceptor-labelled ligand. Global analysis may then be used to simultaneously fit an entire dataset assuming that only the fractional contributions of a limited number of unknown but spatially invariant lifetime components vary from pixel to pixel.

### State of the Art of Global Fluorescence Decay Analysis

Beecham demonstrated that the simultaneous analysis of multiple datasets could be used to reduce the uncertainty in parameter estimation as the combined data more strongly constrains the error surface [14]. Verveer et al. [15] first applied global analysis to FLIM data in the frequency-domain, using two approaches. In the first approach, termed ‘lifetime invariant fitting’ or ‘*global binning*’, the decay profile data are integrated across the image to give a single decay profile that can be fitted to a complex decay model. The lifetime components produced by this fit are then fixed across the image and the contributions of the two components are then determined by fitting on a pixelwise basis. In the second approach, which we describe as ‘*global fitting*’, the lifetimes and contributions were fitted simultaneously using a truncated Newton algorithm. The global fitting approach is able to exploit the spatial variation of contributions across the image that is lost in the global binning approach, providing stronger constraints on the lifetime estimates, albeit at the cost of significantly more computation. Pelet et al. [16] compared these two approaches on simulated and experimental TCSPC data and found that global binning failed to converge to the correct lifetime values for experimental data more often that the global fitting approach. They additionally demonstrated an intensity image segmentation based approach to determining initial guesses for the unknown parameters that could improve the time required for global analysis of a 64×64 pixel TCSPC image from 3 hours to 12 minutes using MATLAB large scale optimisation routines.

Barber et al. [17] subsequently developed a global fitting software package based on nonlinear least squares fitting for time-domain FLIM data using a multi-exponential model with a constant background that makes use of the sparse nature of the Jacobian matrix to reduce the computational complexity. Using a nonlinear least squares Levenberg-Marquart fitting algorithm, they demonstrated that global analysis may be performed across several datasets [18]. To further address the computational burden of global fitting, Visser et al. [19] applied the TIMP separable nonlinear least squares fitting package, written in the programming language R, to global analysis of FLIM data. This approach uses the fact that a multi-exponential decay may be expressed as the linear sum of a number of nonlinear components and reduces the memory requirements dramatically. It also reduces the number of iterations required for convergence by reducing the number of nonlinear parameters in the fit. TIMP did not extend the variable projection approach to use analytical derivatives and so used numerically computed derivatives.

Although these global fitting approaches are typically faster than pixel-wise nonlinear analysis of image data, concerns remain about the scalability of current nonlinear fitting methods to very large datasets and more complex decay models. In recent years improvements in the performance potential of new generations of processors has come through the availability of multiple cores able to process operations in parallel while the increase in performance of individual cores has slowed. Previous global analysis implementations have been strictly single threaded, limiting their ability to capitalise on hardware improvements and run efficiently on modern CPUs. Recent developments in high-content and high-throughput FLIM microscopy platforms have enabled the capture of datasets with hundreds or thousands of time-resolved images in a single acquisition session [20]–[23]. Such datasets can easily exceed several gigabytes in size and it is highly desirable that the processing and analysis of the data is practical on conventional workstations and does not become a bottleneck for such assays. We believe that the software presented here addresses this increasingly important need.

### Our Approach

In this paper we employ a separable nonlinear least square fitting algorithm based on the variable projection technique of Golub and Pereyra [24], extending the approach employed by TIMP [19] to use analytical derivatives, considered important for ensuring convergence [25]. Crucially, in order to allow the scaling of this approach to the global analysis of large datasets, the code is carefully optimised from data-loading to display and processing of results to minimise memory usage and implemented using multithreaded parallel algorithms to enable effective scaling on multicore processors. The resulting software tool is called *FLIMfit* and we demonstrate that it is able to routinely analyse multi-well plate FLIM datasets on conventional PC workstations in a reasonable time. For example a 394 image multiwell time-gated FLIM dataset with five time gates, with each image containing 672×512 pixels, required 32 seconds and 2 GB of memory to analyse globally.

#### Accounting for instrumental and experimental effects

Our software is able to account for instrumental effects and experimental limitations such as background fluorescence from plastic multi-well plates and contributions from previous pulses due to the use of high repetition rate lasers. In addition, it can analyse either time-binned TCSPC data or time-gated FLIM data (with arbitrary temporal sampling profiles) and can account for the temporal instrument response function (IRF) using either a direct measurement of the IRF or a measurement of a reference fluorophore with a known mono-exponential decay profile using the delta-function convolution method [26], [27]. Contributions from a constant background of stray light, scattered excitation light and a time-varying noise background can be taken into account if appropriate background measurements are provided. The effect of incomplete fluorescence decay profiles from previous excitation pulses are included in the model, as previously demonstrated by Rowley et al. [28].

#### Handling FRET donors with complex fluorescence decay profiles

For the common case of a FRET system with a bi-exponential donor, for example ECFP [29], [30], paired with a mono-exponential acceptor such as EYFP [31], the resulting complex decay can be approximated by four exponential decay components: two associated with the different donor conformations alone and two associated with these conformations undergoing FRET. We demonstrate a constrained model to fit such data, where the FRET efficiencies of the two donor conformational states are linked via their relative quantum yields and the relative contributions of the two states are assumed to be in dynamic equilibrium. We apply the model to fit multiwell plate time-gated FLIM FRET (ECFP/EYFP) data from an assay of the aggregation of HIV Gag protein into virus like particles in HeLa cells.

#### Global analysis of time-resolved fluorescence anisotropy data

Our global fitting algorithm may also be applied to quantitative readouts of time-lapse live cell imaging experiments where fitting to complex decay models with modest numbers of detected photons is required. One application is the readout of homoFRET using time-resolved fluorescence anisotropy, e.g. [32]–[35], which makes use of the fact that fluorescence excitation is most efficient for radiation polarised parallel to the absorption dipole moment of a fluorophore. The net polarisation of fluorescence emission from an initially randomly orientated ensemble of fluorophores therefore tends to follow that of the excitation radiation as long as the rotational correlation time 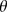 (the timescale over which fluorophore orientations are randomised due to collisions with surrounding molecules) is longer than the fluorescence lifetime. This is typically true for fluorescent proteins (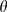
∼30 ns) [4]. When homo-FRET occurs, the emission becomes depolarised [36] due to the angle between the emission dipole of the excited fluorophore and the absorption dipole of the receiving fluorophore. While steady-state anisotropy measurements are sufficient for detecting FRET, time-resolved anisotropy measurements can provide more quantitative information on the fraction of fluorophores undergoing FRET and the FRET rate. Such readouts, however, require the fitting of multiple rotational correlation time components and, for pixel-wise analysis, therefore require more detected photons to achieve a reasonable accuracy than either FLIM or steady-state anisotropy measurements. Global analysis of time resolved anisotropy data has previously been demonstrated across a number of spectral channels [37]–[39], but has not yet been applied to the analysis of image data.

### Demonstration of FLIMfIT

To demonstrate the capabilities of the FLIMfit software, we apply it to the analysis of five different datasets. First, we illustrate the ability of our approach to rapidly analyse a large experimental fluorescence lifetime dataset obtained from a multiwell plate array containing mixtures of dyes using a global double-exponential decay model. Second, we apply a similar analysis approach to the readout of a FRET Rac1 biosensor (mTurquoise/YPET) based on that developed by Hahn et al. [40], [41] in live cells. Third, we demonstrate how this software can enable the use of more complex fitting models to analyse FRET with donors presenting complex fluorescence decay profiles. Fourth, we demonstrate its application to simulated photon-constrained polarisation-resolved TCSPC imaging data of homoFRET between two identical fluorescent proteins with a bi-exponential decay profile and show that it is possible to perform global fitting across the image to successfully recover the lifetimes, fractional contributions and rotational correlation times and compare the results to fitting on a pixel-by-pixel basis. Finally, we apply this approach to demonstrate global analysis of a time series of experimental polarisation-resolved TCSPC images of homo-FRET between PH domains of AKT labelled with EGFP to readout accumulation of PtdIns(3,4,5)P_3_ in the plasma membrane in live mouse embryonic fibroblasts following stimulation with PDGF. We show that it is possible to globally fit this 15 frame time series of 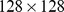
pixel polarisation resolved FLIM images to extract rotational correlation times associated with FRET and rotational motion at sufficiently low photon numbers to be compatible with live cell imaging.

## Methods

### Data Analysis

#### FLIM data model

We first consider the case of a single population of fluorophores with a mono-exponential lifetime 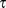 excited by a train of pulses with period 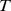
and measured using a system whose instrument response function (IRF) may be described by a function 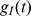. Using the identity for a geometric series, the model fluorescence signal 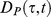 arising from the train of all previous pulses, excluding the current one may be expressed as
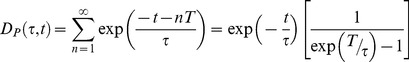
(1)and so the total model decay 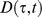, including previous pulses, may be expressed as



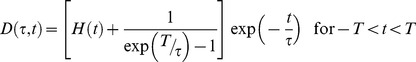
(2)where 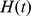 is the Heavyside step function. We note that this expression is equivalent to that derived by Rowley et al. [28]. If a direct measurement of the IRF is available, the measured decay 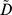 can be expressed as the convolution of the model and the IRF,



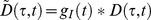
(3)In some cases it is not practical or possible to directly measure the IRF. In this case a measurement of a mono-exponential reference dye with lifetime 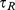, denoted 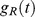, may be used in place of an IRF using the delta-function convolution method [27]. In this case, the model decay 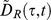 may be expressed as
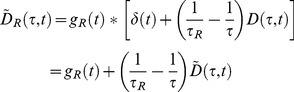
(4)


Using the commutivity of convolution, the model measured intensity decay 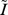 from a mixed population of fluorophores with lifetimes 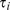 whose amplitudes are given by 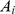 may be expressed as
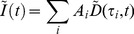
(5)


We use the tilde to indicate that a decay model has been convolved with the IRF.

#### ECFP FRET data model

We describe a model accounting for FRET when ECFP is used as a donor, accounting for the bi-exponential nature of ECFP. The efficiency of FRET transfer between a donor and acceptor fluorophore is given by [42]

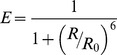
(6)where 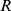 is the distance between the donor and acceptor and the Förster distance 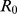 [4] is defined by
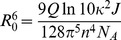
(7)where 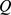 is the fluorescence quantum yield of the donor alone, 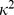 is the dipole orientation factor, n is the refractive index of the medium, 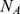 is Avogadro's number and 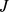 is the overlap integral between the donor emission spectrum and the acceptor excitation spectrum.

ECFP [29], [30] has two lifetime components which are associated with distinct chromophore conformations. If the overall structure and spectra of two conformations are similar [29], it is reasonable to assume that 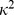, 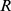 and 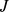 will be approximately equal for the two conformations in a given FRET system and so the Förster distances for the two conformations will be proportional to their respective donor-only quantum yields, which in turn will be proportional to their lifetimes 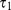 and 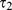 [43]–[45] such that
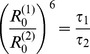
(8)where 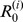 is the Förster distance for the 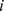 ^th^ component. Although these conditions are not strictly fulfilled by ECFP, which has been reported to present two conformations with slightly different emission spectra [46], it is a useful simplification that is more realistic than the assumption of a monoexponential donor decay profile. Accordingly, we can then express the FRET efficiency of the second component, 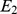, in terms of the FRET efficiency of the first, 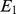, and the two lifetimes by combing Equations 6 and 8 and using the assumption that 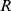 is invariant between the conformations



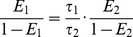
(9)

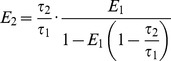
(10)


The two conformations exist in a slow equilibrium [46] and so we may assume that, for a given system, the relative contributions of the two conformations, 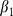 and 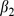, in a non-FRET state will be the same as their relative contributions in the FRET state. The non-FRET and FRET states may then be respectively associated with the decay profiles 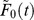 and 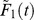


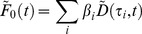
(11)

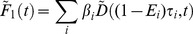
(12)where 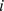 indexes the different conformations. For a bi-exponential donor these two decay profiles are characterised by four parameters: the donor lifetimes 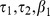 and 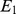 since 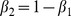 and 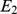 is defined by Equation 10. A model for a mixture of non-FRET and FRET states may then be written
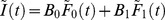
(13)where 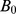 and 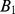 are the amplitudes of the non-FRET and FRET populations respectively.

#### Time-resolved anisotropy data model

In the case of polarisation resolved data we must consider the anisotropy of the fluorophores. Considering a population of identical, randomly oriented fluorophore it may be shown [4] that the fluorescence intensity 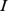 polarised at an angle 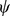 to the excitation is given by
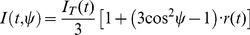
(14)where 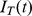 is the total fluorescence intensity decay, which may generally be expressed as a multi-exponential decay with lifetimes 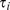
and pre-exponential factors 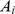, and 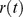 is the time resolved anisotropy. For convenience we define 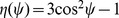. If the time resolved anisotropy follows a multi-exponential decay with correlation times 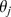 and initial anisotropy contributions 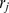, the model becomes



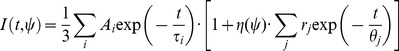
(15)The total initial anisotropy is defined as 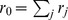. Accounting for a measured instrument response function and contributions from incomplete decays, the model measured decay may be expressed in terms of a sum over one or more convolved single exponential decay functions 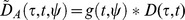 where 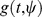 is the instrument response function measured at polarisation angle 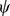.
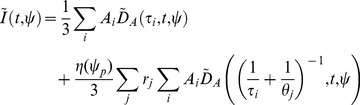
(16)


The instrument response functions may differ between polarisation angles if measurements are made with a polarising beam splitter and two different detectors.

#### Instrument and background light

In addition to light from the sample, the signal may be corrupted by unwanted background light. This background generally takes one of three forms

1. a time-independent background, e.g. from room light or detector dark noise, that may be accounted for by a constant value 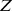

2. scattered light from the excitation source that is not fully blocked by the emission filter, that may be accounted for by including a contribution 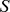 that is proportional to the IRF
3. background fluorescence, e.g. from instrument or fibre-optic cable autofluorescence when using UV excitation, that may be accounted for by measuring the time-dependent background fluorescence, 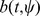, which may have a spatially-varying intensity 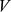 in the absence of a sample and including this contribution in the model.

The model including stray light may be written
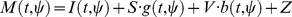
(17)



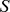
, 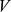 and 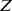 may be included as either local or global parameters depending on whether they are expected to vary from pixel to pixel or to be invariant across the image. These parameters may be fitted but where possible should be measured using data acquired from the instrument with control samples in the absence of the fluorescent objects of interest, e.g. imaging buffer only.

#### Global analysis using partitioned variable projection

To perform global fitting across a large number of decay profiles we use partitioned variable projection [24]. The model shown in Equation 17 may be expressed as the linear sum of a number of nonlinear functions. For global analysis, the nonlinear functions are assumed to be constant across the data set and so the total intensity may be written as
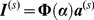
(18)where 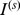 is a vector containing the model decay at the 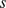 ^th^ pixel, 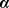 is a vector of nonlinear parameters that are constant across the dataset, 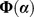 is a matrix whose columns contain the nonlinear functions included in the model and 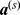 is a vector of linear parameters at the 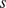 ^th^ pixel. The nonlinear functions will vary depending on the fitting problem specified and may include the exponential decay functions or background components. For example, in the case of a FLIM data set with a bi-exponential decay and a spatially-varying scattered excitation light component, the nonlinear matrix becomes
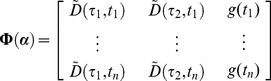
(19)while the linear components are



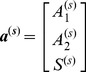
(20)In another example, for the case of a polarisation resolved data set measured at two angles, 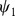 and 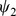, with a mono-exponential fluorescence intensity decay profile with lifetime 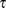 and a bi-exponential anisotropy decay profile with rotational correlation times 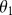 and 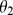 a constant background, the nonlinear matrix becomes



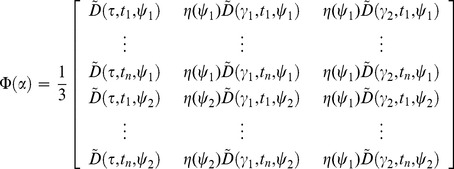
(21)where we define 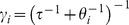 for clarity. The linear components are then



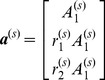
(22)Having expressed the model in a generalised form, the weighted residual between the model and data to be minimised may be written as
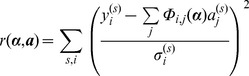
(23)where 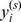 is the decay measured for the 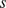 ^th^ pixel at the 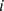 ^th^ time/polarisation point, 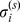 is the estimated error on the 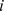 ^th^ measurement and 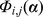 refers to the 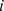 ^th^ measurement in the 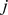 ^th^ column of 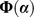. We discuss our approach for estimating 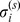 to weight the residual function below.

We may rewrite Equation 23 in matrix form
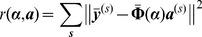
(24)where 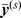 is a column vector of the data measured for the 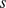 ^th^ pixel weighted by the estimated error and 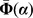 is the decay matrix weighted by the estimated error. Note that it is possible to express the residual as the sum of independent calculations for each pixel since the linear parameters for a given pixel are determined exclusively by the data for that pixel. For a given set of nonlinear parameters, the magnitude of the residual vector is minimised when 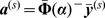, where 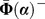 is the symmetric generalized inverse of 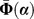. Therefore the linear variables may be eliminated entirely from the residual after [24]



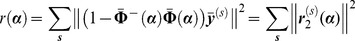
(25)The quantity 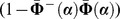 is denoted 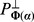 and can be calculated by matrix decomposition of 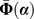
using the QR method [47]. Continuing the notation used by [24], 
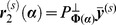
 and is known as the variable projection of 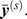 We now have an objective function that can be expressed purely in terms of the nonlinear parameters and so have reduced our minimisation parameter space considerably. Our variable projection implementation is based on a modified version of the VARP2 code by LeVerque [48].

#### Data weighting

A critical factor in non-linear fitting is the choice of data weighting. For Poisson distributed data the variance 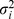 is equal to the expected value of the data. It is well known that two common weighting approaches, Neymann weighting, 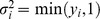 and Pearson weighting, 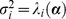, give biased estimators of the true function parameters [49]. Kim and Seok [50] recently presented a systematic investigation of the statistical properties of several common estimator functions. They estimated the bias and variance of the estimators by linearising the gradient of the objective function around the true parameter values;The gradient should of course be zero at the minimum point. As previously observed they showed that Neymann and Pearson weighting yield relatively efficient but biased estimators. They noted that equal weighting, 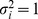, gives an unbiased estimator but with higher variance on the parameter estimate. We use the approximation 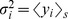, where 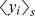 is the measured value for the 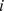 ^th^ time/polarisation point averaged over all pixels. This approach allows the same weighted model function to be used for each pixel and so significantly reduces the computation burden compared to weighting each pixel independently. It is possible to show that this weighting technique produces an unbiased estimator even if the decay in different pixels varies significantly. Consider first the expected value of the derivative of the objective function with respect to the non-linear parameters 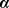 at the true non-linear and linear parameter values, denoted 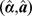. For clarity we write the model function 
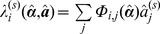
 and make use of Equation 24.
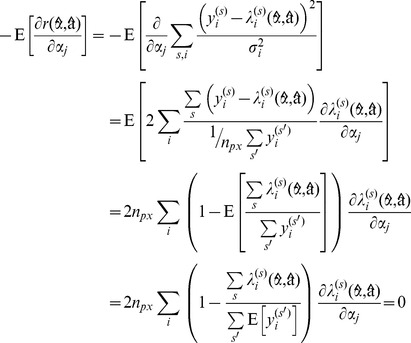
(26)


In the last equality we have used the fact that at the true function minimum, 
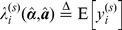
 by definition. The expected value of the derivative with respect to the linear parameters at a given pixel 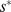 will depend only on the data points for that pixel, since 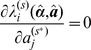 for 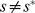.
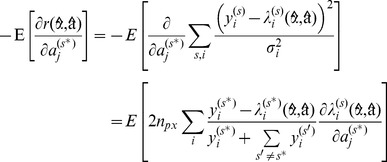
(27)


For a global fitting problem with a moderate number of pixels 
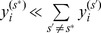
 so the dependence of the average decay, and therefore the weighting, on any given data point is negligible. Using this approximation,
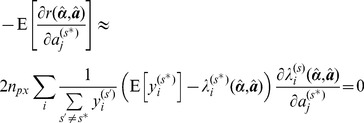
(28)


Therefore average weighting yields an unbiased estimator of the true parameters even if there is a range of disparate lifetime components present across the image.

#### Nonlinear minimization

Nonlinear minimisation of the residual defined by Equation 25 with respect to the parameters 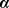 is performed using a modified Levenberg-Marquart (LM) routine derived from the Minpack library [51]. The LM algorithm is a widely used trust-region modification of the traditional Gauss-Newton optimisation algorithm [52]. For fast and stable convergence using the LM algorithm, we use analytical rather than numerical derivatives when calculating the Jacobian matrix of 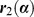. We use the approximation of Kaufman to obtain the Jacobian of the projected residuals [24]

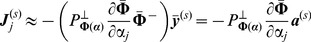
(29)where 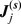 is the derivative of the residual for the 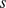 ^th^ pixel with respect to the 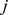 ^th^ nonlinear variable.

#### Multithreaded implementation

**Figure 1:**
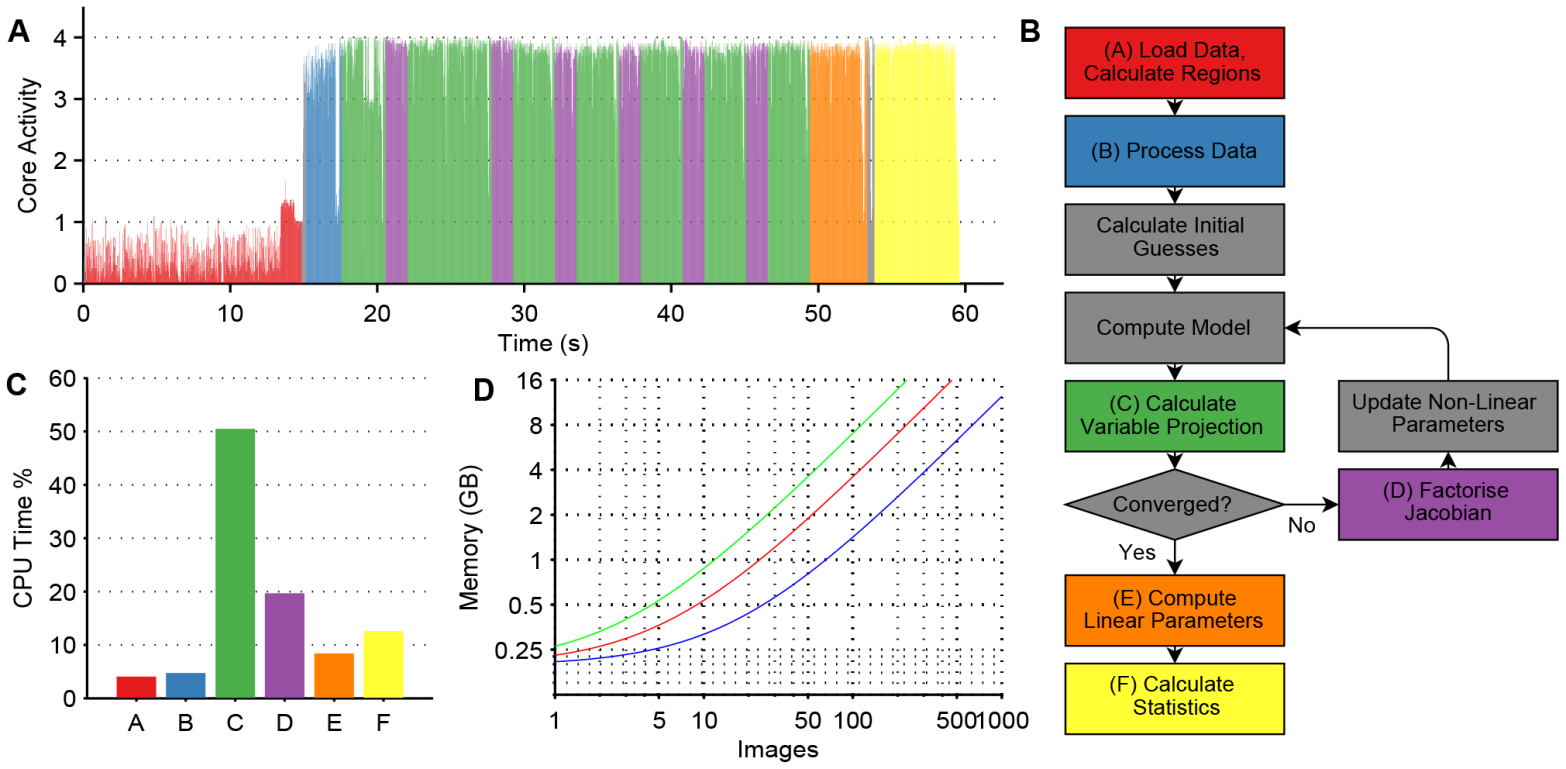
Profiling of the CPU and memory requirements of the algorithm. (A) Fractional core activity of the four cores while performing the fit in Experiment 1, colour coded by algorithm stage as shown in the flowchart in (B) which shows the main stages of the algorithm. (C) CPU time spent on the different algorithm stages in Experiment 1. (E) Memory requirements for a global fit against image number for (blue) a five 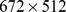
frame time gated FLIM dataset, (red) a 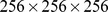
TCSPC dataset and (green) a 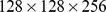 two channel polarisation resolved dataset. Numbers exclude the memory required for the MATLAB runtime engine (300 MB).

The computationally intensive sections of the fitting algorithms are implemented in multi-threaded C++ to exploit the increasing prevalence of multi-core CPUs. A schematic flow chart of the main sections is shown in Figure 1B. The initial loading of data from disk is performed by a single thread loading consecutive images into a circular buffer while a number of worker threads operating in parallel apply the user selected thresholds to calculate image masks and the number of pixels within each region. This part of the algorithm is limited by the data transfer rate from disk and would benefit from use of a solid state drive and/or disk array. The images are stored in virtual memory for the next stage of the processing; if there is enough memory they will be retained in main memory, otherwise they will be reloaded as required. The data is then transformed, first applying any smoothing and background subtraction specified and then applying the image masks calculated earlier. The portions of the data within the image masks are then copied into main memory. Removing the requirement to store only the included portions of the images in memory provides a significant reduction in required memory when analysing images of sparsely seeded cells, since a large fraction of the image is dark. This transformation is calculated in parallel with worker threads transforming images independently.

The computation time of the fitting process is dominated by the variable projection that calculates the residuals at each iteration and the calculation and the factorisation of the Jacobian. The computation of the model functions does not factor significantly, as it is only calculated once per iteration for the entire dataset, rather than for each pixel. The variable projection is calculated in parallel with each thread computing the projection for independent pixels. Since for even a modest global problem there are many more pixels than threads this will scale well to any realistic number of CPU cores in a shared memory architecture.

We use a parallel hybrid Householder-Givens transform approach to factorise the Jacobian across multiple cores based on the approach of Cunha et al. [53]. The pixels are divided evenly between threads. Each thread calculates the QR decomposition of the rows of the Jacobian in blocks of up to 1,024 rows by Householder reflection, combining the resultant matrices in turn using Givens rotations. The rows of the Jacobian are computed on demand, eliminating the requirement to store the whole Jacobian before factorisation. This block size was chosen to minimse the storage requirements without significantly affecting the performance. Once the Jacobian rows for all pixels have been factorised, the resultant matrices from each thread are merged in turn.

After the fit, the linear parameters are calculated by back-substitution in parallel for each pixel and the mean, standard deviation, median, and interquartile range are calculated for each fitted region. These statistics are calculated across each image in parallel. Results are only stored for thresholded pixels, i.e. complete images are constructed for display on demand.

The program is compiled for both 32- and 64-bit platforms. The 64-bit version is able to overcome the 2 GB memory limit imposed by 32-bit Windows and can use the full memory capacity available in modern workstations. All fitting reported here was performed on using an Intel Core i7 870 quad-core processor clocked at 2.93 GHz with 8 Gb of main memory. For convenience we have implemented a graphical user interface (GUI) in MATLAB (Mathworks, MA, USA) that allows the user to load data from multiple sources, to easily modify the fitting parameters and data pre-processing settings, to apply segmentation and to generate summary statistics and false colour maps of the fitted parameters. The source code and compilation instructions for the software used in this paper are provided in File S1. The latest version of the source code and binary executables are available online [54].

To profile the core utilisation by stage in the algorithm, we used the Visual Studio concurrency analysis tool (Microsoft, USA). Note that this tool has a small overhead which increases the overall fitting time when profiling.

#### Memory utilisation

The memory requirements of the conventional LM algorithm are dominated by the storage of the data (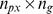), stored as single precision float point, the transformed residuals (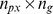), the Jacobian (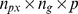), and the results of the fit (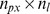) stored as double precision due to their use in intermediate calculations. Here, 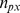 is the number of pixels in the fit, 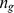 the number of time gates or bins, 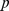 the number of non-zero derivatives of the non-linear functions and 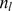 the number of linear parameters in the fit. For a bi-exponential model, 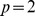 while for a FRET model with a bi-exponential donor, 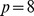. Typically, LM algorithms use Householder reflections [55] to factorise the Jacobian to determine the step direction, which requires storing the entire Jacobian. The use of the hybrid Householder-Givens algorithm for Jacobian factorisation eliminates the requirement to store the complete Jacobian. This reduces the memory requirements by 70–90% compared to the standard Householder algorithm. This also nearly eliminates the dependence of the memory utilisation on the model complexity, leaving only the additional storage required for the results of the fit.

#### Generating initial parameter estimates

To generate initial estimates of the nonlinear parameters to be determined, all the decay profiles in the global data set are binned and the mean lifetime 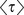 is estimated. For multi-exponential fits the initial estimates for the lifetime components are linearly spaced between 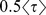 and 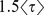, with the smallest and largest lifetimes set equal to those values. These parameters were found to give good convergence over a wide range of lifetimes. When calculating the mean lifetime, data before the peak of the IRF is discarded and the time points are shifted such that the time is zero at the peak of the IRF. For TCSPC data, the mean photon arrival time 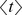 over the acquisition window 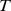 is used, which is given by
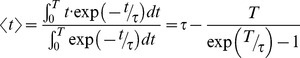
(30)


We calculate the mean lifetime by applying a recursive correction to the mean arrival time to obtain an estimate of the mean lifetime 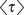 following the methodology of Isenberg and Dyson [56]. We use three iterations of the Newton-Raphson method to solve Equation 30. Defining 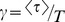, the update step is
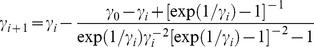
(31)where 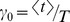. We found that three iterations was sufficient to give a bias of less than 1% in the estimated mean arrival time for all values of 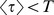.

For time-gated data, the linearised least squares determination method [57] is used to determine the mean lifetime, which is given by
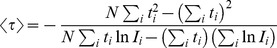
(32)where 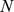
is the number of gates, and 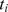 and 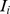 the time and intensity of the 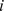 ^th^ gate respectively.

#### Estimating confidence intervals on globally fitted parameters

To calculate confidence intervals for the globally fitting parameters we used full support plane analysis [4], [58], [59]. Briefly, for each fitted parameter an optimisation is performed to determine the amount by which the parameter must change to produce a statistically significant change in the 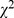. At each step the new parameter value is held constant while the other parameters are refitted. This process is repeated to find the upper and lower confidence limits on all parameters. We use the TOMS algorithm 748 for root finding [60], implemented in the Boost C++ library, to find the required parameter value. The statistically significant change required, 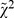, is determined using the F statistic
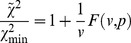
(33)where 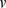 is the number of free parameters in the fit, 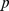 is the required confidence interval, and 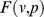 is the F statistic for the required values of 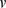 and 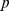. In the present analysis we use 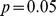 to estimate the 95% confidence limits on the parameters and have presented the larger of the lower and upper confidence intervals. We note that the confidence intervals represent the confidence based on statistical uncertainty and will not account for any biases due to, for example, model inaccuracies.

### Sample Preparation

#### Experiment 1: Multiwell plate rhodamine dye lifetime unmixing

Dye mixtures of Rhodamine B and Rhodamine 6G were prepared by dissolving pure dye powder into spectroscopic grade methanol and then subsequent dilution in MilliQ to give a final 10 µM solution of each dye. The Rhodamine B and Rhodamine 6G solutions were mixed in the following ratios; Columns 1,2; 0∶1, Columns 3,4; 1∶4, Columns 5,6; 2∶3, Columns 7,8; 3∶2, Columns 9,10; 4∶1, Columns 11,12; 1∶0.

#### Cell preparation

All cells were maintained in Dulbecco’s modified Eagle’s medium (DMEM) (Gibco, USA) supplemented with 10% fetal calf serum (Gibco, USA), 0.5% penicillin/streptomycin and 4 mM L-glutamine (growth media). Cells were maintained at 37°C, 5% CO_2_ and grown until 80–90% confluent in T75 flasks (Corning, USA) before passaging using Trypsin-EDTA to detach the cells. For imaging fixed cells in multiwell plates, cells were fixed by incubation with 4% paraformaldehyde for fifteen minutes and washed twice with phosphate buffered saline (PBS).

#### Experiment 2: Multiwell plate FRET assay of inhibition of Rac1-Pak1 interaction

COS-7 cells (ECACC, cat. no. 87021302) were transfected with a 2∶1 ratio of mTurquoise-Rac1 and YPet-PBD plasmid DNA [40], [41] at a final concentration of 2 µg/µl by electroporation using an Amaxa Nuclofector II, program W-001, seeded in 96 well plates (µclear, Greiner) and allowed to settle overnight in culture medium. The cells were starved in DMEM supplemented with 0.5% FCS for three hours. Cells were then incubated with varying concentrations of the PAK inhibitor IPA-3 [61] (1,1′-Disulfanediyldinaphthalen-2-ol, Sigma-Aldrich) for one hour. Cells were stimulated with 100 ng/ml Epidermal Growth Factor (EGF) for fifteen minutes and then fixed.

#### Experiment 3: Multiwell plate FRET assay of inhibition of HIV-1 Gag aggregation

HeLa cells (ECACC, cat. no. 93021013) were seeded in 96 well plates 24 hours prior to transfection. Immediately before transfection the cells were washed in PBS and the growth media replaced with Optimem 1 reduced serum media (Gibco, USA). Transfections were performed using Lipofectamine 2000 (Invitrogen). Transfection mixes were prepared following manufacturer’s instructions using a total of 150 ng of plasmid DNA per well with a 2∶1 ratio of lipids to DNA. The transfection mixes were left on the cells for 6 hours. Cells were then washed in PBS and the media replaced with growth media. The addition of NMT inhibitor doses was performed at the same time as the lipofection, with the NMT inhibitor also being diluted into the Optimem 1 used to seed the cells prior to transfection. Transfections were then carried out as described above. The cells were fixed after 24 hours.

#### Experiment 5: Live cell time-resolved anisotropy imaging of AKT-PH accumulation

Immortalised Mouse Embryonic Fibroblasts (MEFs, as described in [41]) were transfected with 3 µg/µl EGFP-AKT-PH plasmid DNA by electroporation using an Amaxa Nuclofector II, program A-023, seeded onto glass bottom dishes (MatTek, MA, USA) and allowed to settle overnight in culture medium. Before imaging the cells were starved in Hanks Balanced Salt Solution for three hours.

### Time-gated Imaging of Multiwell Plate Data

Samples were excited using a fibre-laser pumped supercontinuum source (Fianium UK Ltd, SC400-6) with a repetition rate of 60 MHz or a frequency doubled, Ti:Sapphire laser with an 80 MHz repetition rate. Images were recorded using an Orca ER2 (Hamamatsu, Japan) with 2×2 binning, providing 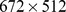 pixels. To account for the IRF, finely sampled reference measurements were made at the same excitation and emission wavelengths as used for imaging using 20 µM DASPI in MilliQ purified water (Millipore, USA) for ECFP or mTurquoise measurements and 20 µM Erythrosin B in MilliQ for Rhodamine B/Rhodamine 6G measurements. Fixed cells were imaged in PBS at room temperature.

#### Experiment 1: Multiwell plate rhodamine dye lifetime unmixing

Imaging was performed using the automated Nipkow spinning-disk (CSU-X, Yokogawa, Japan) based FLIM multiwell plate system described in [22] with a 40x CFI PLAN Fluor ELWD 0.60 NA objective (Nikon, Tokyo, Japan). The sample was illuminated using the supercontinuum source with a 465/30 nm excitation filter and a 525/50 nm emission filter. For each field of view (FOV), six time-gated images with a gate-width of 2000 ps and integration times of 1 s per gate were recorded. Three FOV were recorded per well. An oval segmentation mask with ∼42% coverage was applied to the images to approximate the coverage observed with dense cell data.

The relative contribution to the fluorescence decay of each dye depends on the quantum yield and spectral characteristics of the dyes. We fitted a decay profile from a pure sample of each dye to a single exponential model to find the pre-exponential factor 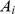, which then allowed us to determine the pre-exponential factor per micromole of dye, 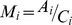, where 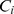 is the molar concentration of each dye in its pure solution. We calculated the expected relative contributions 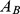 and 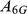 of a mixture of Rhodamine B and Rhodamine 6G with respective molar concentrations 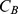 and 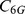 using
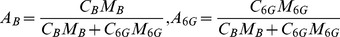
(34)


#### Experiment 2: Multiwell plate FRET assay of inhibition of Rac1-Pak1 interaction

Imaging was performed using an automated widefield FLIM multiwell plate system with a 40x LUCPlanFLN 0.6 NA objective (Olympus, Japan). The sample was excited at 435 nm using the frequency doubled Ti:Sapphire laser and emission recorded with a 483/32 nm filter. Five time-gated images were acquired for each field of view (FOV) with a temporal gate-width of 1000 ps and integration times of 1 s per gate. Eight FOV were recorded per well. Fluorescence intensity images of the acceptor were recorded using a mercury fluorescence lamp with a 545/30 nm excitation filter and 610/75 nm emission filter.

#### Experiment 3: Multiwell plate FRET assay of inhibition of HIV-1 Gag aggregation

Imaging was performed using the automated Nipkow spinning-disk based FLIM multiwell plate system described in [23] with a 40x LUCPlanFLN 0.6 NA objective (Olympus, Japan). The sample was excited using the supercontinuum source with a 434/17 nm excitation filter and emission recorded with a 483/35 nm filter. Seven time-gated images were recorded for each field of view (FOV) with a temporal gate width of 3000 ps and integration times of 2.3 s per gate. Four FOV were recorded per well.

### Time-resolved Anisotropy Imaging Using Tcspc

#### Experiment 5: Live cell time-resolved anisotropy imaging of AKT-PH accumulation

Time-resolved fluorescence anisotropy imaging was performed using a confocal scanning microscope (TCS SP5, Leica Microsystems). The sample was excited at 465 nm using a frequency doubled Ti:Sapphire laser. The polarisation state of the excitation light was controlled using a cube polariser and a half-wave plate. GFP emission was recorded using a 528/38 nm emission filter. The confocal pinhole was set to one Airy unit. Emission light was split into parallel and perpendicular components using a polarising beam splitter cube and recording simultaneously using hybrid photomultipliers (HPM-100-40, Becker & Hickl, GmbH). Photon counting was performed using a (SPC830, Becker & Hickl, GmbH) TCSPC board. A 40× 0.75NA objective was used and 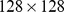 pixel images recorded with 256 time-bins over a measurement period of 12.5 ns with a total integration time per image of 60 s. An IRF was recorded using excitation light scattered from a coverslip with edge-overlapping filters and the half-wave plate rotated until the peak of both channels was approximately equal. The g-factor representing the polarisation response of the instrument [4] of the system was determined using a 20 µM Rhodamine 6G sample in MilliQ water assuming that the residual anisotropy should be zero since Rhodamine 6G has a short (∼200 ps) anisotropy decay. The relative magnitudes of the IRF in each channel were adjusted so as to compensate for their relative sensitivities as determined by the measured g-factor. Time lapse TCSPC images were recorded every two minutes and the cell was stimulated with 50 ng/ml PDGF after six minutes. In total, 15 images of 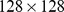 pixels were recorded. Due to the low signal levels, a 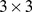
mean smoothing kernel was applied to each the image representing each time-bin of the data by convolution to reduce the error in the local parameter estimates.

### Data Simulation

#### Experiment 4: Simulated TR-FAIM homo-FRET data

Polarisation resolved TCSPC data of fluorophores with a bi-exponential fluorescence decay profile undergoing homo-FRET leading to a bi-exponential anisotropy decay profile was simulated in MATLAB with a time window of 12.5 ns for a simulated pulse repetition rate of 80 MHz. The model, before accounting for incomplete decays and a simulated instrument response function takes the form
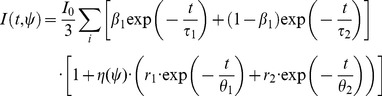
(35)where 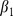 is the fractional contribution of the long component of the intensity decay and 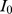 is the peak intensity. The data was simulated for two detectors polarised parallel and perpendicular to the excitation light, i.e. 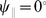 and 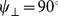. Using Equation 14, 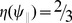 and 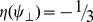. The model was convolved with a Gaussian IRF with a full width half maximum of 150 ps. The lifetimes of the fluorescence decay components were set to be 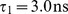 and 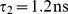 with a fractional contribution of the long component 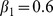. The rotational correlation times were set to 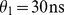 and 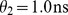, i.e. of the order expected for a fluorescent protein fusion construct undergoing homo-FRET. The 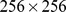 pixel simulated image was split into three regions with initial anisotropy contribution of the short component 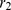
set to 0.1, 0.2 and 0.3. In all three regions the total initial anisotropy 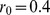. The initial intensity 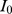 was set such that there were on average 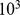
total integrated counts in each pixel and Poissonian noise was added to each decay using the MATLAB function poissrnd. These simulation parameters were chosen to approximate realistic values for cell imaging data, with the lifetimes selected to be similar to those of common cyan fluorescent protein variants such as ECFP and Cerulean [62]. In common with Experiment 5, a 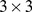 mean smoothing kernel was applied to each the image representing each time-bin of the data by convolution.

### Image Segmentation

In Experiment 2, automatic image segmentation was performed on the integrated intensity image and a corresponding acceptor intensity image to identify individual cells expressing both donor and acceptor. To identify cells above the background, a size-tuned nonlinear top-hat (nTh) transform [63] was applied to the mTurquoise integrated intensity image. This method applies the pixelwise transform
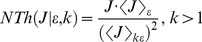
(36)to the integrated intensity image 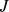 where 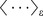 denotes averaging with a square mask of width 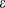. This transformation locally enhances bright pixels in region of approximately size 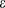
surrounded by a dim region of size 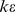. The mask radius 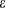
was set to the approximate size of cells, 200 pixels, while relative background radius 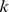 was set to 2. The transformed image was then thresholded at a value of 0.1. Since the COS-7 cells were relatively densely seeded regions identified by this method often contained more than one cell. A marker based watershed segmentation routine [64] was applied to identify individual cells from the thresholded regions, exploiting the fact the mTurquoise fluorescence is higher in the cell nucleus in this cell system. Briefly, ultimate erosion of the thresholded image was calculated by iterative erosion to identify the peaks associated with the bright cell nuclei. Markers separated by fewer than 20 pixels were assumed to belong to the same nucleus and so were merged. The remaining peaks were used as markers in a watershed transform, which identifies the boundaries between objects by ‘flooding’ the regions around the markers [65]. The cell regions were then filtered according to three criteria. Regions with a total area of fewer than 4000 pixels were rejected since this is significantly smaller than the average cells size and likely to be associated with cell debris. Since we are only interested in cells expressing both mTurquoise-Rac1 and YPet-PBD, the regions were then thresholded based on the fluorescence intensity in the acceptor channel. Regions below the threshold were rejected.

## Results

### Global Fitting of Multiwell Plate Fluorescence Lifetime Data

#### Experiment 1: Multiwell plate rhodamine dye lifetime unmixing

**Figure 2:**
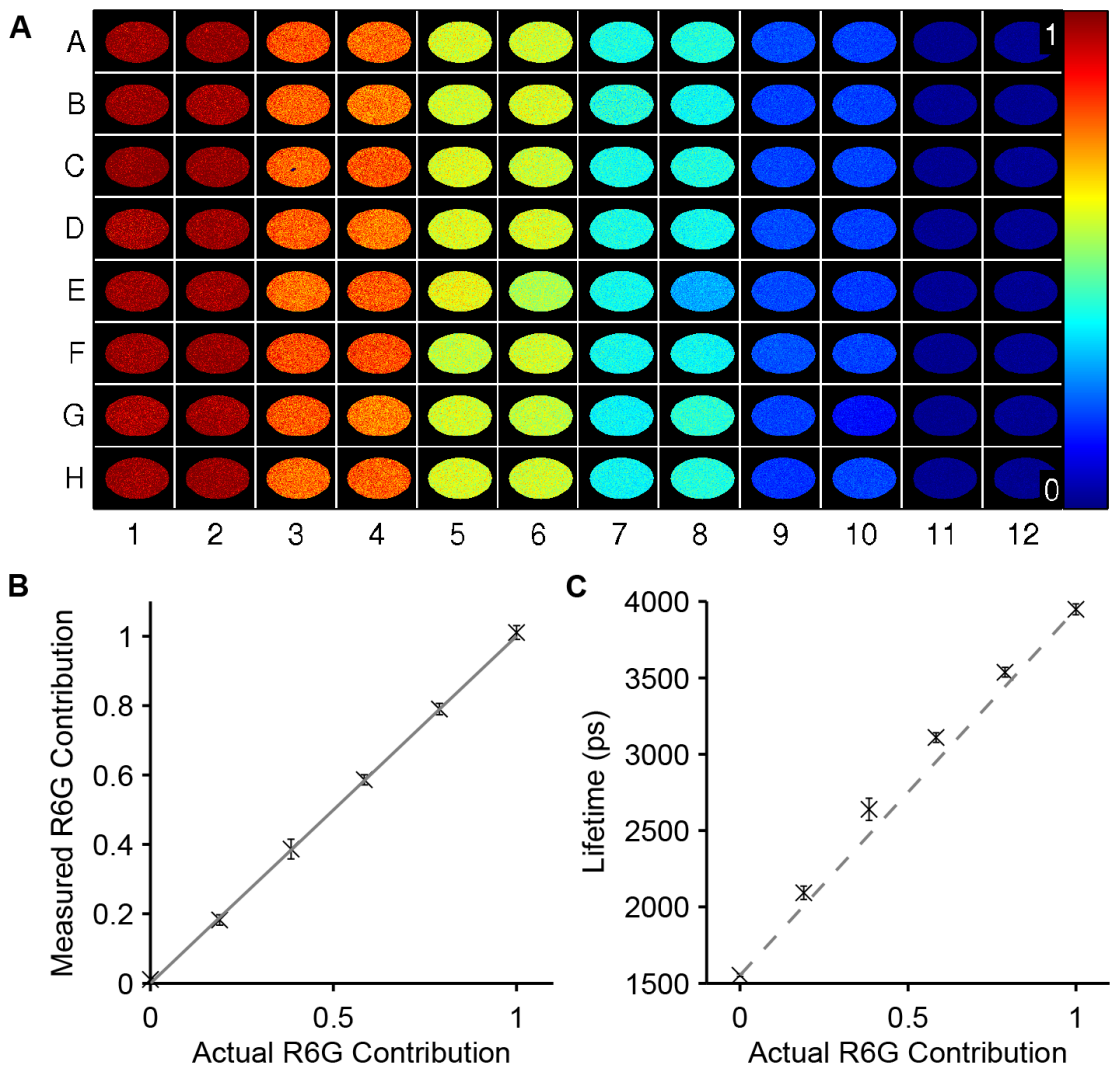
Global analysis of a multiwell plate with varying concentrations of fluorescent dyes. Global analysis was applied to a multiwell plate with varying concentrations of the fluorescent dyes Rhodamine B and Rhodamine 6G using a bi-exponential model. The relative concentration of Rhodamine 6G reduces across pairs of columns as described in the text. The dataset contains four fields FOV per well. A) plate map showing the measured fractional contribution of Rhodamine 6G for a representative FOV in each well. B) plot of the actual Rhodamine 6G contribution against measured contribution (crosses). C) plot of measured lifetime using a single exponential fit against actual Rhodamine 6G concentration. This dataset was collected as part of a previous study [22].

To illustrate global fitting of large datasets we analysed a 96 well plate arrays containing a mixture of Rhodamine 6G and Rhodamine B in water at six different relative concentrations. These are both fluorescent dyes reported [66] to present mono-exponential decays with lifetimes 4.08 and 1.52 ns respectively. FLIM images consisting of six time-gated images were acquired for each FOV and four FOV were acquired per well. This produced 384 FLIM images and a total of 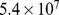 pixels/decays. The dataset was fitted globally to a bi-exponential model and the analysis time, including loading the data, was 54 seconds and required 4.7 GB of main PC memory. The fitted lifetimes were 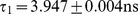 and 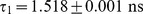, in reasonable agreement with literature [66]. Figure 2A shows a plate map of the contribution of Rhodamine 6G across the plate and a plot of the measured contribution against the actual contribution. The CPU utilisation during the fitting process is shown in Figure 1A, colour coded by algorithm stage. For comparison, a pixel-wise single exponential decay analysis was also performed, which took 219 seconds to analyse the entire dataset.

#### Experiment 2: Multiwell plate FRET assay of inhibition of Rac1-Pak1 interaction

To demonstrate the ability of the fitting software to analyse biological multiwell plate FLIM FRET data, we performed global fitting on a FRET dataset assaying the effect of the p21-activated kinase (Pak1) inhibitor IPA-3 [61] on the interaction between Rac1 and Pak1 in COS-7 cells. Rac1 [67] is a small GTPase involved in cell growth and motility, among other processes, that is known to bind Pak1 in its active form [68], [69]. Here we used a modified version of the intermolecular FRET biosensor, FLAIR [40], [41], consisting of a mTurquoise-Rac1 construct and YPet conjugated to the p21-binding domain of Pak1 (PDB). mTurquoise is a fluorescent protein with a spectral profile similar to that of ECFP but which exhibits a mono-exponential decay profile [70]. This enables the FLIM FRET analysis to be performed using a bi-exponential decay model where the long lifetime and short lifetimes may be associated with donor only and interacting populations respectively.

**Figure 3:**
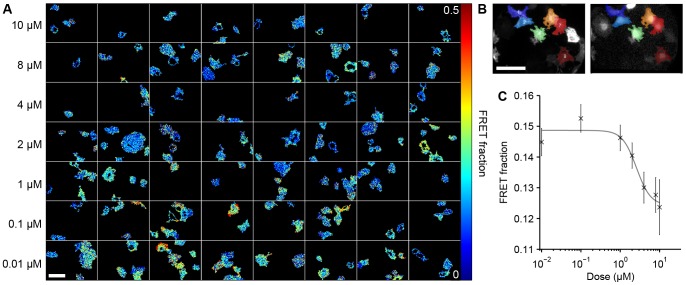
Global analysis of an IPA-3 dose-response dataset modulating the interaction between Rac1 and Pak1. Global analysis was applied to a multiwell plate dose-response dataset showing the effect of the inhibitor IPA-3 on interaction between Rac1 and Pak1 using an mTurquoise variant of the FLAIR biosensor in COS-7 cells stimulated with EGF. A) representative images from each inhibitor concentration showing distribution of fraction undergoing FRET. B) examples of automatic image segmentation with *(left)* donor intensity and *(right)* acceptor images shown in grey-scale with coloured segmented cell regions overlaid. *C*) plot of fraction of donor molecules undergoing FRET against IPA-3 concentration, averaged across segmented cells with fitted dose-response curve. Error bars indicate 95% confidence intervals on average FRET fraction over segmented cells at each dose. White scale bar represents 100 µm.

The dataset comprising 394 FOV over a range of doses of IPA-3 was globally fitted to a bi-exponential decay model. The images were segmented as described in the methods section. In total, 1,508 cell regions were identified. In a random subset of fifty fields of views, the automatic segmentation was compared against manual segmentation in order to determine the segmentation accuracy. In each field of view we counted the number of correctly identified cells (TP), false positives (FP) and false negatives (FN). We could then calculate the hit rate, defined here as TP/(TP+FN). We also assessed the quality of the region boundaries and counted regions containing more than one cell (M) and the number of regions that missed portions of the true cell extent (IN). In that subset, we found TP = 137 cells, FP = 5 cells (3.6%), FN = 8 cells (5.5%), giving a hit rate of 87%. Additionally M = 7 (5.1%) and IN = 14 (10.2%). An example of the segmentation results are shown in Figure 3B. Since the background fluorescence from the plastic plates is not negligible at the wavelengths used to excite mTurquoise, we included a time varying background in the fitting model that was acquired from a measurement of the fluorescence decay profile of a well filled with PBS. We fitted to a double exponential model across all FOV and the analysis took 32 seconds and required 2 Gb of memory. The globally fitted lifetimes were 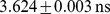 and 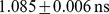, corresponding to the unbound and bound mTurquoise-Rac1 states respectively. Figure 3A shows representative false colour maps of the interacting fraction and Figure 3C shows the fractional contribution of the short lifetime associated with the interacting population as a function of IPA-3 concentration, where the fractional contributions are averaged over each segmented cell. We fitted a dose-response curve to this data using nonlinear fitting to the Hill equation. This gave an EC50 value of 2.59 µM, which is close to the value reported in the literature of 2.5 µM [71].

#### Experiment 3: Multiwell plate FRET assay of inhibition of HIV-1 gag aggregation

We have also demonstrated the ability of our fitting software to account for the complex decay profile of ECFP as a FRET donor by applying it to a FLIM-FRET assay of Gag protein aggregation in HeLa cells [22], [23] and its response to the NMT inhibitor DDD85646 [72]. HIV-1 Gag proteins are responsible for enabling the assembly of nascent HIV-1 virions at the cell membrane [73] and produce virus like particles (VLPs) even in the absence of other viral proteins and enzymes. They are therefore often used as a model system for the late stages in the HIV-1 lifecycle. VLP formation may be monitored using a FRET aggregation assay by co-transfecting cells with Gag proteins stochastically labelled with donor (ECFP) and acceptor (EYFP) fluorophores that undergo FRET when colocalised in Gag protein aggregates. The NMT inhibitor prevents the formation of VLPs and was used to obtain dose response curves of the Gag aggregation. In our previous study [23], the analysis was restricted to a single exponential decay model that provided a semi-quantitative readout. For further quantitative analysis, however, it would be useful to determine the relative fractions of Gag monomers and VLP-bound Gag proteins and an estimate of the average FRET efficiency of proteins in VLPs by fitting to a more complex decay model. Accordingly, we analysed an inhibitor dose-response dataset using global fitting to a bi-exponential donor FRET model using the assumption that the fluorophores can be divided into a first population of non-FRETing monomers and a second population of VLP-bound oligomers undergoing FRET. The dataset contained cells at nine different dose concentrations with a total of 385 FOV, for which the analysis took 7.3 seconds using 400 Mb of main memory.

**Figure 4:**
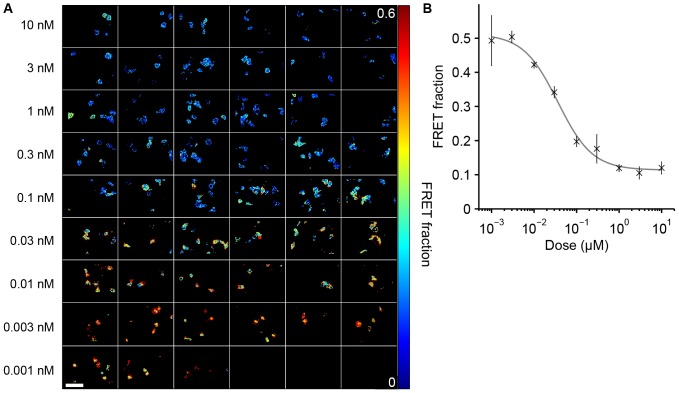
Global analysis of an NMT inhibitor dose-response dataset modulating Gag aggregation. Global analysis was performed across a multiwell plate dataset with HeLa cells expressing ECFP-Gag and EYFP-Gag with increasing levels of a NMT inhibitor using a bi-exponential donor FRET model. A) representative images from each inhibitor dose showing distribution of fraction Gag-CFP undergoing FRET. B) plot of fraction of Gag-CFP undergoing FRET against inhibitor concentration, averaged across wells with fitted dose-response curve. Error bars indicate 95% confidence intervals across wells. This dataset was collected as part of a previous study [23]. White scale bar represents 100 µm.

Table 1 shows the globally fitted parameters and Figure 4 shows the fraction of Gag molecules undergoing FRET as a function of inhibitor dose with representative false colour maps of the VLP-bound fraction. The fraction of Gag population undergoing FRET varies from 50% at low concentrations of inhibitor to just over 10% at high concentrations. On closer examination of the images, we can see that in the membrane regions of many cells exposed to low concentrations of the inhibitor, the FRET population fraction is close to 100% at the membrane where we expect aggregation to be highest. We fitted a dose-response curve to the average FRET population across wells using nonlinear fitting to the Hill equation, giving an EC50 value of 0.037 µM.

**Table 1:** Parameters from global fitting of a dose-response dataset using an NMT inhibitor with HeLa cells expressing ECFP-Gag and EYFP-Gag using a bi-exponential donor FRET model.

	τ (ns)	B	E	τ *_FRET_* (ns)
**Component 1**	3.493±0.005	0.6114±0.003	0.55±0.02	*1.55±0.05*
**Component 2**	0.961±0.003	*0.3856±0.003*	*0.25±0.01*	*0.72±0.01*

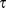
 represents the lifetimes of the donor-only decay and 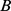 represents the fractional contribution of each component. 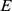 represents the FRET efficiency for each component as defined in Equations 9–13. All three parameters are determined globally. 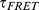 represents the fluorescence lifetime of the FRET population calculated from the fitted parameters and is therefore shown in italics.

### Global Fitting of Time-Resolved Anisotropy Homo-fret Data

#### Experiment 4: Simulated time-resolved anisotropy homo-FRET data

**Figure 5:**
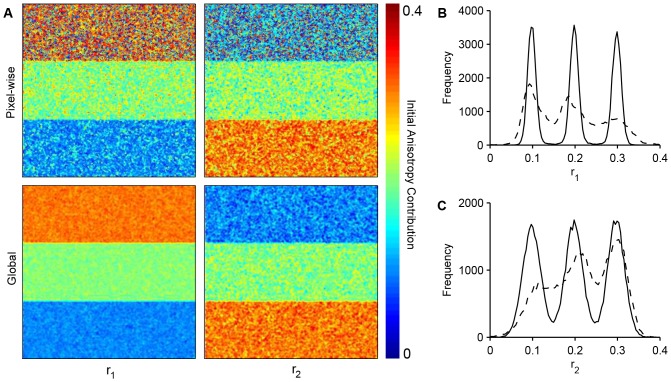
Global and pixel-wise analysis of simulated TCSPC polarisation resolved image data. Simulated polarisation resolved TCSPC data was generated with fluorescence lifetimes 3.0 and 1.2 ns and rotational correlation times of 30 ns and 1.0 ns. The simulated data was generated with the total initial anisotropy set to 0.4 across the image with the initial anisotropy contribution of the short component equal to 0.1, 0.2 and 0.3 in three bands from top to bottom across the image. (A) False colour images of the recovered initial anisotropy contribution for the long (left) and short (right) correlation time components analysed pixel-wise (top) and with global fitting (bottom); Histograms of estimates of the initial anisotropy contribution of the (B) long and (C) short correlation time components analysed pixel-wise (dashed lines) and with global fitting (solid lines).

Polarisation resolved TCSPC data simulated as described in the methods section was fitted on a pixel-wise and global basis to the polarisation resolved model described in Equation 35. In each case, the lifetimes and fractional contributions of both the fluorescence and anisotropy decays were left free. Table 2 shows the fit parameters obtained for quantities that were are invariant across the initial simulated image and Figure 5 shows false colour maps and histograms of the recovered initial anisotropy contributions. Using global fitting, the true values of the spatially invariant parameters are recovered within the standard deviation and the different initial anisotropy regions are clearly distinguishable. When fitting pixel-wise, however, the errors on the spatially invariant parameters are significantly larger, particularly for the rotational correlation times. The large uncertainty in the correlation times is reflected in the initial anisotropy contributions, which show little correlation with the true values.

**Table 2:** Parameters used for simulation of polarisation resolved TCSPC data of a fluorescent protein undergoing homo-FRET and fitted parameters using global and pixel-wise fitting.

	*τ*_1_ (ns)	*τ*_2_ (ns)	*β*_1_	*θ*_1_ (ns)	*θ*_2_ (ns)
**Simulated**	3.0	1.2	0.6	30.0	1.0
**Global Fit**	2.998±0.002	1.198±0.003	0.600±0.001	30.00±0.28	0.999±0.004
**Pixel-wise** **Fit**	2.78±0.30	1.04±0.27	0.68±0.13	7.42±7.74	1.33±0.43

The mean and standard deviations are calculated over 10 independent datasets.

#### Experiment 5: Live cell time-resolved anisotropy imaging of AKT-PH accumulation

We applied the global fitting approach to study experimental homo-FRET data of EGFP tagged AKT-PH, providing an intensity-independent readout of PtdIns(3,4,5)P_3_ accumulation in response to PDGF stimulation. The pleckstrin homology domain of the AKT protein kinase (AKT-PH) is known to bind selectively to PtdIns(3,4,5)P_3_ and has been used to monitor accumulation of this signalling molecule in the plasma membrane [74] by translocation. As previously noted [75], it is often difficult to quantify this membrane localisation and a number of factors such as changes in membrane shape, e.g. due to ruffling, can lead to changes in intensity independent of translocation. FRET has previously been used to report on phosphoinositide translocation and it has been suggested that PtdIns(3,4,5)P_3_ localises in microdomains within the plasma membrane [76], [77] where the local density may be high enough to allow efficient FRET between bound PH-domains. Van der Wal et al. [78] used this approach to report on the membrane translocation of the PH domain of phospholipase C-δ1 (PLC δ1-PH), which binds PtdIns(3,4)P_2_ (PIP_2_). They co-transfected cells with CFP-PLC δ1-PH and YFP-PLC δ1-PH and observed that upon hydrolysis of PIP_2_ the PH domains translocate to the membrane and a reduction in FRET is observed.

Here we acquired a 15 frame dataset that was globally fitted to a mono-exponential fluorescence intensity decay model with lifetime 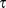 and two rotational correlation times 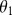 and 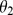 with respective initial anisotropy contributions 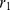 and 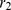. The model before convolution and before accounting for incomplete decays took the form
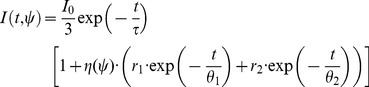
(37)


The fitting process took 7.2 seconds using 550 Mb of main memory. The resulting globally estimated lifetime of EGFP was 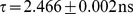, i.e. close to a previously reported lifetime of 2.39 ns for the same construct [79]. The rotational correlation times were estimated to be 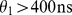 and 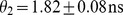, which we associate with the rotational motion of the molecules and homo-FRET respectively. Note that we present only a lower bound on 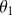 as the 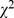 surface is essentially flat for larger values of 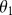 since we are not able to accurately resolve long rotational correlation times due to the shorter fluorescence decay time. The rotational correlation time is much longer than that reported for free EGFP in the cytoplasm, 36 ns [80], suggesting that the rotation of the protein is significantly hindered, consistent with membrane binding.

**Figure 6:**
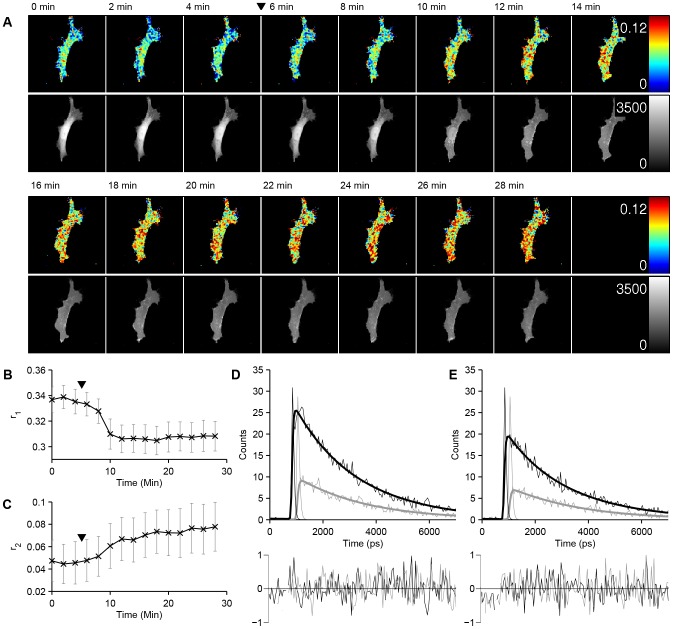
Global analysis of a polarisation resolved homo-FRET TCSPC dataset reading out PtdIns(3,4,5)P_3_ accumulation at the membrane. A MEF transfected with EGFP-AKT-PH was imaged at two minute intervals and stimulated with 50 ng/ml PDGF after 6 minutes (indicated by black triangles). (A, top row) False colour map of the initial anisotropy contribution *r*_2_ associated with homo-FRET over the time course. (A, bottom row) Integrated fluorescence intensity images over the time course. (B, C) Initial anisotropy contributions spatially averaged over the cell: *r_1_* associated with the rotational correlation (B) and *r_2_* associated with homo-FRET (C). Error bars represent the standard deviation across the image. (D,E) Exemplar fluorescence decays from the region indicated by a white triangle in the first (D) and last (E) frame with fit (top) and normalised residuals (bottom). The thin, fainter lines represent the experimental data while the thick, bolder lines represent the fitted model. Black lines represent the parallel component while grey lines represent the perpendicular component. Data are representative of three experiments. White scale bar represents 20 µm.

Figure 6 shows the temporal development of the fitted initial anisotropy contributions 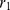
and 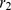 (associated with the rotational correlation time and homo-FRET respectively) following stimulation with EGF. After stimulation, the initial anisotropy contribution of the short rotational component due to FRET, 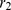 decreases, consistent with an increase in FRET due to accumulation of EGFP tagged AKT-PH at the membrane, and there is a corresponding reduction in the initial anisotropy contribution associated with the rotational correlation time. Consistent with previous observations [74], a translocation of EGFP tagged AKT-PH from the nucleus and cytosol to the membrane is also observed. Note that the decrease in GFP fluorescence intensity over the time course is due to this translocation to the membrane upon stimulation and not to photobleaching, which was measured to change the fluorescence intensity by less than 8%.

## Discussion

### Model Validity

The interpretation of the estimated parameters from this or any other fitting software depends critically on the validity of the model applied. For example if a FRET donor has a significant second decay component but a biexponential model is used to fit the detected signal, then the contribution of the shorter lifetime fitted component, nominally associated with FRET, will be higher to compensate for the second component of the donor alone. This could lead to a systematic overestimate of the FRET population fraction. Even for a donor with a mono-exponential lifetime, recent studies suggest that the approximation of the decay of a population of molecules undergoing FRET by a single exponential decay may dramatically break down under certain circumstances. For example, Vogel et al. [81] recently demonstrated that a short Cerulean-YFP linked construct demonstrates a bi-exponential decay and proposed that this could be due to a distribution of conformations with a variety of chromophore angles and distances, leading to a distribution of FRET efficiencies.

In Experiment 2 we used a global double exponential decay model to analyse data from an mTurquoise YPet FRET pair. This model assumes that mTurquoise exhibits a monoexponential decay, which we believe is reasonable [70]. This model also assumes a narrow distribution of FRET efficiency, which implies a tightly constrained relative orientation of the mTurquoise with respect to the YPet when Rac1 binds PDB. This assumption has not been tested rigorously. If there is significant flexibility of mTurquoise relative to YPet when the constructs are bound, then the absolute values reported by the analysis may not be correct. However, relative changes in the fit parameters will still report changes in the relative binding of the FRET pair.

In the future it may be possible to account for these effects by using a model which, for example, accounts for the ensemble average decay of a population of biosensors with, for example, an isotropic 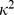 distribution (see Equation 19 in [81]). Using a global analysis approach where the model function need only be computed once per iteration, rather than for each pixel, could enable the practical application and evaluation of such computationally expensive models.

In Experiment 3 we assumed that the fluorophores can be divided into a first population of monomers and a second population of VLP-bound oligomers undergoing FRET. It is thought that Gag packs in a 2D hexagonal structure as VLPs form [82] and therefore all nearest neighbours are likely to have similar distances and orientations. In addition, the close packing may constrain the motion of the fluorophore. While our fitting model is clearly an approximation to the complex underlying situation, it appears to provide a reasonable tool to analyse the Gag oligomerisation. For the purposes of this paper, this experiment and its analysis is intended only to demonstrate the ability of our approach to explore the use of more complex global models that may provide a better approximation to complex fluorescence decay profiles than fitting to a single exponential decay model.

In Experiment 5, we assumed a monoexponential decay for the fluorescence decay of EGFP, which is consistent with previous work showing that the second decay component of EGFP has an amplitude of less than 10% at the wavelength used in this experiment [83]. We then assume that FRET introduces a short single exponential anisotropy decay associated with depolarisation due to homo-FRET in addition to a longer single exponential anisotropy decay associated with the rotational correlation time of the whole protein. This implies, as in Experiment 2, that there is a narrow distribution of FRET efficiencies and hence tightly constrained relative orientation between the FRETing fluorophores. These assumptions have been applied before, e.g. [43], [84], but should be treated with caution and be taken into account when interpreting biological data obtained using them.

### Algorithm Scalability

The memory usage of the algorithm scales linearly with the number of data points for large datasets. Figure 1D illustrates the upper limit of the memory requirement as a function of number of images for a selection of common data dimensions. This is dominated by the storage of the data, residuals and the results. The use of a hybrid Householder-Givens algorithm to factorise the Jacobian means the memory usage is essentially independent of model complexity, with the exception of storage of additional linear parameters. The computational complexity of the algorithm scales linearly with the number of data points. Computationally intensive sections of the algorithm are divided across cores either by pixel or image and so will scale efficiently with increasing cores while the number of cores is smaller than the number of images. Figure 1A shows the computational load of the CPU while fitting the data in Experiment 1. The initial data loading the performance is limited by the speed of the data store. During the fitting process the four cores are nearly fully utilised. The computational complexity of the Levenberg-Marquart algorithm is well known to scale quadratically with number of nonlinear parameters, while the variable projection algorithm scales quadratically with the number of linear parameters.

### Conclusions

We have demonstrated a new global fitting software tool for the analysis of FLIM data based on variable projection. By optimising the memory usage and enabling parallel processing across multiple CPU cores, it is possible to apply global analysis to obtain quantitative information from large multiwell plate or time-series datasets with 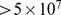
decay profiles using standard PC workstations with analysis times on the order of 1–2 minutes.

We have illustrated the ability of this software to globally fit complex decay models to photon-constrained data, as is typically encountered with live cell imaging using fluorescent proteins. Such data can be fitted more robustly to more complex decay models than is possible with traditional pixel-wise analysis of separate images. In particular, we have applied a FRET model accounting for the bi-exponential nature of donor fluorophores such as ECFP where the FRET efficiencies and relative contributions of the two fluorophore conformations are linked, accounting for their relative quantum yields. Using simulated and experimental data we also demonstrated the potential to apply global fitting to analyse polarisation resolved TCSPC data to determine the anisotropy decay and lifetime parameters associated with homo-FRET. We note that any fitting software should be used with caution and the results will always be limited by the validity of the fitting model. To this end, however, we believe that global analysis tools which enable the fitting of practical experimental fluorescence lifetime data to complex models can be important in improving the biological utility of FLIM and FRET experiments.

We note that the speed of this global fitting approach is sufficient to make it routinely useful, even for large FLIM datasets where previously only non-iterative approaches using simpler decay models were practical. Indeed the ease with which global fitting can now be applied to multiwell plate array or time-series datasets strengthens the case to implement automatic acquisition of such large datasets for biological studies.

Source code and binary executable files for a software package implementing this algorithm, FLIMfit, are available under an open source licence through the Open Microscopy Environment [54].

## Supporting Information

File S1 **Compressed archive of the source code for FLIMfit used in the present study.** Please note that up to date source code and compiled binaries are available from the Open Microscopy website [54].
(ZIP)

## References

[1] Vogel SS, Thaler C, Koushik SV (2006) Fanciful FRET. Science’s STKE 2006. doi:10.1126/stke.3312006re2. 10.1126/stke.3312006re2 16622184

[2] Piston DW, Kremers GJ (2007) Fluorescent protein FRET: the good, the bad and the ugly. Trends Biochem Sci 32: 407–414 doi:10.1016/j.tibs.2007.08.003 17764955 10.1016/j.tibs.2007.08.003

[3] Suhling K, Siegel J, Phillips D, French PMW, Lévêque-Fort S, et al (2002) Imaging the environment of green fluorescent protein. Biophys J 83: 3589–3595 doi:10.1016/S0006-3495(02)75359-9 12496126 10.1016/S0006-3495(02)75359-9 PMC1302434

[4] Lakowicz JR (1999) Principles of Fluorescence Spectroscopy. Second Edi. Kluwer Academic.

[5] Munro I, McGinty J, Galletly N, Requejo-Isidro J, Lanigan PMP, et al (2005) Toward the clinical application of time-domain fluorescence lifetime imaging. J Biomed Opt 10: 051403 doi:10.1117/1.2102807 16292940 10.1117/1.2102807

[6] Sharman KK, Periasamy A, Ashworth H, Demas JN (1999) Error analysis of the rapid lifetime determination method for double-exponential decays and new windowing schemes. Anal Chem 71: 947–952 doi:10.1021/ac981050d 21662765 10.1021/ac981050d

[7] Clayton AHA, Hanley QS, Verveer PJ (2004) Graphical representation and multicomponent analysis of single-frequency fluorescence lifetime imaging microscopy data. J Microsc 213: 1–5 doi:10.1111/j.1365-2818.2004.01265.x 14678506 10.1111/j.1365-2818.2004.01265.x

[8] Digman MA, Caiolfa VR, Zamai M, Gratton E (2008) The phasor approach to fluorescence lifetime imaging analysis. Biophys J 94: L14–6 doi:10.1529/biophysj.107.120154 17981902 10.1529/biophysj.107.120154 PMC2157251

[9] Fereidouni F, Esposito A, Blab GA, Gerritsen HC (2011) A modified phasor approach for analyzing time-gated fluorescence lifetime images. J Microsc 244: 248–258 doi:–10.1111/j.1365–2818.2011.03533.x 21933184 10.1111/j.1365-2818.2011.03533.x

[10] McGinty J, Requejo-Isidro J, Munro I, Talbot CB, Kellett PA, et al (2009) Signal-to-noise characterization of time-gated intensifiers used for wide-field time-domain FLIM. J Phys D: Appl Phys 42: 135103 doi:10.1088/0022-3727/42/13/135103

[11] Rizzo MA, Springer GH, Granada B, Piston DW (2004) An improved cyan fluorescent protein variant useful for FRET. Nat Biotechnol 22: 445–449 doi:10.1038/nbt945 14990965 10.1038/nbt945

[12] Köllner M, Wolfrum J (1992) How many photons are necessary for fluorescence-lifetime measurements? Chem Phys Lett 200: 199–204 doi:10.1016/0009-2614(92)87068-Z

[13] Miyawaki A, Griesbeck O, Heim R, Tsien RY (1999) Dynamic and quantitative Ca2+ measurements using improved cameleons. P Natl A Sci 96: 2135–2140 doi:10.1073/pnas.96.5.2135 10.1073/pnas.96.5.2135 PMC26749 10051607

[14] Beechem JM (1992) Global analysis of biochemical and biophysical data. Methods in Enzymology. Elsevier, Vol. 210: 37–54 doi:10.1016/0076-6879(92)10004-W 10.1016/0076-6879(92)10004-w 1584042

[15] Verveer PJ, Squire A, Bastiaens PIH (2000) Global analysis of fluorescence lifetime imaging microscopy data. Biophys J 78: 2127–2137 doi:10.1016/S0006-3495(00)76759-2 10733990 10.1016/S0006-3495(00)76759-2 PMC1300804

[16] Pelet S, Previte M, Laiho L, So P (2004) A fast global fitting algorithm for fluorescence lifetime imaging microscopy based on image segmentation. Biophys J 87: 2807–2817 doi:10.1529/biophysj.104.045492 15454472 10.1529/biophysj.104.045492 PMC1304699

[17] Barber PR (2005) Global and pixel kinetic data analysis for FRET detection by multi-photon time-domain FLIM. Proc SPIE: 171–181. doi:10.1117/12.590510.

[18] Barber PR, Ameer-Beg SM, Gilbey J, Carlin LM, Keppler M, et al (2009) Multiphoton time-domain fluorescence lifetime imaging microscopy: practical application to protein–protein interactions using global analysis. Journal of The Royal Society Interface 6: S93–S105 doi:10.1098/rsif.2008.0451.focus

[19] Visser AJWG, Apanasovich VV, Van Stokkum IHM, Borst JW, Mullen KM, et al. (2007) Fluorescence Lifetime Imaging Microscopy (FLIM) Data Analysis with TIMP. J Stat Softw 18. Available: http://ideas.repec.org/a/jss/jstsof/18i08.html. Accessed 2 August 2010.

[20] Esposito A, Dohm CP, Bähr M, Wouters FS (2007) Unsupervised fluorescence lifetime imaging microscopy for high content and high throughput screening. Mol Cell Proteomics 6: 1446–1454 doi:10.1074/mcp.T700006-MCP200 17510051 10.1074/mcp.T700006-MCP200

[21] McGhee EJ, Owen DM, Talbot CB, Neil MAA, McGinty J, et al (2008) High speed unsupervised fluorescence lifetime imaging confocal multiwell plate reader for high content analysis. Journal of Biophotonics 1: 514–521 doi:10.1002/jbio.200810054 19343677 10.1002/jbio.200810054

[22] Kumar S, Alibhai D, Margineanu A, Laine R, Kennedy G, et al (2011) FLIM FRET technology for drug discovery: automated multiwell-plate high-content analysis, multiplexed readouts and application in situ. Chemphyschem 12: 609–626 doi:10.1002/cphc.201000874 21337485 10.1002/cphc.201000874 PMC3084521

[23] Alibhai D, Kelly DJ, Warren S, Kumar S, Margineau A, et al.. (2012) Automated fluorescence lifetime imaging plate reader and its application to Förster resonant energy transfer readout of Gag protein aggregation. Journal of Biophotonics. doi:10.1002/jbio.201200185. 10.1002/jbio.201200185 PMC3660788 23184449

[24] Golub G, Pereyra V (2003) Separable nonlinear least squares: the variable projection method and its applications. Inverse Problems 19: R1–R26 doi:10.1088/0266-5611/19/2/201

[25] Christensen TM, Hurn AS, Lindsay K (2008) The devil is in the detail: hints for practical optimisation. Economic Analysis and Policy 38: 345–368.

[26] Gauduchon P, Wahl P (1978) Pulsefluorimetry of tyrosyl peptides. Biophys Chem 8: 87–104 doi:10.1016/0301-4622(78)85026-1 647105 10.1016/0301-4622(78)85026-1

[27] Zuker M, Szabo AG, Bramall L, Krajcarski DT, Selinger B (1985) Delta function convolution method (DFCM) for fluorescence decay experiments. Rev Sci Instrum 56: 14 doi:10.1063/1.1138457

[28] Rowley MI, Barber PR, Coolen ACC, Vojnovic B (2011) Bayesian analysis of fluorescence lifetime imaging data. Proceedings of SPIE. Vol. 7903. p. 790325. doi:10.1117/12.873890.

[29] Demachy I, Ridard J, Laguitton-Pasquier H, Durnerin E, Vallverdu G, et al (2005) Cyan fluorescent protein: molecular dynamics, simulations, and electronic absorption spectrum. J Phys Chem B 109: 24121–24133 doi:10.1021/jp054656w 16375404 10.1021/jp054656w

[30] Hyun Bae J, Rubini M, Jung G, Wiegand G, Seifert MHJ, et al (2003) Expansion of the Genetic Code Enables Design of a Novel “Gold” Class of Green Fluorescent Proteins. J Mol Biol 328: 1071–1081 doi:10.1016/S0022-2836(03)00364-4 12729742 10.1016/s0022-2836(03)00364-4

[31] Berney C, Danuser G (2003) FRET or no FRET: a quantitative comparison. Biophys J 84: 3992–4010 doi:10.1016/S0006-3495(03)75126-1 12770904 10.1016/S0006-3495(03)75126-1 PMC1302980

[32] Clayton AHA, Hanley QS, Arndt-Jovin DJ, Subramaniam V, Jovin TM (2002) Dynamic fluorescence anisotropy imaging microscopy in the frequency domain (rFLIM). Biophys J 83: 1631–1649 doi:10.1016/S0006-3495(02)73932-5 12202387 10.1016/S0006-3495(02)73932-5 PMC1302260

[33] Gautier I, Tramier M, Durieux C, Coppey J, Pansu RB, et al (2001) Homo-FRET microscopy in living cells to measure monomer-dimer transition of GFP-tagged proteins. Biophys J 80: 3000–3008 doi:10.1016/S0006-3495(01)76265-0 11371472 10.1016/S0006-3495(01)76265-0 PMC1301483

[34] Bader AN, Hofman EG, Van Bergen en Henegouwen PM, Gerritsen HC (2007) Imaging of protein cluster sizes by means of confocal time-gated fluorescence anisotropy microscopy. Opt Express 15: 6934–6945 doi:10.1364/OE.15.006934 19547008 10.1364/oe.15.006934

[35] Thaler C, Koushik S V, Puhl HL, Blank PS, Vogel SS (2009) Structural rearrangement of CaMKIIalpha catalytic domains encodes activation. Proc Natl Acad Sci US A 106: 6369–6374 doi:10.1073/pnas.0901913106 10.1073/pnas.0901913106 PMC2669344 19339497

[36] Berberan-Santos MN, Valeur B (1991) Fluorescence depolarization by electronic energy transfer in donor–acceptor pairs of like and unlike chromophores. The Journal of Chemical Physics 95: 8048 doi:10.1063/1.461285

[37] Beechmam J, Knutson J, Brand L (1986) Global analysis of multiple dye fluorescence anisotropy experiments on proteins. Biochem Soc Trans 14: 832–835. Available: http://m.biochemsoctrans.org/bst/014/0832/0140832.pdf. Accessed 12 March 2013. 10.1042/bst0140832 3781080

[38] Crutzen M, Ameloot M, Boens N, Negri RM, De Schryver FC (1993) Global analysis of unmatched polarized fluorescence decay curves. J Phys Chem 97: 8133–8145 doi:10.1021/j100133a005

[39] Zheng W, Li D, Qu JY (2010) Monitoring changes of cellular metabolism and microviscosity in vitro based on time-resolved endogenous fluorescence and its anisotropy decay dynamics. J Biomed Opt 15: 037013 doi:10.1117/1.3449577 20615042 10.1117/1.3449577

[40] Itoh RE, Kurokawa K, Ohba Y, Yoshizaki H, Mochizuki N, et al (2002) Activation of Rac and Cdc42 Video Imaged by Fluorescent Resonance Energy Transfer-Based Single-Molecule Probes in the Membrane of Living Cells. Mol Cell Biol 22: 6582–6591 doi:10.1128/MCB.22.18.6582 12192056 10.1128/MCB.22.18.6582-6591.2002 PMC135619

[41] Martins M, Warren S, Kimberley C, Margineanu A, Peschard P, et al (2012) Activity of phospholipase C epsilon contributes to chemotaxis of fibroblasts towards platelet-derived growth factor. J Cell Sci 125: 5758–5769 doi:10.1242/jcs.110007 22992460 10.1242/jcs.110007 PMC3575709

[42] Clegg RM (1995) Fluorescence resonance energy transfer. Curr Opin Biotechnol 6: 103–110 doi:10.1016/0958-1669(95)80016-6 7534502 10.1016/0958-1669(95)80016-6

[43] Borst JW, Laptenok SP, Westphal AH, Kühnemuth R, Hornen H, et al (2008) Structural changes of yellow Cameleon domains observed by quantitative FRET analysis and polarized fluorescence correlation spectroscopy. Biophys J 95: 5399–5411 doi:10.1529/biophysj.107.114587 18790855 10.1529/biophysj.107.114587 PMC2586569

[44] Albaugh S, Steiner RF (1989) Determination of distance distribution from time domain fluorometry. J Phys Chem 93: 8013–8016 doi:10.1021/j100361a011

[45] Czuper A, Gryczynski I, Kuśba J (2007) Förster energy transfer from nonexponentially decaying donors. J Photochem Photobiol B 87: 200–208 doi:10.1016/j.jphotobiol.2007.04.003 17537640 10.1016/j.jphotobiol.2007.04.003

[46] Seifert MHJ, Ksiazek D, Azim MK, Smialowski P, Budisa N, et al (2002) Slow Exchange in the Chromophore of a Green Fluorescent Protein Variant. J Am Chem Soc 124: 7932–7942 doi:10.1021/ja0257725 12095337 10.1021/ja0257725

[47] Horn RA, Johnson CR (1990) Matrix Analysis. Cambridge University Press. doi:10.1002/zamm.19870670330.

[48] Golub, G H; LeVeque R (1979) Extensions and uses of the variable projection algorithm for solving nonlinear least squares problems. Proc. Army Numerical Analysis and Computers Conf. Washington, DC. 1–12.

[49] Turton DA, Reid GD, Beddard GS (2003) Accurate Analysis of Fluorescence Decays from Single Molecules in Photon Counting Experiments. Anal Chem 75: 4182–4187 doi:10.1021/ac034325k 14632133 10.1021/ac034325k

[50] Kim J, Seok J (2013) Statistical properties of amplitude and decay parameter estimators for fluorescence lifetime imaging. Opt Express 21: 6061 doi:10.1364/OE.21.006061 23482174 10.1364/OE.21.006061

[51] Moré J, Garbow B, Hillstrom K (1980) User guide for MINPACK-1. Argonne National Laboratory Argonne, IL.

[52] Nocedal J, Wright S (1999) Numerical Optimization. Springer. doi:10.1007/978-0-387-40065-5.

[53] Cunha RD da, Patterson JC, Becker D (2002) New Parallel (Rank-Revealing) QR Factorisation Algorithms. Euro-Par 2002 Parallel Processing. Springer Berlin Heidelberg. 677–686. doi:10.1007/3-540-45706-2_94.

[54] Open Microscopy Environment (n.d.). Available: http://www.openmicroscopy.org/site/products/partner/flimfit. Accessed 1 September 2012.

[55] Householder AS (1958) Unitary Triangularization of a Nonsymmetric Matrix. J ACM 5: 339–342 doi:10.1145/320941.320947

[56] Isenberg I, Dyson RD (1969) The analysis of fluorescence decay by a method of moments. Biophys J 9: 1337–1350 doi:10.1016/S0006-3495(69)86456-8 5353139 10.1016/S0006-3495(69)86456-8 PMC1367635

[57] Chang C-W, Mycek M-A (2010) Enhancing precision in time-domain fluorescence lifetime imaging. J Biomed Opt 15: 056013 doi:10.1117/1.3494566 21054107 10.1117/1.3494566 PMC2966491

[58] Straume M, Fraiser-Cadoret S, Johnson M (2002) Least-squares analysis of fluorescence data. In: Lakowicz JR, editor. Topics in Fluorescence Spectroscopy. Boston: Kluwer Academic Publishers, Vol. 2: 177–239 doi:10.1007/b112907

[59] Motulsky H, Christopoulos A (2004) Fitting Models to Biological Data Using Linear and Nonlinear Regression : A Practical Guide to Curve Fitting. Oxford University Press. doi:10.1002/sim.2181.

[60] Alefeld GE, Potra FA, Shi Y (1995) Algorithm 748; enclosing zeros of continuous functions. ACM Transactions on Mathematical Software 21: 327–344 doi:10.1145/210089.210111

[61] Deacon SW, Beeser A, Fukui JA, Rennefahrt UEE, Myers C, et al (2008) An isoform-selective, small-molecule inhibitor targets the autoregulatory mechanism of p21-activated kinase. Chem Biol 15: 322–331 doi:10.1016/j.chembiol.2008.03.005 18420139 10.1016/j.chembiol.2008.03.005 PMC4353635

[62] Sarkar P, Koushik S V, Vogel SS, Gryczynski I, Gryczynski Z (2009) Photophysical properties of Cerulean and Venus fluorescent proteins. J Biomed Opt 14: 034047 doi:10.1117/1.3156842 19566339 10.1117/1.3156842 PMC2754229

[63] Santos AF, Zaltsman AB, Martin RC, Kuzmin A, Alexandrov Y, et al (2008) Angiogenesis: an improved in vitro biological system and automated image-based workflow to aid identification and characterization of angiogenesis and angiogenic modulators. Assay Drug Dev Technol 6: 693–710 doi:10.1089/adt.2008.146 19035850 10.1089/adt.2008.146

[64] Malpica N, De Solórzano CO, Vaquero JJ, Santos A, Vallcorba I, et al (1997) Applying watershed algorithms to the segmentation of clustered nuclei. Cytometry 28: 289–297 doi:10.1002/(SICI)1097-0320(19970801)28:4<289::AID-CYTO3>3.0.CO;2–7 9266748 10.1002/(sici)1097-0320(19970801)28:4<289::aid-cyto3>3.0.co;2-7

[65] Beucher S (1991) The Watershed Transformation Applied To Image Segmentation. Scanning Microscopy International. 299–314. doi:10.1.1.24.5229.

[66] Magde D, Rojas GE, Seybold PG (1999) Solvent Dependence of the Fluorescence Lifetimes of Xanthene Dyes. Photochem Photobiol 70: 737–744 doi:10.1111/j.1751-1097.1999.tb08277.x

[67] Didsbury J, Weber R, Bokoch G, Evans T, Snyderman R (1989) rac, a novel ras-related family of proteins that are botulinum toxin substrates. J Biol Chem 264: 16378–16382 2674130

[68] Knaus UG (1998) Structural Requirements for PAK Activation by Rac GTPases. J Biol Chem 273: 21512–21518 doi:10.1074/jbc.273.34.21512 9705280 10.1074/jbc.273.34.21512

[69] Bokoch GM (2003) Biology of the p21-activated kinases. Annu Rev Biochem 72: 743–781 doi:10.1146/annurev.biochem.72.121801.161742 12676796 10.1146/annurev.biochem.72.121801.161742

[70] Goedhart J, Van Weeren L, Hink MA, Vischer NOE, Jalink K, et al (2010) Bright cyan fluorescent protein variants identified by fluorescence lifetime screening. Nat Methods 7: 137–139 doi:10.1038/nmeth.1415 20081836 10.1038/nmeth.1415

[71] Coleman N, Kissil J (2012) Recent advances in the development of p21-activated kinase inhibitors. Cell Logist 2: 132–135 doi:10.4161/cl.21667 23162744 10.4161/cl.21667 PMC3490963

[72] Goncalves V, Brannigan JA, Thinon E, Olaleye TO, Serwa R, et al (2012) A fluorescence-based assay for N-myristoyltransferase activity. Anal Biochem 421: 342–344 doi:10.1016/j.ab.2011.10.013 22051857 10.1016/j.ab.2011.10.013 PMC3863716

[73] Ono A (2010) HIV-1 assembly at the plasma membrane. Vaccine 28 Suppl 2B55–9 doi:10.1016/j.vaccine.2009.10.021 20510745 10.1016/j.vaccine.2009.10.021 PMC2879345

[74] Servant G (2000) Polarization of Chemoattractant Receptor Signaling During Neutrophil Chemotaxis. Science (80-) 287: 1037–1040 doi:10.1126/science.287.5455.1037 10.1126/science.287.5455.1037 PMC2822871 10669415

[75] Várnai P, Balla T (2006) Live cell imaging of phosphoinositide dynamics with fluorescent protein domains. Biochim Biophys Acta 1761: 957–967 doi:10.1016/j.bbalip.2006.03.019 16702024 10.1016/j.bbalip.2006.03.019

[76] Lasserre R, Guo X-J, Conchonaud F, Hamon Y, Hawchar O, et al (2008) Raft nanodomains contribute to Akt/PKB plasma membrane recruitment and activation. Nat Chem Biol 4: 538–547 doi:10.1038/nchembio.103 18641634 10.1038/nchembio.103

[77] Gao X, Lowry PR, Zhou X, Depry C, Wei Z, et al (2011) PI3K/Akt signaling requires spatial compartmentalization in plasma membrane microdomains. Proc Natl Acad Sci US A 108: 14509–14514 doi:10.1073/pnas.1019386108 10.1073/pnas.1019386108 PMC3167518 21873248

[78] Van der Wal J, Habets R, Várnai P, Balla T, Jalink K (2001) Monitoring agonist-induced phospholipase C activation in live cells by fluorescence resonance energy transfer. J Biol Chem 276: 15337–15344 doi:10.1074/jbc.M007194200 11152673 10.1074/jbc.M007194200

[79] König I, Schwarz JP, Anderson KI (2008) Fluorescence lifetime imaging: association of cortical actin with a PIP3-rich membrane compartment. Eur J Cell Biol 87: 735–741 doi:10.1016/j.ejcb.2008.02.002 18375014 10.1016/j.ejcb.2008.02.002

[80] Swaminathan R, Hoang CP, Verkman AS (1997) Photobleaching recovery and anisotropy decay of green fluorescent protein GFP-S65T in solution and cells: cytoplasmic viscosity probed by green fluorescent protein translational and rotational diffusion. Biophys J 72: 1900–1907 doi:10.1016/S0006-3495(97)78835-0 9083693 10.1016/S0006-3495(97)78835-0 PMC1184383

[81] Vogel SS, Nguyen TA, Van der Meer BW, Blank PS (2012) The impact of heterogeneity and dark acceptor states on FRET: implications for using fluorescent protein donors and acceptors. PLoS One 7: e49593 doi:10.1371/journal.pone.0049593 23152925 10.1371/journal.pone.0049593 PMC3496711

[82] Briggs JAG, Simon MN, Gross I, Kräusslich H-G, Fuller SD, et al (2004) The stoichiometry of Gag protein in HIV-1. Nat Struct Mol Biol 11: 672–675 doi:10.1038/nsmb785 15208690 10.1038/nsmb785

[83] Cotlet M, Hofkens J, Maus M, Gensch T, Van der Auweraer M, et al (2001) Excited-State Dynamics in the Enhanced Green Fluorescent Protein Mutant Probed by Picosecond Time-Resolved Single Photon Counting Spectroscopy. J Phys Chem B 105: 4999–5006 doi:10.1021/jp003813i

[84] Devauges V, Marquer C, Lécart S, Cossec J-C, Potier M-C, et al (2012) Homodimerization of amyloid precursor protein at the plasma membrane: a homoFRET study by time-resolved fluorescence anisotropy imaging. PLoS One 7: e44434 doi:10.1371/journal.pone.0044434 22973448 10.1371/journal.pone.0044434 PMC3433432

