# Supplementary material for: Rapid Global Fitting of Large Fluorescence Lifetime Imaging Microscopy Datasets
Source: PLoS One. 2013 Aug 5;8(8):e70687. doi: 10.1371/journal.pone.0070687 (PMC3734241; doi:10.1371/journal.pone.0070687)
Supplement: File S1 — Compressed archive of the source code for FLIMfit used in the present study. Please note that up to date source code and compiled binaries are available from the Open Microscopy website [54]. (ZIP) [file pone.0070687.s001.zip › FLIMFit 4_3_3 Source/GlobalProcessingFrontEnd/HelperFunctions/GUILayout/layoutHelp/atoz.html]

GUI Layout Toolbox documentation: Index
  


GUI Layout Toolbox 1.12

# Alphabetical index

A -
B -
C -
D -
E -
F -
G -
H -
I -
J -
K -
L -
M
N -
O -
P -
Q -
R -
S -
T -
U -
V -
W -
X -
Y -
Z

|  |  |  |  |  |  |  |  |  |  |  |  |  |  |  |  |  |  |  |  |  |  |  |  |  |  |  |  |  |  |  |  |  |  |  |  |  |  |  |  |  |  |  |  |  |  |  |  |  |  |  |  |  |  |  |  |  |  |  |  |  |  |  |  |  |  |  |  |  |  |  |  |  |  |  |  |  |  |  |  |  |  |  |  |  |  |  |  |  |  |  |  |  |  |  |  |  |  |  |  |  |  |  |  |  |  |  |  |  |  |  |  |  |  |  |  |  |  |  |  |  |  |  |  |  |  |  |  |  |  |  |  |  |  |  |  |  |  |  |  |  |  |  |  |  |  |  |  |  |  |  |  |  |  |  |  |  |  |  |  |  |  |  |  |  |  |  |  |  |  |  |  |  |  |  |  |  |  |  |  |  |  |  |  |  |  |  |  |  |  |  |  |  |  |  |  |  |  |  |  |  |  |  |  |  |  |  |  |  |  |  |  |  |  |  |  |  |  |  |  |  |  |  |  |  |  |  |  |  |  |  |  |  |  |  |  |  |  |  |  |  |  |  |  |  |  |  |  |  |  |  |  |  |  |  |  |  |  |  |  |  |  |  |  |  |  |  |  |  |  |  |  |  |
| --- | --- | --- | --- | --- | --- | --- | --- | --- | --- | --- | --- | --- | --- | --- | --- | --- | --- | --- | --- | --- | --- | --- | --- | --- | --- | --- | --- | --- | --- | --- | --- | --- | --- | --- | --- | --- | --- | --- | --- | --- | --- | --- | --- | --- | --- | --- | --- | --- | --- | --- | --- | --- | --- | --- | --- | --- | --- | --- | --- | --- | --- | --- | --- | --- | --- | --- | --- | --- | --- | --- | --- | --- | --- | --- | --- | --- | --- | --- | --- | --- | --- | --- | --- | --- | --- | --- | --- | --- | --- | --- | --- | --- | --- | --- | --- | --- | --- | --- | --- | --- | --- | --- | --- | --- | --- | --- | --- | --- | --- | --- | --- | --- | --- | --- | --- | --- | --- | --- | --- | --- | --- | --- | --- | --- | --- | --- | --- | --- | --- | --- | --- | --- | --- | --- | --- | --- | --- | --- | --- | --- | --- | --- | --- | --- | --- | --- | --- | --- | --- | --- | --- | --- | --- | --- | --- | --- | --- | --- | --- | --- | --- | --- | --- | --- | --- | --- | --- | --- | --- | --- | --- | --- | --- | --- | --- | --- | --- | --- | --- | --- | --- | --- | --- | --- | --- | --- | --- | --- | --- | --- | --- | --- | --- | --- | --- | --- | --- | --- | --- | --- | --- | --- | --- | --- | --- | --- | --- | --- | --- | --- | --- | --- | --- | --- | --- | --- | --- | --- | --- | --- | --- | --- | --- | --- | --- | --- | --- | --- | --- | --- | --- | --- | --- | --- | --- | --- | --- | --- | --- | --- | --- | --- | --- | --- | --- | --- | --- | --- | --- | --- | --- | --- | --- | --- | --- | --- | --- | --- | --- | --- | --- | --- | --- | --- | --- | --- | --- | --- | --- | --- | --- | --- |
| \|  \|  \| \| --- \| --- \| \| ---   A \| \| \| A complete example \| A complete example (User guide: 8) \| \| About \| What is the GUI Layout Toolbox? (Getting Started: 1) \| \| Acknowledgements \| Acknowledgements (Getting Started: 5) \| \| Advanced maneuvers with panels \| Advanced maneuvers with panels (User guide: 5) \| \| Application design \| A complete example (User guide: 8) \| \| Application structure \| Application structure (User guide: 8.1) \| \| axes \| Positioning Axes (User guide: 2) \| \| Axes inside layouts \| Axes inside layouts (User guide: 2.2) \| \| ---   B \| \| \| Box \| uiextras.HBox (Function reference: 2.1) \| \|  \| uiextras.VBox (Function reference: 2.2) \| \|  \| uiextras.HBoxFlex (Function reference: 2.3) \| \|  \| uiextras.VBoxFlex (Function reference: 2.4) \| \| Boxes \| Boxes (Function reference: 2) \| \| BoxPanel \| uiextras.BoxPanel (Function reference: 1.3) \| \| Button box \| uiextras.HButtonBox (Function reference: 2.5) \| \|  \| uiextras.VButtonBox (Function reference: 2.6) \| \| ---   C \| \| \| Callbacks \| A complete example (User guide: 8) \| \| CardPanel \| uiextras.CardPanel (Function reference: 1.2) \| \| Compiler \| Deploying GUIs using the MATLAB Compiler (User guide: 7) \| \| Context help \| Context help (User guide: 5.1) \| \| createInterface \| createInterface (User guide: 8.2) \| \| ---   D \| \| \| Date \| layoutVersion (Function reference: 4.2) \| \| Default \| Using property defaults (User guide: 4) \| \|  \| uiextras.set (Function reference: 4.4) \| \|  \| uiextras.get (Function reference: 4.5) \| \| Deploying GUIs using the MATLAB Compiler \| Deploying GUIs using the MATLAB Compiler (User guide: 7) \| \| Dock and undock \| Dock and undock (User guide: 5.3) \| \| Draggable dividers \| uiextras.HBoxFlex (Function reference: 2.3) \| \|  \| uiextras.VBoxFlex (Function reference: 2.4) \| \|  \| uiextras.GridFlex (Function reference: 3.2) \| \| ---   E \| \| \| Empty \| uiextras.Empty (Function reference: 4.1) \| \| Enable and Visible \| Enable and Visible (User guide: 3) \| \| Enable example \| Enable example (User guide: 3.1) \| \| Examples \| (Examples) \| \| ---   F \| \| \| Flexible layout \| uiextras.HBoxFlex (Function reference: 2.3) \| \|  \| uiextras.VBoxFlex (Function reference: 2.4) \| \| Folder \| layoutRoot (Function reference: 4.3) \| \| Function reference \| (Function reference) \| \| ---   G \| \| \| Getting Started \| (Getting Started) \| \| Grid \| uiextras.Grid (Function reference: 3.1) \| \| GridFlex \| uiextras.GridFlex (Function reference: 3.2) \| \| Grids \| Grids (Function reference: 3) \| \| GUIDE \| Using layouts inside GUIDE GUIs (User guide: 6) \| \| ---   H \| \| \| HBox \| uiextras.HBox (Function reference: 2.1) \| \| HBoxFlex \| uiextras.HBoxFlex (Function reference: 2.3) \| \| HButtonBox \| uiextras.HButtonBox (Function reference: 2.5) \| \| Help \| Support (Getting Started: 4) \| \| Horizontal button layout \| uiextras.HButtonBox (Function reference: 2.5) \| \| Horizontal layout \| uiextras.HBox (Function reference: 2.1) \| \|  \| uiextras.HBoxFlex (Function reference: 2.3) \| \| ---   I \| \| \| Installation \| Installation (Getting Started: 3) \| \| Installation folder \| layoutRoot (Function reference: 4.3) \| \|  \| \| \|  \| \| \| ---   L \| \| \| Layout basics \| Layout basics (User guide: 1.1) \| \| Layout heirarchies \| Layout heirarchies (User guide: 1.4) \| \| layoutRoot \| layoutRoot (Function reference: 4.3) \| \| layoutVersion \| layoutVersion (Function reference: 4.2) \| \| ---   M \| \| \| MATLAB Compiler \| Deploying GUIs using the MATLAB Compiler (User guide: 7) \| \| Minimize and maximize \| Minimize and maximize (User guide: 5.2) \| \| Minimum sizes \| Sizes and units (User guide: 1.3.1) \| \|  \| \| \| ---   O \| \| \| onListSelection \| onListSelection (User guide: 8.4) \| \| Other functions \| Other functions (Function reference: 4) \| \| ---   P \| \| \| Panel \| uiextras.Panel (Function reference: 1.1) \| \| Panels \| Panels (Function reference: 1) \| \| Position vs OuterPosition \| Position vs OuterPosition (User guide: 2.1) \| \| Positioning Axes \| Positioning Axes (User guide: 2) \| \| Property \| Using property defaults (User guide: 4) \| \|  \| uiextras.set (Function reference: 4.4) \| \|  \| uiextras.get (Function reference: 4.5) \| \|  \| \| \| ---   R \| \| \| Release Notes \| Release Notes (Getting Started: 2) \| \| Root \| layoutRoot (Function reference: 4.3) \| \| Running it \| Running it (User guide: 8.5) \| \| ---   S \| \| \| Scalability \| Scalability (User guide: 8.6) \| \| Setting defaults for all windows \| Setting defaults for all windows (User guide: 4.2) \| \| Setting defaults for one window \| Setting defaults for one window (User guide: 4.1) \| \| Sizes and units \| Sizes and units (User guide: 1.3) \| \| Support \| Support (Getting Started: 4) \| \| ---   T \| \| \| TabPanel \| uiextras.TabPanel (Function reference: 1.4) \| \| Types of layout \| Types of layout (User guide: 1.2) \| \| ---   U \| \| \| uiextras.BoxPanel \| uiextras.BoxPanel (Function reference: 1.3) \| \| uiextras.CardPanel \| uiextras.CardPanel (Function reference: 1.2) \| \| uiextras.Empty \| uiextras.Empty (Function reference: 4.1) \| \| uiextras.get \| uiextras.get (Function reference: 4.5) \| \| uiextras.Grid \| uiextras.Grid (Function reference: 3.1) \| \| uiextras.GridFlex \| uiextras.GridFlex (Function reference: 3.2) \| \| uiextras.HBox \| uiextras.HBox (Function reference: 2.1) \| \| uiextras.HBoxFlex \| uiextras.HBoxFlex (Function reference: 2.3) \| \| uiextras.HButtonBox \| uiextras.HButtonBox (Function reference: 2.5) \| \| uiextras.Panel \| uiextras.Panel (Function reference: 1.1) \| \| uiextras.set \| uiextras.set (Function reference: 4.4) \| \| uiextras.TabPanel \| uiextras.TabPanel (Function reference: 1.4) \| \| uiextras.VBox \| uiextras.VBox (Function reference: 2.2) \| \| uiextras.VBoxFlex \| uiextras.VBoxFlex (Function reference: 2.4) \| \| uiextras.VButtonBox \| uiextras.VButtonBox (Function reference: 2.6) \| \| Understanding layouts \| Understanding layouts (User guide: 1) \| \| Undock \| Dock and undock (User guide: 5.3) \| \| Uninstall \| Installation (Getting Started: 3) \| \| updateInterface \| updateInterface (User guide: 8.3) \| \| User guide \| (User guide) \| \| Using layouts inside GUIDE GUIs \| Using layouts inside GUIDE GUIs (User guide: 6) \| \| Using property defaults \| Using property defaults (User guide: 4) \| \| ---   V \| \| \| VBox \| uiextras.VBox (Function reference: 2.2) \| \| VBoxFlex \| uiextras.VBoxFlex (Function reference: 2.4) \| \| VButtonBox \| uiextras.VButtonBox (Function reference: 2.6) \| \| Version \| layoutVersion (Function reference: 4.2) \| \| Vertical button layout \| uiextras.VButtonBox (Function reference: 2.6) \| \| Vertical layout \| uiextras.VBox (Function reference: 2.2) \| \|  \| uiextras.VBoxFlex (Function reference: 2.4) \| \| Visible example \| Visible example (User guide: 3.2) \| \| ---   W \| \| \| What is the GUI Layout Toolbox? \| What is the GUI Layout Toolbox? (Getting Started: 1) \| \| Why use layouts? \| Why use layouts? (User guide: 1.5) \| \|  \| \| \|  \| \| \|  \| \| |

  

Back to Top

© 2013 The MathWorks Inc
• Terms of Use
•  Patents
•  Trademarks
