# Supplementary material for: Rapid Global Fitting of Large Fluorescence Lifetime Imaging Microscopy Datasets
Source: PLoS One. 2013 Aug 5;8(8):e70687. doi: 10.1371/journal.pone.0070687 (PMC3734241; doi:10.1371/journal.pone.0070687)
Supplement: File S1 — Compressed archive of the source code for FLIMfit used in the present study. Please note that up to date source code and compiled binaries are available from the Open Microscopy website [54]. (ZIP) [file pone.0070687.s001.zip › FLIMFit 4_3_3 Source/GlobalProcessingFrontEnd/HelperFunctions/GUILayout/layoutHelp/Examples.html]

Examples


|  |  |
| --- | --- |
| **GUI Layout Toolbox 1.12** |  |

# Examples

The following examples are provided as part of this documentation. Click
the name of any example to go to the corresponding page.

#### Basics

Layout heirarchy
:   Using layouts inside layouts to produce more complex designs

#### Using axes

Positioning axes
:   Using the different axes position properties.

#### Using panels

Context help
:   Adding context-sensitive help using panels.

Minimize and maximize
:   Creating a user interface with panels that can be minimized and
    maximized.

Dock and undock
:   Creating a user interface with panels that can be undocked into
    separate windows.

#### Enable and Visible

Enabling/Disabling a layout
:   Using the "Enable" property to enable or disable groups of buttons

Showing/Hiding a layout
:   Using the "Visible" property to show or hide groups of buttons

#### Applications

Building a complete application
:   Using layouts together to produce a complete application

  
  

|  |  |  |  |  |
| --- | --- | --- | --- | --- |
|  | Front page | [Top] | Front page |  |

© 2013 The MathWorks Ltd
• Terms of Use
• Patents
• Trademarks
