# Supplementary material for: Rapid Global Fitting of Large Fluorescence Lifetime Imaging Microscopy Datasets
Source: PLoS One. 2013 Aug 5;8(8):e70687. doi: 10.1371/journal.pone.0070687 (PMC3734241; doi:10.1371/journal.pone.0070687)
Supplement: File S1 — Compressed archive of the source code for FLIMfit used in the present study. Please note that up to date source code and compiled binaries are available from the Open Microscopy website [54]. (ZIP) [file pone.0070687.s001.zip › FLIMFit 4_3_3 Source/GlobalProcessingFrontEnd/HelperFunctions/GUILayout/layoutHelp/Function reference.html]

Function reference


|  |  |
| --- | --- |
| **GUI Layout Toolbox 1.12** |  |

# Function reference


|  |  |
| --- | --- |
| 1: Panels | |
| 1.1 uiextras.Panel | Arrange a single element inside a standard panel |
| 1.2 uiextras.CardPanel | Show one element (card) from a list |
| 1.3 uiextras.BoxPanel | Arrange a single element in a panel with boxed title |
| 1.4 uiextras.TabPanel | Arrange elements in a panel with tabs for selecting which is visible |
| 2: Boxes | |
| 2.1 uiextras.HBox | Arrange elements horizontally in a single row |
| 2.2 uiextras.VBox | Arrange elements vertically in a single column |
| 2.3 uiextras.HBoxFlex | Arrange elements horizontally with draggable dividers |
| 2.4 uiextras.VBoxFlex | Arrange elements vertically with draggable dividers |
| 2.5 uiextras.HButtonBox | Arrange buttons horizontally in a single row |
| 2.6 uiextras.VButtonBox | Arrange buttons vertically in a single column |
| 3: Grids | |
| 3.1 uiextras.Grid | Arrange elements in a two dimensional grid |
| 3.2 uiextras.GridFlex | Arrange elements in a two dimensional grid with draggable dividers |
| 4: Other functions | |
| 4.1 uiextras.Empty | Create an empty space |
| 4.2 layoutVersion | Get the toolbox version number and date |
| 4.3 layoutRoot | Return the folder containing the GUI layout toolbox |
| 4.4 uiextras.set | Store a default property value in a parent object |
| 4.5 uiextras.get | Retrieve a default property value from a parent object |

  
  

|  |  |  |  |  |
| --- | --- | --- | --- | --- |
|  | Front page | [Top] | uiextras.Panel |  |

© 2013 The MathWorks Ltd
• Terms of Use
• Patents
• Trademarks
