# Supplementary material for: Rapid Global Fitting of Large Fluorescence Lifetime Imaging Microscopy Datasets
Source: PLoS One. 2013 Aug 5;8(8):e70687. doi: 10.1371/journal.pone.0070687 (PMC3734241; doi:10.1371/journal.pone.0070687)
Supplement: File S1 — Compressed archive of the source code for FLIMfit used in the present study. Please note that up to date source code and compiled binaries are available from the Open Microscopy website [54]. (ZIP) [file pone.0070687.s001.zip › FLIMFit 4_3_3 Source/GlobalProcessingFrontEnd/HelperFunctions/GUILayout/layoutHelp/Function reference1_1.html]

uiextras.Panel


|  |  |
| --- | --- |
| **Function reference** |  |

  

## 1.1: uiextras.Panel

  

Arrange a single element inside a standard panel

`obj` `= uiextras.Panel(` `)`
:   creates a standard `uipanel` object but with automatic management
    of the contained widget or layout. The properties available are largely
    the same as the builtin `uipanel` object. Where more than one child is
    added, the currently visible child is determined using the SelectedChild property.

`obj` `= uiextras.Panel(` `prop``,` `value``,` `...` `)`
:   also sets one or more property values.

### uiextras.Panel properties

| Property | Value | Description |
| --- | --- | --- |
| `BackgroundColor` | `colorspec` | The color to use for exposed areas of the layout background. This can be an RGB triple (e.g. [0 0 1]) or a colour name (e.g. 'b'). |
| `BeingDeleted` | [ on \| off ] | is the object in the process of being deleted. |
| `BorderType` | [ none \| etchedin \| etchedout \| beveledin \| beveledout \| line ] | Type of border around the uipanel area. |
| `BorderWidth` | positive integer | Width of the panel border. |
| `Children` | handle array | List of child objects within this layout (note that this can only be set to permutations of itself). |
| `DeleteFcn` | `function_handle` | Function to call when the layout is being deleted |
| `Enable` | [ on \| off ] | Is interaction with this layout enabled? Note that changing the enable property will cause all children contained to also be enabled/disabled, however since they can be individually re-enabled the state of this property does not reflect the state of the children. See the enable example for more details. |
| `FontAngle` | [ normal \| italic \| oblique ] | Title font angle. |
| `FontName` | string | Title font name (e.g. Arial, Helvetica etc). |
| `FontSize` | positive integer | Title font size. |
| `FontUnits` | [ inches \| centimeters \| normalized \| points \| pixels ] | Title font units for measuring size. |
| `FontWeight` | [ light \| normal \| demi \| bold ] | Title font weight. |
| `ForegroundColor` | `colorspec` | Title font color and/or color of 2-D border line. |
| `HighlightColor` | `colorspec` | 3-D frame highlight color. |
| `Padding` | positive integer | Number of pixels of extra space around the outside of the layout. |
| `Parent` | handle | The handle of the parent figure or container. |
| `Position` | [x y w h] | The position (x,y) and size (w,h) of the layout. |
| `SelectedChild` | positive integer or empty | Which child is visible. |
| `ShadowColor` | `colorspec` | 3-D frame shadow color. |
| `Tag` | string | A string that can be used to identify this object later. |
| `Title` | string | Title string. |
| `TitlePosition` | [ lefttop \| centertop \| righttop \| leftbottom \| centerbottom \| rightbottom ] | Location of title string in relation to the panel. |
| `Type` | string | the object type (class). |
| `Units` | [ inches \| centimeters \| normalized \| points \| pixels \| characters ] | The units of measurement for position the layout within its parent. |
| `Visible` | [ on \| off ] | Is the object visible on screen. |

### For example:

```
f = figure();
p = uiextras.Panel( 'Parent', f, 'Title', 'A Panel', 'Padding', 5 );
uicontrol( 'Parent', p, 'Background', 'r' )
```

```
f = figure();
p = uiextras.Panel( 'Parent', f, 'Title', 'A Panel', 'TitlePosition', 'CenterTop');
b = uiextras.HBox( 'Parent', p, 'Spacing', 5, 'Padding', 5  );
uicontrol( 'Style', 'listbox', 'Parent', b, 'String', {'Item 1','Item 2'} );
uicontrol( 'Parent', b, 'Background', 'b' );
set( b, 'Sizes', [100 -1] );
```

  
**See also:**

- `uiextras.BoxPanel` - for creating a more boxy panel
- `uiextras.TabPanel` - for a panel with tabs for selecting the visible child
- `uiextras.HBox` - for creating a horizontal arrangement within a panel

  

|  |  |  |  |  |
| --- | --- | --- | --- | --- |
|  | Function reference | [Top] | uiextras.CardPanel |  |

© 2013 The MathWorks Ltd
• Terms of Use
• Patents
• Trademarks
