# Supplementary material for: Rapid Global Fitting of Large Fluorescence Lifetime Imaging Microscopy Datasets
Source: PLoS One. 2013 Aug 5;8(8):e70687. doi: 10.1371/journal.pone.0070687 (PMC3734241; doi:10.1371/journal.pone.0070687)
Supplement: File S1 — Compressed archive of the source code for FLIMfit used in the present study. Please note that up to date source code and compiled binaries are available from the Open Microscopy website [54]. (ZIP) [file pone.0070687.s001.zip › FLIMFit 4_3_3 Source/GlobalProcessingFrontEnd/HelperFunctions/GUILayout/layoutHelp/Function reference1_3.html]

uiextras.BoxPanel


|  |  |
| --- | --- |
| **Function reference** |  |

  

## 1.3: uiextras.BoxPanel

  

Arrange a single element in a panel with boxed title

`obj` `= uiextras.BoxPanel(` `)`
:   creates a box-styled panel object with
    automatic management of the contained widget or layout. The
    properties available are largely the same as the builtin `UIPANEL`
    object. Where more than one child is added, the currently visible
    child is determined using the SelectedChild property.

`obj` `= uiextras.BoxPanel(` `prop``,` `value``,` `...` `)`
:   also sets one or more property values.

### uiextras.BoxPanel properties

| Property | Value | Description |
| --- | --- | --- |
| `BackgroundColor` | `colorspec` | The color to use for exposed areas of the layout background. This can be an RGB triple (e.g. [0 0 1]) or a colour name (e.g. 'b'). |
| `BeingDeleted` | [ on \| off ] | is the object in the process of being deleted. |
| `Children` | handle array | List of child objects within this layout (note that this can only be set to permutations of itself). |
| `CloseRequestFcn` | `function_handle` | Function to call when the panel close icon is clicked. Note that if empty, no close button is shown. |
| `DeleteFcn` | `function_handle` | Function to call when the layout is being deleted |
| `DockFcn` | function handle | Function to call when panel is docked or undocked. Note that if empty, no dock button is shown. See advanced manoeuvres with panels for details. |
| `Enable` | [ on \| off ] | Is interaction with this layout enabled? Note that changing the enable property will cause all children contained to also be enabled/disabled, however since they can be individually re-enabled the state of this property does not reflect the state of the children. See the enable example for more details. |
| `FontAngle` | [ normal \| italic \| oblique ] | Title font angle. |
| `FontName` | string | Title font name (e.g. Arial, Helvetica etc). |
| `FontSize` | positive integer | Title font size. |
| `FontUnits` | [ inches \| centimeters \| normalized \| points \| pixels ] | Title font units for measuring size. |
| `FontWeight` | [ light \| normal \| demi \| bold ] | Title font weight. |
| `ForegroundColor` | `colorspec` | Title font color and/or color of 2-D border line. |
| `HelpFcn` | function handle | Function to call when the help icon is clicked. Note that if empty, no help button is shown. See advanced manoeuvres with panels for details. |
| `HighlightColor` | `colorspec` | 3-D frame highlight color. |
| `IsDocked` | logical | Is this panel in a docked state. See advanced manoeuvres with panels for details. |
| `IsMinimized` | logical | Is this panel in a minimized state. See advanced manoeuvres with panels for details. |
| `MinimizeFcn` | function handle | Function to call when panel is minimized or maximized. Note that if empty, no minimize button is shown. See advanced manoeuvres with panels for details. |
| `Padding` | positive integer | Number of pixels of extra space around the outside of the layout. |
| `Parent` | handle | The handle of the parent figure or container. |
| `Position` | [x y w h] | The position (x,y) and size (w,h) of the layout. |
| `SelectedChild` | positive integer or empty | Which child is visible. |
| `ShadowColor` | `colorspec` | 3-D frame shadow color. |
| `Tag` | string | A string that can be used to identify this object later. |
| `Title` | string | Title string. |
| `TitleColor` | `colorspec` | color for the title bar background. |
| `Type` | string | the object type (class). |
| `Units` | [ inches \| centimeters \| normalized \| points \| pixels \| characters ] | The units of measurement for position the layout within its parent. |
| `Visible` | [ on \| off ] | Is the object visible on screen. See the visible example for more details. |

### For example:

```
f = figure();
p = uiextras.BoxPanel( 'Parent', f, 'Title', 'A BoxPanel', 'Padding', 5 );
uicontrol( 'Parent', p, 'Background', 'r' )
```

```
f = figure();
p = uiextras.BoxPanel( 'Parent', f, 'Title', 'A BoxPanel', 'Padding', 5 );
b = uiextras.HBox( 'Parent', p, 'Spacing', 5, 'Padding', 5  );
uicontrol( 'Style', 'listbox', 'Parent', b, 'String', {'Item 1','Item 2'} );
uicontrol( 'Parent', b, 'Background', 'b' );
set( b, 'Sizes', [100 -1] );
p.FontSize = 12;
p.FontWeight = 'bold';
p.HelpFcn = @(x,y) disp('Help me!');
```

  
**See also:**

- `uiextras.Panel` - for creating a more standard panel
- `uiextras.TabPanel` - for a panel with tabs for selecting the visible child
- `uiextras.HBox` - for creating a horizontal arrangement within a panel

  

|  |  |  |  |  |
| --- | --- | --- | --- | --- |
|  | uiextras.CardPanel | [Top] | uiextras.TabPanel |  |

© 2013 The MathWorks Ltd
• Terms of Use
• Patents
• Trademarks
