# Supplementary material for: Rapid Global Fitting of Large Fluorescence Lifetime Imaging Microscopy Datasets
Source: PLoS One. 2013 Aug 5;8(8):e70687. doi: 10.1371/journal.pone.0070687 (PMC3734241; doi:10.1371/journal.pone.0070687)
Supplement: File S1 — Compressed archive of the source code for FLIMfit used in the present study. Please note that up to date source code and compiled binaries are available from the Open Microscopy website [54]. (ZIP) [file pone.0070687.s001.zip › FLIMFit 4_3_3 Source/GlobalProcessingFrontEnd/HelperFunctions/GUILayout/layoutHelp/Function reference1_4.html]

uiextras.TabPanel


|  |  |
| --- | --- |
| **Function reference** |  |

  

## 1.4: uiextras.TabPanel

  

Arrange elements in a panel with tabs for selecting which is visible

`obj` `= uiextras.TabPanel(` `)`
:   creates a panel with tabs along one edge
    to allow selection between the different child objects contained.

`obj` `= uiextras.TabPanel(` `prop``,` `value``,` `...` `)`
:   also sets one or more property values.

### uiextras.TabPanel properties

| Property | Value | Description |
| --- | --- | --- |
| `BackgroundColor` | `colorspec` | The color to use for exposed areas of the layout background. This can be an RGB triple (e.g. [0 0 1]) or a colour name (e.g. 'b'). |
| `BeingDeleted` | [ on \| off ] | is the object in the process of being deleted. |
| `Callback` | `function_handle` | Function to call when the selected tab is changed. The event-data supplied has fields `PreviousChild` and `SelectedChild` giving the previously selected and newly selected tab indices. |
| `Children` | handle array | List of child objects within this layout (note that this can only be set to permutations of itself). |
| `DeleteFcn` | `function_handle` | Function to call when the layout is being deleted |
| `Enable` | [ on \| off ] | Is interaction with this layout enabled? Note that changing the enable property will cause all children contained to also be enabled/disabled, however since they can be individually re-enabled the state of this property does not reflect the state of the children. See the enable example for more details. |
| `FontAngle` | [ normal \| italic \| oblique ] | Title font angle. |
| `FontName` | string | Title font name (e.g. Arial, Helvetica etc). |
| `FontSize` | positive integer | Title font size. |
| `FontUnits` | [ inches \| centimeters \| normalized \| points \| pixels ] | Title font units for measuring size. |
| `FontWeight` | [ light \| normal \| demi \| bold ] | Title font weight. |
| `ForegroundColor` | `colorspec` | Title font color and/or color of 2-D border line. |
| `HighlightColor` | `colorspec` | 3-D frame highlight color. |
| `ShadowColor` | `colorspec` | 3-D frame shadow color. |
| `IsMinimized` | logical | Is this panel in a minimized state. |
| `MinimizeFcn` | function handle | Function to call when panel is minimized or maximized. |
| `Padding` | positive integer | Number of pixels of extra space around the outside of the layout. |
| `Parent` | handle | The handle of the parent figure or container. |
| `Position` | [x y w h] | The position (x,y) and size (w,h) of the layout. |
| `SelectedChild` | positive integer or empty | Which child is visible. |
| `TabSize` | positive integer | Size (width) of the tabs in pixels (default 50). |
| `TabEnable` | cell array of [ on \| off ] | A list of the enabled state of each tab (default is all 'on'). |
| `TabNames` | cell array of strings | A list of the names of the tabs with one entry per tab. |
| `Tag` | string | A string that can be used to identify this object later. |
| `Type` | string | the object type (class). |
| `Units` | [ inches \| centimeters \| normalized \| points \| pixels \| characters ] | The units of measurement for position the layout within its parent. |
| `Visible` | [ on \| off ] | Is the object visible on screen. |

### For example:

```
f = figure();
p = uiextras.TabPanel( 'Parent', f, 'Padding', 5 );
uicontrol( 'Parent', p, 'Background', 'r' );
uicontrol( 'Parent', p, 'Background', 'b' );
uicontrol( 'Parent', p, 'Background', 'g' );
p.TabNames = {'Red', 'Blue', 'Green'};
p.SelectedChild = 2;
```

  
**See also:**

- `uiextras.Panel` - for creating a more standard panel
- `uiextras.BoxPanel` - for creating a panel with boxed title

  

|  |  |  |  |  |
| --- | --- | --- | --- | --- |
|  | uiextras.BoxPanel | [Top] | uiextras.HBox |  |

© 2013 The MathWorks Ltd
• Terms of Use
• Patents
• Trademarks
