# Supplementary material for: Rapid Global Fitting of Large Fluorescence Lifetime Imaging Microscopy Datasets
Source: PLoS One. 2013 Aug 5;8(8):e70687. doi: 10.1371/journal.pone.0070687 (PMC3734241; doi:10.1371/journal.pone.0070687)
Supplement: File S1 — Compressed archive of the source code for FLIMfit used in the present study. Please note that up to date source code and compiled binaries are available from the Open Microscopy website [54]. (ZIP) [file pone.0070687.s001.zip › FLIMFit 4_3_3 Source/GlobalProcessingFrontEnd/HelperFunctions/GUILayout/layoutHelp/Function reference2_4.html]

uiextras.VBoxFlex


|  |  |
| --- | --- |
| **Function reference** |  |

  

## 2.4: uiextras.VBoxFlex

  

Arrange elements vertically with draggable dividers

`obj` `= uiextras.VBoxFlex(` `)`
:   creates a new vertical box layout with draggable dividers and with all properties set to defaults.
    The output is a new layout object that can be used as the parent for other user-interface components.

`obj` `= uiextras.VBoxFlex(` `prop``,` `value``,` `...` `)`
:   also sets one or more property values from the list below.

### uiextras.VBoxFlex properties

| Property | Value | Description |
| --- | --- | --- |
| `BackgroundColor` | `colorspec` | The color to use for exposed areas of the layout background. This can be an RGB triple (e.g. [0 0 1]) or a colour name (e.g. 'b'). |
| `BeingDeleted` | [ on \| off ] | is the object in the process of being deleted. |
| `Children` | handle array | List of child objects within this layout (note that this can only be set to permutations of itself). |
| `DeleteFcn` | `function_handle` | Function to call when the layout is being deleted |
| `Enable` | [ on \| off ] | Is interaction with this layout enabled? Note that changing the enable property will cause all children contained to also be enabled/disabled, however since they can be individually re-enabled the state of this property does not reflect the state of the children. See the enable example for more details. |
| `MinimumSizes` | double vector | The minimum size in pixels for each of the child elements. The size of this vector must always match the number of elements (children). |
| `Padding` | positive integer | Number of pixels of extra space around the outside of the layout. |
| `Parent` | handle | The handle of the parent figure or container. |
| `Position` | [x y w h] | The position (x,y) and size (w,h) of the layout. |
| `ShowMarkings` | [ on \| off ] | Show markings on the draggable dividers (default 'on'). |
| `Sizes` | double vector | The size of each of the child elements. Positive entries indicate fixed sizes in pixels, negative values indicate relative weights for resizing. The size of this vector must always match the number of elements (children). |
| `Spacing` | positive integer | Number of pixels of extra space to leave between elements in the layout. |
| `Tag` | string | A string that can be used to identify this object later. |
| `Type` | string | the object type (class). |
| `Units` | [ inches \| centimeters \| normalized \| points \| pixels \| characters ] | The units of measurement for position the layout within its parent. |
| `Visible` | [ on \| off ] | Is the object visible on screen. |

### For example:

```
f = figure( 'Name', 'uiextras.VBoxFlex example' );
b = uiextras.VBoxFlex( 'Parent', f );
uicontrol( 'Parent', b, 'Background', 'r' )
uicontrol( 'Parent', b, 'Background', 'b' )
uicontrol( 'Parent', b, 'Background', 'g' )
uicontrol( 'Parent', b, 'Background', 'y' )
set( b, 'Sizes', [-1 100 -2 -1], 'Spacing', 5 );
```

  
**See also:**

- `uiextras.VBox` - for creating a vertical arrangement
- `uiextras.HBoxFlex` - for creating a horizontal arrangement with draggable dividers

  

|  |  |  |  |  |
| --- | --- | --- | --- | --- |
|  | uiextras.HBoxFlex | [Top] | uiextras.HButtonBox |  |

© 2013 The MathWorks Ltd
• Terms of Use
• Patents
• Trademarks
