# Supplementary material for: Rapid Global Fitting of Large Fluorescence Lifetime Imaging Microscopy Datasets
Source: PLoS One. 2013 Aug 5;8(8):e70687. doi: 10.1371/journal.pone.0070687 (PMC3734241; doi:10.1371/journal.pone.0070687)
Supplement: File S1 — Compressed archive of the source code for FLIMfit used in the present study. Please note that up to date source code and compiled binaries are available from the Open Microscopy website [54]. (ZIP) [file pone.0070687.s001.zip › FLIMFit 4_3_3 Source/GlobalProcessingFrontEnd/HelperFunctions/GUILayout/layoutHelp/Function reference2_6.html]

uiextras.VButtonBox


|  |  |
| --- | --- |
| **Function reference** |  |

  

## 2.6: uiextras.VButtonBox

  

Arrange buttons vertically in a single column

`obj` `= uiextras.VButtonBox(` `)`
:   is a type of VBox specialised for
    arranging a column of buttons, check-boxes or similar graphical
    elements. All buttons are given equal size and by default are
    centered in the drawing area. The justification can be changed as
    required.

`obj` `= uiextras.VButtonBox(` `prop``,` `value``,` `...` `)`
:   also sets one or more property values.

### uiextras.HBox properties

| Property | Value | Description |
| --- | --- | --- |
| `BackgroundColor` | `colorspec` | The color to use for exposed areas of the layout background. This can be an RGB triple (e.g. [0 0 1]) or a colour name (e.g. 'b'). |
| `BeingDeleted` | [ on \| off ] | is the object in the process of being deleted. |
| `ButtonSize` | [w h] | The size for the buttons (all are given equal size). |
| `Children` | handle array | List of child objects within this layout (note that this can only be set to permutations of itself). |
| `DeleteFcn` | `function_handle` | Function to call when the layout is being deleted |
| `Enable` | [ on \| off ] | Is interaction with this layout enabled? Note that changing the enable property will cause all children contained to also be enabled/disabled, however since they can be individually re-enabled the state of this property does not reflect the state of the children. See the enable example for more details. |
| `HorizontalAlignment` | [ left \| center \| right ] | The horizontal position of the buttons. |
| `Padding` | positive integer | Number of pixels of extra space around the outside of the layout. |
| `Parent` | handle | The handle of the parent figure or container. |
| `Position` | [x y w h] | The position (x,y) and size (w,h) of the layout. |
| `Spacing` | positive integer | Number of pixels of extra space to leave between elements in the layout. |
| `Tag` | string | A string that can be used to identify this object later. |
| `Type` | string | the object type (class). |
| `Units` | [ inches \| centimeters \| normalized \| points \| pixels \| characters ] | The units of measurement for position the layout within its parent. |
| `VerticalAlignment` | [ top \| middle \| bottom ] | The vertical position of the buttons. |
| `Visible` | [ on \| off ] | Is the object visible on screen. |

### For example:

```
f = figure();
b = uiextras.VButtonBox( 'Parent', f );
uicontrol( 'Parent', b, 'String', 'One' );
uicontrol( 'Parent', b, 'String', 'Two' );
uicontrol( 'Parent', b, 'String', 'Three' );
set( b, 'ButtonSize', [130 35], 'Spacing', 5 );
```

  
**See also:**

- `uiextras.HButtonBox` - for creating a horizontal arrangement of buttons
- `uiextras.VBox` - for creating a general vertical arrangement

  

|  |  |  |  |  |
| --- | --- | --- | --- | --- |
|  | uiextras.HButtonBox | [Top] | uiextras.Grid |  |

© 2013 The MathWorks Ltd
• Terms of Use
• Patents
• Trademarks
