# Supplementary material for: Rapid Global Fitting of Large Fluorescence Lifetime Imaging Microscopy Datasets
Source: PLoS One. 2013 Aug 5;8(8):e70687. doi: 10.1371/journal.pone.0070687 (PMC3734241; doi:10.1371/journal.pone.0070687)
Supplement: File S1 — Compressed archive of the source code for FLIMfit used in the present study. Please note that up to date source code and compiled binaries are available from the Open Microscopy website [54]. (ZIP) [file pone.0070687.s001.zip › FLIMFit 4_3_3 Source/GlobalProcessingFrontEnd/HelperFunctions/GUILayout/layoutHelp/Function reference4_1.html]

uiextras.Empty


|  |  |
| --- | --- |
| **Function reference** |  |

  

## 4.1: uiextras.Empty

Create an empty space

`obj` `= uiextras.Empty(` `)`
:   creates an empty space object that can be
    used in layouts to add gaps between other elements.

`obj` `= uiextras.Empty(` `param``,` `value``,` `...` `)`
:   also sets one or more property values.

### uiextras.Empty properties

| Property | Value | Description |
| --- | --- | --- |
| `BeingDeleted` | [ on \| off ] | is the object in the process of being deleted. |
| `Parent` | handle | The handle of the parent figure or container. |
| `Position` | [x y w h] | The position (x,y) and size (w,h) of the layout. |
| `Tag` | string | A string that can be used to identify this object later. |
| `Type` | string | the object type (class). |
| `Units` | [ inches \| centimeters \| normalized \| points \| pixels \| characters ] | The units of measurement for position the layout within its parent. |
| `Visible` | [ on \| off ] | Is the object visible on screen. |

### For example:

```
f = figure();
box = uiextras.HBox( 'Parent', f, 'Spacing', 5 );
uicontrol( 'Parent', box, 'Background', 'r' )
uiextras.Empty( 'Parent', box )
uicontrol( 'Parent', box, 'Background', 'g' )
```

  
**See also:**

- `uiextras.HBox` - for arranging widgets horizontally

  

|  |  |  |  |  |
| --- | --- | --- | --- | --- |
|  | uiextras.GridFlex | [Top] | layoutVersion |  |

© 2013 The MathWorks Ltd
• Terms of Use
• Patents
• Trademarks
