# Supplementary material for: Rapid Global Fitting of Large Fluorescence Lifetime Imaging Microscopy Datasets
Source: PLoS One. 2013 Aug 5;8(8):e70687. doi: 10.1371/journal.pone.0070687 (PMC3734241; doi:10.1371/journal.pone.0070687)
Supplement: File S1 — Compressed archive of the source code for FLIMfit used in the present study. Please note that up to date source code and compiled binaries are available from the Open Microscopy website [54]. (ZIP) [file pone.0070687.s001.zip › FLIMFit 4_3_3 Source/GlobalProcessingFrontEnd/HelperFunctions/GUILayout/layoutHelp/Function reference4_2.html]

layoutVersion


|  |  |
| --- | --- |
| **Function reference** |  |

  

## 4.2: layoutVersion

Get the toolbox version number and date

`V` `= layoutVersion(` `)`
:   returns the version number as a string.

`[``V``,` `D``] = layoutVersion(` `)`
:   returns both the version string and the date of creation (format is ISO8601, i.e. YYYY-MM-DD)

### For example:

```
[v,d] = layoutVersion()
```

`v = '1.0'  
d = '2010-05-28'`

  
**See also:**

- `layoutRoot` - returns the folder containing the GUI layout toolbox

  

|  |  |  |  |  |
| --- | --- | --- | --- | --- |
|  | uiextras.Empty | [Top] | layoutRoot |  |

© 2013 The MathWorks Ltd
• Terms of Use
• Patents
• Trademarks
