# Supplementary material for: Rapid Global Fitting of Large Fluorescence Lifetime Imaging Microscopy Datasets
Source: PLoS One. 2013 Aug 5;8(8):e70687. doi: 10.1371/journal.pone.0070687 (PMC3734241; doi:10.1371/journal.pone.0070687)
Supplement: File S1 — Compressed archive of the source code for FLIMfit used in the present study. Please note that up to date source code and compiled binaries are available from the Open Microscopy website [54]. (ZIP) [file pone.0070687.s001.zip › FLIMFit 4_3_3 Source/GlobalProcessingFrontEnd/HelperFunctions/GUILayout/layoutHelp/Function reference4_3.html]

layoutRoot


|  |  |
| --- | --- |
| **Function reference** |  |

  

## 4.3: layoutRoot

returns the folder containing the GUI layout toolbox

`folder` `= layoutRoot(` `)`
:   returns the full path to the folder containing the GUI layout toolbox.

### For example:

```
folder = layoutRoot()
```

`folder = 'C:\Temp\LayoutToolbox1.0'`

  
**See also:**

- `layoutVersion` - for getting the toolbox version number and date

  

|  |  |  |  |  |
| --- | --- | --- | --- | --- |
|  | layoutVersion | [Top] | uiextras.set |  |

© 2013 The MathWorks Ltd
• Terms of Use
• Patents
• Trademarks
