# Supplementary material for: Rapid Global Fitting of Large Fluorescence Lifetime Imaging Microscopy Datasets
Source: PLoS One. 2013 Aug 5;8(8):e70687. doi: 10.1371/journal.pone.0070687 (PMC3734241; doi:10.1371/journal.pone.0070687)
Supplement: File S1 — Compressed archive of the source code for FLIMfit used in the present study. Please note that up to date source code and compiled binaries are available from the Open Microscopy website [54]. (ZIP) [file pone.0070687.s001.zip › FLIMFit 4_3_3 Source/GlobalProcessingFrontEnd/HelperFunctions/GUILayout/layoutHelp/Function reference4_4.html]

uiextras.set


|  |  |
| --- | --- |
| **Function reference** |  |

  

## 4.4: uiextras.set

Store a default property value in a parent object

`uiextras.set(` `hObj``,` `propName``,` `propValue` `)`
:   stores a default property
    value in the object hObj such that children created inside it of the
    correct class will use the specified value (`propValue`) for
    the specified property by default. The property name should take the
    form "DefaultClassProperty", for example to set the default "TitleColor"
    for the class "uiextras.BoxPanel", use "DefaultBoxPanelTitleColor".
    Note that using the special object handle 0 sets the default for all
    new figures.

### For example:

```
uiextras.set( gcf(), 'DefaultBoxPanelTitleColor', 'g' )
```

```
uiextras.set( 0, 'DefaultHBoxPadding', 5 )
```

  
**See also:**

- `uiextras.get` - for retrieving a default property value from a parent object

  

|  |  |  |  |  |
| --- | --- | --- | --- | --- |
|  | layoutRoot | [Top] | uiextras.get |  |

© 2013 The MathWorks Ltd
• Terms of Use
• Patents
• Trademarks
