# Supplementary material for: Rapid Global Fitting of Large Fluorescence Lifetime Imaging Microscopy Datasets
Source: PLoS One. 2013 Aug 5;8(8):e70687. doi: 10.1371/journal.pone.0070687 (PMC3734241; doi:10.1371/journal.pone.0070687)
Supplement: File S1 — Compressed archive of the source code for FLIMfit used in the present study. Please note that up to date source code and compiled binaries are available from the Open Microscopy website [54]. (ZIP) [file pone.0070687.s001.zip › FLIMFit 4_3_3 Source/GlobalProcessingFrontEnd/HelperFunctions/GUILayout/layoutHelp/Function reference4_5.html]

uiextras.get


|  |  |
| --- | --- |
| **Function reference** |  |

  

## 4.5: uiextras.get

Retrieve a default property value from a parent object

`propValue` `= uiextras.get(` `hObj``,` `propName` `)`
:   retrieves a default property
    value from the object hObj. Note that the hObj must be a valid graphics
    container object such as a uipanel, figure or the special flag 0 (the
    overall environment). The property name should take the form
    "DefaultClassProperty", for example to get the default "TitleColor" for
    the class "uiextras.BoxPanel", use "DefaultBoxPanelTitleColor".If no
    default has been set for the specified property an error is thrown.

### For example:

```
p = uiextras.get( gcf(), 'DefaultBoxPanelTitleColor' )
```

```
p = uiextras.get( 0, 'DefaultHBoxPadding' )
```

  
**See also:**

- `uiextras.set` - for setting a default property value in a parent object

  

|  |  |  |  |  |
| --- | --- | --- | --- | --- |
|  | uiextras.set | [Top] | Front page |  |

© 2013 The MathWorks Ltd
• Terms of Use
• Patents
• Trademarks
>
