# Supplementary material for: Rapid Global Fitting of Large Fluorescence Lifetime Imaging Microscopy Datasets
Source: PLoS One. 2013 Aug 5;8(8):e70687. doi: 10.1371/journal.pone.0070687 (PMC3734241; doi:10.1371/journal.pone.0070687)
Supplement: File S1 — Compressed archive of the source code for FLIMfit used in the present study. Please note that up to date source code and compiled binaries are available from the Open Microscopy website [54]. (ZIP) [file pone.0070687.s001.zip › FLIMFit 4_3_3 Source/GlobalProcessingFrontEnd/HelperFunctions/GUILayout/layoutHelp/Getting Started.html]

Getting Started


|  |  |
| --- | --- |
| **GUI Layout Toolbox 1.12** |  |

# Getting Started

This chapter gives an overview of this package, including its purpose,
how to install it and any known issues at the time of release.


  


## Contents

- 1. What is the GUI Layout Toolbox?
- 2. Release Notes
- 3. Installation
- 4. Support
- 5. Acknowledgements

  

|  |  |  |  |  |
| --- | --- | --- | --- | --- |
|  | Front page | [Top] | What is the GUI Layout Toolbox? |  |

© 2013 The MathWorks Ltd
• Terms of Use
• Patents
• Trademarks
