# Supplementary material for: Rapid Global Fitting of Large Fluorescence Lifetime Imaging Microscopy Datasets
Source: PLoS One. 2013 Aug 5;8(8):e70687. doi: 10.1371/journal.pone.0070687 (PMC3734241; doi:10.1371/journal.pone.0070687)
Supplement: File S1 — Compressed archive of the source code for FLIMfit used in the present study. Please note that up to date source code and compiled binaries are available from the Open Microscopy website [54]. (ZIP) [file pone.0070687.s001.zip › FLIMFit 4_3_3 Source/GlobalProcessingFrontEnd/HelperFunctions/GUILayout/layoutHelp/Getting Started1.html]

 


What is the GUI Layout Toolbox?


|  |  |
| --- | --- |
| **Getting Started** |  |

## 1: What is the GUI Layout Toolbox?

The GUI Layout Toolbox provides a package of MATLAB objects that allow for complex
arrangement of graphical user interface elements within a figure window. The
main capabilities provided are:

- Automatic element arrangement horizontally, vertically or in grids
- Ability to specify fixed sizes or resizing weights for each element
- Ability to "nest" layouts to produce virtually any element arrangement
- Divider bars for user-resizing of elements

These element arrangement capabilities are designed to match those found as
standard in other user-interface toolkits such as Java Swing, GTK, QT etc.

  

|  |  |  |  |  |
| --- | --- | --- | --- | --- |
|  | Getting Started | [Top] | Release Notes |  |

© 2013 The MathWorks Ltd
• Terms of Use
• Patents
• Trademarks
