# Supplementary material for: Rapid Global Fitting of Large Fluorescence Lifetime Imaging Microscopy Datasets
Source: PLoS One. 2013 Aug 5;8(8):e70687. doi: 10.1371/journal.pone.0070687 (PMC3734241; doi:10.1371/journal.pone.0070687)
Supplement: File S1 — Compressed archive of the source code for FLIMfit used in the present study. Please note that up to date source code and compiled binaries are available from the Open Microscopy website [54]. (ZIP) [file pone.0070687.s001.zip › FLIMFit 4_3_3 Source/GlobalProcessingFrontEnd/HelperFunctions/GUILayout/layoutHelp/Getting Started2.html]

 


Release Notes


|  |  |
| --- | --- |
| **Getting Started** |  |

## 2: Release Notes

The GUI Layout Toolbox version numbers take the form `major.minor`. The
current version you have installed can be checked by typing `ver`
or `layoutVersion` at the MATLAB command prompt.

### Section contents:

- 2.1. Version 1.12 (February 2013)
- 2.2. Version 1.11 (December 2012)
- 2.3. Version 1.10 (July 2011)
- 2.4. Version 1.9 (February 2011)
- 2.5. Version 1.8 (November 2010)
- 2.6. Version 1.7 (October 2010)
- 2.7. Version 1.6 (September 2010)
- 2.8. Version 1.5 (July 2010)
- 2.9. Version 1.4 (July 2010)
- 2.10. Version 1.3 (July 2010)
- 2.11. Version 1.2 (June 2010)
- 2.12. Version 1.1 (June 2010)
- 2.13. Version 1.0 (May 2010)


  

---

## 2.1: Version 1.12 (February 2013)

Update to the first release:

- Fix bug when axes were deleted from layouts in R2013a

  

---

## 2.2: Version 1.11 (December 2012)

Update to the first release:

- Fix bug where axes disappear from tabs
- Fix bug where axes position is corrupted if tabs become too small to draw

  

---

## 2.3: Version 1.10 (July 2011)

Update to the first release:

- Add `TabEnable` property for controlling the enabled state of individual tabs
- Revert to normalized units for all layouts
- Fix bug with mouse-over events

  

---

## 2.4: Version 1.9 (February 2011)

Update to the first release:

- Add `MinimumSize` properties to Boxes and Grids
- Minor improvements to mouse-over and redraw speed
- Fix bug with ordering of controls that sometimes prevented grid dividers being dragged

  

---

## 2.5: Version 1.8 (November 2010)

Update to the first release:

- Add PreviousChild event-data to `uiextras.TabPanel` Callback
- Add guards to prevent errors when focus is lost whilst dragging a flexible divider
- Fix bug when trying to print figures containing flexible layouts

  

---

## 2.6: Version 1.7 (October 2010)

Update to the first release:

- Improve enable behavior when used with custom widgets
- Add an explanation of cell arrays used to pass extra function arguments
- Fix bug in unit-setting of children added to layouts (improves axes handling)

  

---

## 2.7: Version 1.6 (September 2010)

Update to the first release:

- Make panel "SelectedChild" property observable for use with `event.proplistener`
- Fix bug with dividers disappearing when GridFlex's are nested
- Fix clipping bug with Tab label text drawing outside parent container

  

---

## 2.8: Version 1.5 (July 2010)

Update to the first release:

- Add DeleteFcn property that allows a user function to be called before a layout is deleted.
- Improve Docking and Minimizing examples.
- Fix flexible layout divider positions when figure units are not set to pixels (e.g. in GUIDE apps).
- Add documentation describing how to use layouts as part of a GUIDE-built GUI.
- Fix a bug where ButtonBoxes caused a new window to open if their parent had HandleVisibility off

  

---

## 2.9: Version 1.4 (July 2010)

Update to the first release:

- Add ability to set defaults for property values. See `uiextras.set`.

  

---

## 2.10: Version 1.3 (July 2010)

Update to the first release:

- Fix bug with `uiextras.Panel` mis-managing its title when not specified at construction
- Fix bug with `uiextras.Empty` widget going out of scope
- Fix bug when removing a child from `uiextras.TabPanel`
- Prevent legends being hijacked by the layout managers!
- Add option for showing markings on the movable dividers for flexible containers (`uiextras.HBoxFlex`,`uiextras.VBoxFlex`,`uiextras.GridFlex`)
- Add documentation on building deployed exectuables
- Tidy up help H1 lines in all files

  

---

## 2.11: Version 1.2 (June 2010)

Update to the first release:

- Ensure layout objects are deleted when the graphics are deleted
- Add "ancestor" method
- Fix bug in GridFlex divider positioning
- Remove ">>" from examples in documentation
- Improve formatting of examples in documentation

  

---

## 2.12: Version 1.1 (June 2010)

Update to the first release:

- Added missing `layoutRoot` and `layoutVersion` functions.
- Fix various typographical errors in the documentation.

  

---

## 2.13: Version 1.0 (May 2010)

Initial public release. Includes:

1. Panels
   - `uiextras.Panel`: Arrange a single element inside a standard panel
   - `uiextras.CardPanel`: Show one element (card) from a list
   - `uiextras.BoxPanel`: Arrange a single element in a panel with boxed title
   - `uiextras.TabPanel`: Arrange elements in a panel with tabs for selecting which is visible
2. Boxes
   - `uiextras.HBox`: Arrange elements horizontally in a single row
   - `uiextras.VBox`: Arrange elements vertically in a single column
   - `uiextras.HBoxFlex`: Arrange elements horizontally with draggable dividers
   - `uiextras.VBoxFlex`: Arrange elements vertically with draggable dividers
   - `uiextras.HButtonBox`: Arrange buttons horizontally in a single row
   - `uiextras.VButtonBox`: Arrange buttons vertically in a single column
3. 3: Grids
   - `uiextras.Grid`: Arrange elements in a two dimensional grid
   - `uiextras.GridFlex`: Arrange elements in a two dimensional grid with draggable dividers

  

|  |  |  |  |  |
| --- | --- | --- | --- | --- |
|  | What is the GUI Layout Toolbox? | [Top] | Installation |  |

© 2013 The MathWorks Ltd
• Terms of Use
• Patents
• Trademarks
