# Supplementary material for: Rapid Global Fitting of Large Fluorescence Lifetime Imaging Microscopy Datasets
Source: PLoS One. 2013 Aug 5;8(8):e70687. doi: 10.1371/journal.pone.0070687 (PMC3734241; doi:10.1371/journal.pone.0070687)
Supplement: File S1 — Compressed archive of the source code for FLIMfit used in the present study. Please note that up to date source code and compiled binaries are available from the Open Microscopy website [54]. (ZIP) [file pone.0070687.s001.zip › FLIMFit 4_3_3 Source/GlobalProcessingFrontEnd/HelperFunctions/GUILayout/layoutHelp/Getting Started3.html]

 


Installation


|  |  |
| --- | --- |
| **Getting Started** |  |

## 3: Installation

To use this toolbox it must be added to the MATLAB path. Scripts are provided
to do this in two ways:

**From MATLAB:**

1. **From MATLAB:** from the folder containing the toolbox, run the "install.m" function
2. **From Windows Explorer:** from the folder containing the toolbox, double-click the "install.bat" icon

To uninstall (i.e. remove it from the path), run the function 'uninstall' in MATLAB, also found in the toolbox folder.

  

|  |  |  |  |  |
| --- | --- | --- | --- | --- |
|  | Release Notes | [Top] | Support |  |

© 2013 The MathWorks Ltd
• Terms of Use
• Patents
• Trademarks
