# Supplementary material for: Rapid Global Fitting of Large Fluorescence Lifetime Imaging Microscopy Datasets
Source: PLoS One. 2013 Aug 5;8(8):e70687. doi: 10.1371/journal.pone.0070687 (PMC3734241; doi:10.1371/journal.pone.0070687)
Supplement: File S1 — Compressed archive of the source code for FLIMfit used in the present study. Please note that up to date source code and compiled binaries are available from the Open Microscopy website [54]. (ZIP) [file pone.0070687.s001.zip › FLIMFit 4_3_3 Source/GlobalProcessingFrontEnd/HelperFunctions/GUILayout/layoutHelp/Getting Started4.html]

 


Support


|  |  |
| --- | --- |
| **Getting Started** |  |

## 4: Support

This toolbox is not a MathWorks supported product. However, if you
have problems, suggestions or other comments, please contact the
authors:

- Ben Tordoff
- David Sampson

If you like this toolbox, help others to find it by leaving a rating
and comment on the MATLAB Central File Exchange.

  

|  |  |  |  |  |
| --- | --- | --- | --- | --- |
|  | Installation | [Top] | Acknowledgements |  |

© 2013 The MathWorks Ltd
• Terms of Use
• Patents
• Trademarks
