# Supplementary material for: Rapid Global Fitting of Large Fluorescence Lifetime Imaging Microscopy Datasets
Source: PLoS One. 2013 Aug 5;8(8):e70687. doi: 10.1371/journal.pone.0070687 (PMC3734241; doi:10.1371/journal.pone.0070687)
Supplement: File S1 — Compressed archive of the source code for FLIMfit used in the present study. Please note that up to date source code and compiled binaries are available from the Open Microscopy website [54]. (ZIP) [file pone.0070687.s001.zip › FLIMFit 4_3_3 Source/GlobalProcessingFrontEnd/HelperFunctions/GUILayout/layoutHelp/Getting Started5.html]

 


Acknowledgements


|  |  |
| --- | --- |
| **Getting Started** |  |

## 5: Acknowledgements

This toolbox has been evolving for several years and is based on the work of many engineering consultants
at MathWorks, including (but not limited to):

- Ben Tordoff
- David Sampson
- Paul Kerr-Delworth
- Malcolm Wood
- Brad Phelan

Many thanks to those who have shared their code and expertise over the years.

  

|  |  |  |  |  |
| --- | --- | --- | --- | --- |
|  | Support | [Top] | Front page |  |

© 2013 The MathWorks Ltd
• Terms of Use
• Patents
• Trademarks
>
