# Supplementary material for: Rapid Global Fitting of Large Fluorescence Lifetime Imaging Microscopy Datasets
Source: PLoS One. 2013 Aug 5;8(8):e70687. doi: 10.1371/journal.pone.0070687 (PMC3734241; doi:10.1371/journal.pone.0070687)
Supplement: File S1 — Compressed archive of the source code for FLIMfit used in the present study. Please note that up to date source code and compiled binaries are available from the Open Microscopy website [54]. (ZIP) [file pone.0070687.s001.zip › FLIMFit 4_3_3 Source/GlobalProcessingFrontEnd/HelperFunctions/GUILayout/layoutHelp/helptoc.html]

Table of Contents


 **GUI Layout**  
  
  **Getting Started**  
     1. What is the GUI Layout Toolbox?  
     2. Release Notes  
     3. Installation  
     4. Support  
     5. Acknowledgements  
  
  **Examples**  
  
  **User guide**  
     1. Understanding layouts  
     2. Positioning Axes  
     3. Enable and Visible  
     4. Using property defaults  
     5. Advanced maneuvers with panels  
     6. Using layouts inside GUIDE GUIs  
     7. Deploying GUIs using the MATLAB Compiler  
     8. A complete example  
  
  **Function reference**  
  
  **Index**
