# Supplementary material for: Rapid Global Fitting of Large Fluorescence Lifetime Imaging Microscopy Datasets
Source: PLoS One. 2013 Aug 5;8(8):e70687. doi: 10.1371/journal.pone.0070687 (PMC3734241; doi:10.1371/journal.pone.0070687)
Supplement: File S1 — Compressed archive of the source code for FLIMfit used in the present study. Please note that up to date source code and compiled binaries are available from the Open Microscopy website [54]. (ZIP) [file pone.0070687.s001.zip › FLIMFit 4_3_3 Source/GlobalProcessingFrontEnd/HelperFunctions/GUILayout/layoutHelp/index.html]

GUI Layout Toolbox documentation


# GUI Layout Toolbox documentation

**Version: 1.12**

This toolbox provides tools to create sophisticated MATLAB graphical
user interfaces that resize gracefully. The classes supplied can be
used in combination to produce virtually any user interface layout.

- Arrange MATLAB user-interface components horizontally, vertically or in grids
- Ability to mix fixed size and variable size elements
- Dynamic resizing of elements by dragging dividers
- Use panels and tabs for switching interface pages

This toolbox was developed by Ben Tordoff and David Sampson from the
Consulting group at MathWorks.

## Contents:

|  |  |  |
| --- | --- | --- |
|  | **Getting Started:** | Introductory notes and installation instructions |
|  | **Examples:** | A list of the examples that are provided in the documentation |
|  | **User guide:** | Describes how to use these tools |
|  | **Function reference:** | A list of the available functions |
|  | **Index** | Alphabetical index of sections, functions and concepts |

  

© 2013 The MathWorks Ltd
• Terms of Use
• Patents
• Trademarks
