# Supplementary material for: Rapid Global Fitting of Large Fluorescence Lifetime Imaging Microscopy Datasets
Source: PLoS One. 2013 Aug 5;8(8):e70687. doi: 10.1371/journal.pone.0070687 (PMC3734241; doi:10.1371/journal.pone.0070687)
Supplement: File S1 — Compressed archive of the source code for FLIMfit used in the present study. Please note that up to date source code and compiled binaries are available from the Open Microscopy website [54]. (ZIP) [file pone.0070687.s001.zip › FLIMFit 4_3_3 Source/GlobalProcessingFrontEnd/HelperFunctions/GUILayout/layoutHelp/User guide.html]

User guide


|  |  |
| --- | --- |
| **GUI Layout Toolbox 1.12** |  |

# User guide

This chapter describes the purpose and overall design of the layout
tools that the toolbox provides. It also provides some examples of
creating some user interfaces using layouts.


  


## Contents

- 1. Understanding layouts
- 2. Positioning Axes
- 3. Enable and Visible
- 4. Using property defaults
- 5. Advanced maneuvers with panels
- 6. Using layouts inside GUIDE GUIs
- 7. Deploying GUIs using the MATLAB Compiler
- 8. A complete example

  

|  |  |  |  |  |
| --- | --- | --- | --- | --- |
|  | Front page | [Top] | Understanding layouts |  |

© 2013 The MathWorks Ltd
• Terms of Use
• Patents
• Trademarks
