# Supplementary material for: Rapid Global Fitting of Large Fluorescence Lifetime Imaging Microscopy Datasets
Source: PLoS One. 2013 Aug 5;8(8):e70687. doi: 10.1371/journal.pone.0070687 (PMC3734241; doi:10.1371/journal.pone.0070687)
Supplement: File S1 — Compressed archive of the source code for FLIMfit used in the present study. Please note that up to date source code and compiled binaries are available from the Open Microscopy website [54]. (ZIP) [file pone.0070687.s001.zip › FLIMFit 4_3_3 Source/GlobalProcessingFrontEnd/HelperFunctions/GUILayout/layoutHelp/User guide1.html]

 


Understanding layouts


|  |  |
| --- | --- |
| **User guide** |  |

## 1: Understanding layouts

The purpose of a "layout" is to manage the positioning of one
or more user-interface components. This means that instead of
worrying about the exact position of each user interface component
you instead worry about the relative positioning of them; are they
arranged in a vertical list, horizontal list, a grid pattern, etc.

The best way to understand what layouts are and why they are a good user-interface
design tool is to see some examples. The sections below go through the
basics of building an interface using layouts and how to build complex
interfaces using them.

### Section contents:


- 1.1. Layout basics
- 1.2. Types of layout
- 1.3. Sizes and units
- 1.4. Layout heirarchies
- 1.5. Why use layouts?

  

|  |  |  |  |  |
| --- | --- | --- | --- | --- |
|  | User guide | [Top] | Layout basics |  |

© 2013 The MathWorks Ltd
• Terms of Use
• Patents
• Trademarks
