# Supplementary material for: Rapid Global Fitting of Large Fluorescence Lifetime Imaging Microscopy Datasets
Source: PLoS One. 2013 Aug 5;8(8):e70687. doi: 10.1371/journal.pone.0070687 (PMC3734241; doi:10.1371/journal.pone.0070687)
Supplement: File S1 — Compressed archive of the source code for FLIMfit used in the present study. Please note that up to date source code and compiled binaries are available from the Open Microscopy website [54]. (ZIP) [file pone.0070687.s001.zip › FLIMFit 4_3_3 Source/GlobalProcessingFrontEnd/HelperFunctions/GUILayout/layoutHelp/User guide1_1.html]

Layout basics


|  |  |
| --- | --- |
| **User guide** |  |

  

## 1.1: Layout basics

To see how layouts work, let's use the most basic layout,
a horizontal list (or box). We first create a window:

```
f = figure();
```

Now let's create the horizontal layout and add it to the figure. Note
that in common with other MATLAB graphics objects, one object is added to
another by setting the "Parent" property - this will automatically adjust the list
of "Children" in the parent object. The job of a horizontal box layout is to arrange
its contents in a horizontal line, setting the position of each element to best fill
the space:

```
layout = uiextras.HBox( 'Parent', f );
```

Nothing's changed! That's because the layout is for arranging
other user-interface components - it doesn't draw anything itself. Let's
add some buttons. Note how after creating each button the existing contents
of the box make room for the new addition; we don't need to set the position of
any user-interface component!

```
uicontrol( 'String', 'Button 1', 'Parent', layout );
uicontrol( 'String', 'Button 2', 'Parent', layout );
uicontrol( 'String', 'Button 3', 'Parent', layout );
```

..

Other layouts work in exactly the same way, although visually
the end-result is quite different:

```
f = figure();
layout = uiextras.VBox( 'Parent', f );
uicontrol( 'String', 'Button 1', 'Parent', layout );
uicontrol( 'String', 'Button 2', 'Parent', layout );
uicontrol( 'String', 'Button 3', 'Parent', layout );
```


```
f = figure();
layout = uiextras.TabPanel( 'Parent', f );
uicontrol( 'String', 'Button 1', 'Parent', layout );
uicontrol( 'String', 'Button 2', 'Parent', layout );
uicontrol( 'String', 'Button 3', 'Parent', layout );
```

  

|  |  |  |  |  |
| --- | --- | --- | --- | --- |
|  | Understanding layouts | [Top] | Types of layout |  |

© 2013 The MathWorks Ltd
• Terms of Use
• Patents
• Trademarks
