# Supplementary material for: Rapid Global Fitting of Large Fluorescence Lifetime Imaging Microscopy Datasets
Source: PLoS One. 2013 Aug 5;8(8):e70687. doi: 10.1371/journal.pone.0070687 (PMC3734241; doi:10.1371/journal.pone.0070687)
Supplement: File S1 — Compressed archive of the source code for FLIMfit used in the present study. Please note that up to date source code and compiled binaries are available from the Open Microscopy website [54]. (ZIP) [file pone.0070687.s001.zip › FLIMFit 4_3_3 Source/GlobalProcessingFrontEnd/HelperFunctions/GUILayout/layoutHelp/User guide1_2.html]

Types of layout


|  |  |
| --- | --- |
| **User guide** |  |

  

## 1.2: Types of layout

The layouts in this toolbox come in three forms:

1. **Panels**: show a single child element with some decoration. Other children
   of the layout are hidden from view. The visible child can be switched. Available
   panels include
   Panel,
   CardPanel,
   BoxPanel and
   TabPanel.
2. **Boxes**: arrange children linearly in a single row or column. Available boxes include
   HBox,
   VBox,
   HBoxFlex and
   VBoxFlex.
3. **Grids**: (also known as tables) arrange children in a two-dimensional grid. Available grids include
   Grid and
   GridFlex.

  

|  |  |  |  |  |
| --- | --- | --- | --- | --- |
|  | Layout basics | [Top] | Sizes and units |  |

© 2013 The MathWorks Ltd
• Terms of Use
• Patents
• Trademarks
