# Supplementary material for: Rapid Global Fitting of Large Fluorescence Lifetime Imaging Microscopy Datasets
Source: PLoS One. 2013 Aug 5;8(8):e70687. doi: 10.1371/journal.pone.0070687 (PMC3734241; doi:10.1371/journal.pone.0070687)
Supplement: File S1 — Compressed archive of the source code for FLIMfit used in the present study. Please note that up to date source code and compiled binaries are available from the Open Microscopy website [54]. (ZIP) [file pone.0070687.s001.zip › FLIMFit 4_3_3 Source/GlobalProcessingFrontEnd/HelperFunctions/GUILayout/layoutHelp/User guide1_3.html]

Sizes and units


|  |  |
| --- | --- |
| **User guide** |  |

  

## 1.3: Sizes and units

In the previous section we noted that when using layouts
you never need to set the position or size of a user-interface
component yourself - the layouts take care of it. So how do
you control the sizes of the components?

Each layout that arranges multiple items within its drawing area
has a sizing property: for horizontal and vertical boxes this
is called `Sizes`; for grids we have `RowSizes`
and `ColumnSizes`. These all obey the same convention:

1. Positive numbers indicate sizes in pixels (similar to "pixel" units)
2. Negative numbers indicate a weighting for variable sizing (similar to "normalized" units)

By default all sizes are set to -1 (variable size with unit weighting).

Let's take a simple example:

```
f = figure();
layout = uiextras.HBox( 'Parent', f );
uicontrol( 'String', 'Button 1', 'Parent', layout );
uicontrol( 'String', 'Button 2', 'Parent', layout );
uicontrol( 'String', 'Button 3', 'Parent', layout );
```

We can set the middle element to be twice as wide as the others
(but still variable width) by setting its weight to -2 with the others at -1:

```
layout.Sizes = [-1 -2 -1]
```

Alternatively we might want the first element to have a
fixed width of 100 pixels with the others filling any remaining
space equally:

```
layout.Sizes = [100 -1 -1]
```

This ability to mix fixed and variable sized elements is
crucial in interface design. It really comes into its own when building
a heirarchy of layouts, described next.

  

---

### 1.3.1: Minimum sizes

Many of the multi-element layouts also provide a `MinimumSizes`
property to prevent an element becoming too small. This is measured in
pixels and defaults to one pixel. If the specified minimum sizes cannot fit
within the available space then all element sizes are reduced to fit.

  

|  |  |  |  |  |
| --- | --- | --- | --- | --- |
|  | Types of layout | [Top] | Layout heirarchies |  |

© 2013 The MathWorks Ltd
• Terms of Use
• Patents
• Trademarks
