# Supplementary material for: Rapid Global Fitting of Large Fluorescence Lifetime Imaging Microscopy Datasets
Source: PLoS One. 2013 Aug 5;8(8):e70687. doi: 10.1371/journal.pone.0070687 (PMC3734241; doi:10.1371/journal.pone.0070687)
Supplement: File S1 — Compressed archive of the source code for FLIMfit used in the present study. Please note that up to date source code and compiled binaries are available from the Open Microscopy website [54]. (ZIP) [file pone.0070687.s001.zip › FLIMFit 4_3_3 Source/GlobalProcessingFrontEnd/HelperFunctions/GUILayout/layoutHelp/User guide1_5.html]

Why use layouts?


|  |  |
| --- | --- |
| **User guide** |  |

  

## 1.5: Why use layouts?

MATLAB ships with a GUI design tool called `GUIDE`. This
doesn't use layouts, but forces users to manually position each element. This approach
is a much faster way to build simple user-interfaces, so why would you want to
use layouts?

The over-riding reason for using layouts or layout managers is
to gain control of the resizing behaviour of the interface without
having to write a complex "ResizeFcn". If you simply position user-interface elements
directly (either using `GUIDE` or programmatically), you
have two choices about what happens when the window resizes:

### For example:

**1. The user-interface components scale with the window (normalised units)**  
We didn't really want the buttons to grow but everything resizes in proportion.

```
f = figure( 'Position', 200*ones(1,4) );
axes( 'Parent', f, ...
    'Units', 'Normalized', ...
    'OuterPosition', [0.02 0.2 0.96 0.8] );
uicontrol( 'Parent', f, ...
    'Units', 'Normalized', ...
    'Position', [0.02 0.02 0.46 0.16], ...
    'String', 'Button 1' );
uicontrol( 'Parent', f, ...
    'Units', 'Normalized', ...
    'Position', [0.52 0.02 0.46 0.16], ...
    'String', 'Button 2' );
```

.

**2. The user-interface components stay fixed and the window resize creates empty space (pixel units)**  
Although the buttons don't now grow, neither does the axes, which looks very odd.

```
f = figure( 'Position', 200*ones(1,4) );
axes( 'Parent', f, ...
    'Units', 'Pixels', ...
    'OuterPosition', [10 35 190 175] );
uicontrol( 'Parent', f, ...
    'Units', 'Pixels', ...
    'Position', [5 5 90 25], ...
    'String', 'Button 1' );
uicontrol( 'Parent', f, ...
    'Units', 'Pixels', ...
    'Position', [105 5 90 25], ...
    'String', 'Button 2' );
```

.

Neither of these alternatives is particularly useful for a serious
user-interface. Typically there are user-interface components that should
be fixed size: icons, buttons, selectors etc; and others that should resize
with the window: graphs, images, prose text etc. To achieve this one needs
to be able to specify which interface components should be fixed size and
which variable. Over the last two decades, layouts have been shown to be
the method of choice for achieving this.

### For example:

Using layouts, some user-interface components scale with the window, others stay fixed

```
f = figure( 'Position', 200*ones(1,4) );
vbox = uiextras.VBox( 'Parent', f );
axes( 'Parent', vbox );
hbox = uiextras.HButtonBox( 'Parent', vbox, 'Padding', 5 );
uicontrol( 'Parent', hbox, ...
    'String', 'Button 1' );
uicontrol( 'Parent', hbox, ...
    'String', 'Button 2' );
set( vbox, 'Sizes', [-1 35] )
```

.

  

|  |  |  |  |  |
| --- | --- | --- | --- | --- |
|  | Layout heirarchies | [Top] | Positioning Axes |  |

© 2013 The MathWorks Ltd
• Terms of Use
• Patents
• Trademarks
