# Supplementary material for: Rapid Global Fitting of Large Fluorescence Lifetime Imaging Microscopy Datasets
Source: PLoS One. 2013 Aug 5;8(8):e70687. doi: 10.1371/journal.pone.0070687 (PMC3734241; doi:10.1371/journal.pone.0070687)
Supplement: File S1 — Compressed archive of the source code for FLIMfit used in the present study. Please note that up to date source code and compiled binaries are available from the Open Microscopy website [54]. (ZIP) [file pone.0070687.s001.zip › FLIMFit 4_3_3 Source/GlobalProcessingFrontEnd/HelperFunctions/GUILayout/layoutHelp/User guide2.html]

 


Positioning Axes


|  |  |
| --- | --- |
| **User guide** |  |

## 2: Positioning Axes

Unlike other MATLAB user interface components, `axes`
have two position properties: "Position" and "OuterPosition". This
means one has some extra options as to how the layout will
arrange the axes.

### Section contents:


- 2.1. Position vs OuterPosition
- 2.2. Axes inside layouts

  

|  |  |  |  |  |
| --- | --- | --- | --- | --- |
|  | Why use layouts? | [Top] | Position vs OuterPosition |  |

© 2013 The MathWorks Ltd
• Terms of Use
• Patents
• Trademarks
