# Supplementary material for: Rapid Global Fitting of Large Fluorescence Lifetime Imaging Microscopy Datasets
Source: PLoS One. 2013 Aug 5;8(8):e70687. doi: 10.1371/journal.pone.0070687 (PMC3734241; doi:10.1371/journal.pone.0070687)
Supplement: File S1 — Compressed archive of the source code for FLIMfit used in the present study. Please note that up to date source code and compiled binaries are available from the Open Microscopy website [54]. (ZIP) [file pone.0070687.s001.zip › FLIMFit 4_3_3 Source/GlobalProcessingFrontEnd/HelperFunctions/GUILayout/layoutHelp/User guide2_1.html]

Position vs OuterPosition


|  |  |
| --- | --- |
| **User guide** |  |

  

## 2.1: Position vs OuterPosition

Typically one
would position some axes using their "OuterPosition" so that the axis labels, title
and other annotations are all contained within the specified area. Sometimes,
particularly if drawing images, one might want to instead make the axes
canvas (the white bit!) fill the specified area. This is done by setting
the "Position" property instead.

### For example:

```
figure
axes( 'Units', 'Normalized', 'OuterPosition', [0 0 1 1] )
```

```
figure
axes( 'Units', 'Normalized', 'Position', [0 0 1 1] )
```

  

|  |  |  |  |  |
| --- | --- | --- | --- | --- |
|  | Positioning Axes | [Top] | Axes inside layouts |  |

© 2013 The MathWorks Ltd
• Terms of Use
• Patents
• Trademarks
