# Supplementary material for: Rapid Global Fitting of Large Fluorescence Lifetime Imaging Microscopy Datasets
Source: PLoS One. 2013 Aug 5;8(8):e70687. doi: 10.1371/journal.pone.0070687 (PMC3734241; doi:10.1371/journal.pone.0070687)
Supplement: File S1 — Compressed archive of the source code for FLIMfit used in the present study. Please note that up to date source code and compiled binaries are available from the Open Microscopy website [54]. (ZIP) [file pone.0070687.s001.zip › FLIMFit 4_3_3 Source/GlobalProcessingFrontEnd/HelperFunctions/GUILayout/layoutHelp/User guide2_2.html]

Axes inside layouts


|  |  |
| --- | --- |
| **User guide** |  |

  

## 2.2: Axes inside layouts

(The code for this example can be found here:
[ view
| edit
| run ]
)

When using layouts to position axes, the position property
is set by the layout, not the user. Whether the "Position"
or "OuterPosition" property is used is determined by the
"ActivePositionProperty" property of the `axes`.
Note that the default setting is "OuterPosition".

The following example illustrates the two usages.

#### Open a window

Open a new figure window and remove the toolbar and menus.

```
window = figure( 'Name', 'Axes inside layouts', ...
            'MenuBar', 'none', ...
            'Toolbar', 'none', ...
            'NumberTitle', 'off' );
```

#### Create the layout

The layout involves two axes side by side. This is done using a flexible horizontal box. The left-hand axes is left with the ActivePositionProperty set to "OuterPosition", but the right-hand axes is switched to use "Position".

```
hbox = uiextras.HBoxFlex('Parent', window, 'Spacing', 3);
axes1 = axes( 'Parent', hbox, ...
    'ActivePositionProperty', 'OuterPosition' );
axes2 = axes( 'Parent', hbox, ...
    'ActivePositionProperty', 'Position' );
set( hbox, 'Sizes', [-2 -1] );
```

#### Fill the axes

Using "OuterPosition" (left-hand axes) is the normal mode and looks good for virtually any plot type. Using "Position" is only really useful for 2D plots with the axes turned off, such as images.

```
x = membrane( 1, 15 );
surf( axes1, x );
lighting( axes1, 'gouraud' );
shading( axes1, 'interp' );
l = light( 'Parent', axes1 );
camlight( l, 'head' );
axis( axes1, 'tight' );

imagesc( x, 'Parent', axes2 );
set( axes2, 'xticklabel', [], 'yticklabel', [] );
```

  

|  |  |  |  |  |
| --- | --- | --- | --- | --- |
|  | Position vs OuterPosition | [Top] | Enable and Visible |  |

© 2013 The MathWorks Ltd
• Terms of Use
• Patents
• Trademarks
