# Supplementary material for: Rapid Global Fitting of Large Fluorescence Lifetime Imaging Microscopy Datasets
Source: PLoS One. 2013 Aug 5;8(8):e70687. doi: 10.1371/journal.pone.0070687 (PMC3734241; doi:10.1371/journal.pone.0070687)
Supplement: File S1 — Compressed archive of the source code for FLIMfit used in the present study. Please note that up to date source code and compiled binaries are available from the Open Microscopy website [54]. (ZIP) [file pone.0070687.s001.zip › FLIMFit 4_3_3 Source/GlobalProcessingFrontEnd/HelperFunctions/GUILayout/layoutHelp/User guide3.html]

 


Enable and Visible


|  |  |
| --- | --- |
| **User guide** |  |

## 3: Enable and Visible

The examples in this section show the effect of setting the
"Enable" or "Visible" properties on a layout object.

### Section contents:


- 3.1. Enable example
- 3.2. Visible example

  

|  |  |  |  |  |
| --- | --- | --- | --- | --- |
|  | Axes inside layouts | [Top] | Enable example |  |

© 2013 The MathWorks Ltd
• Terms of Use
• Patents
• Trademarks
