# Supplementary material for: Rapid Global Fitting of Large Fluorescence Lifetime Imaging Microscopy Datasets
Source: PLoS One. 2013 Aug 5;8(8):e70687. doi: 10.1371/journal.pone.0070687 (PMC3734241; doi:10.1371/journal.pone.0070687)
Supplement: File S1 — Compressed archive of the source code for FLIMfit used in the present study. Please note that up to date source code and compiled binaries are available from the Open Microscopy website [54]. (ZIP) [file pone.0070687.s001.zip › FLIMFit 4_3_3 Source/GlobalProcessingFrontEnd/HelperFunctions/GUILayout/layoutHelp/User guide3_1.html]

Enable example


|  |  |
| --- | --- |
| **User guide** |  |

  

## 3.1: Enable example

(The code for this example can be found here:
[ view
| edit
| run ]
)

This example shows how the `enable` property can be
used to indicate to users which bits of an interface are currently
relevant. This is particularly useful for preventing unwanted
clicking during long calculations, loading and saving!

Open a window and add a panel

```
fig = figure( 'Name', 'Enable example', ...
    'Position', [100 100 150 250], ...
    'MenuBar', 'none', ...
    'ToolBar', 'none', ...
    'NumberTitle', 'off' );
panel = uiextras.BoxPanel( 'Parent', fig, 'Title', 'Panel' );
```


Put some buttons inside the panel

```
box = uiextras.VButtonBox( 'Parent', panel );
uicontrol( 'Parent', box, 'String', 'Button 1' );
uicontrol( 'Parent', box, 'String', 'Button 2' );
uicontrol( 'Parent', box, 'String', 'Button 3', 'Enable', 'off' );
uicontrol( 'Parent', box, 'String', 'Button 4' );
uicontrol( 'Parent', box, 'String', 'Button 5', 'Enable', 'off' );
uicontrol( 'Parent', box, 'String', 'Button 6' );
```


Try disabling the panel

```
set( panel, 'Enable', 'off' );
```


Try enabling the panel. Note that the original "enable" state of each button is remembered.

```
set( panel, 'Enable', 'on' );
```

  

|  |  |  |  |  |
| --- | --- | --- | --- | --- |
|  | Enable and Visible | [Top] | Visible example |  |

© 2013 The MathWorks Ltd
• Terms of Use
• Patents
• Trademarks
