# Supplementary material for: Rapid Global Fitting of Large Fluorescence Lifetime Imaging Microscopy Datasets
Source: PLoS One. 2013 Aug 5;8(8):e70687. doi: 10.1371/journal.pone.0070687 (PMC3734241; doi:10.1371/journal.pone.0070687)
Supplement: File S1 — Compressed archive of the source code for FLIMfit used in the present study. Please note that up to date source code and compiled binaries are available from the Open Microscopy website [54]. (ZIP) [file pone.0070687.s001.zip › FLIMFit 4_3_3 Source/GlobalProcessingFrontEnd/HelperFunctions/GUILayout/layoutHelp/User guide4.html]

 


Using property defaults


|  |  |
| --- | --- |
| **User guide** |  |

## 4: Using property defaults

Sometimes it is useful to override the default values used when
new layout managers are created. The default property values for most
properties are inherited from the parent panel, figure or environemt
when the layout is first created. Functions for setting these are
described below.

### Section contents:


- 4.1. Setting defaults for one window
- 4.2. Setting defaults for all windows

  

|  |  |  |  |  |
| --- | --- | --- | --- | --- |
|  | Visible example | [Top] | Setting defaults for one window |  |

© 2013 The MathWorks Ltd
• Terms of Use
• Patents
• Trademarks
