# Supplementary material for: Rapid Global Fitting of Large Fluorescence Lifetime Imaging Microscopy Datasets
Source: PLoS One. 2013 Aug 5;8(8):e70687. doi: 10.1371/journal.pone.0070687 (PMC3734241; doi:10.1371/journal.pone.0070687)
Supplement: File S1 — Compressed archive of the source code for FLIMfit used in the present study. Please note that up to date source code and compiled binaries are available from the Open Microscopy website [54]. (ZIP) [file pone.0070687.s001.zip › FLIMFit 4_3_3 Source/GlobalProcessingFrontEnd/HelperFunctions/GUILayout/layoutHelp/User guide4_1.html]

Setting defaults for one window


|  |  |
| --- | --- |
| **User guide** |  |

  

## 4.1: Setting defaults for one window

The usual use of defaults is to modify the colors, fonts or
other settings used for a particular application window. The defaults
are therefore changed on the figure object using the `uiextras.set` function.

The example below sets new defaults for panel title-bar color and font
on the window before two panels are added.

```
f = figure();
uiextras.set( f, 'DefaultHBoxBackgroundColor', [0.3 0.3 0.3] );
uiextras.set( f, 'DefaultBoxPanelTitleColor', 'y' );
uiextras.set( f, 'DefaultBoxPanelFontSize', 16 );
h = uiextras.HBox( 'Parent', f, 'Padding', 10, 'Spacing', 10 );
p1 = uiextras.BoxPanel( 'Parent', h, 'Title', 'Panel 1' );
p2 = uiextras.BoxPanel( 'Parent', h, 'Title', 'Panel 2' );
```

  

|  |  |  |  |  |
| --- | --- | --- | --- | --- |
|  | Using property defaults | [Top] | Setting defaults for all windows |  |

© 2013 The MathWorks Ltd
• Terms of Use
• Patents
• Trademarks
