# Supplementary material for: Rapid Global Fitting of Large Fluorescence Lifetime Imaging Microscopy Datasets
Source: PLoS One. 2013 Aug 5;8(8):e70687. doi: 10.1371/journal.pone.0070687 (PMC3734241; doi:10.1371/journal.pone.0070687)
Supplement: File S1 — Compressed archive of the source code for FLIMfit used in the present study. Please note that up to date source code and compiled binaries are available from the Open Microscopy website [54]. (ZIP) [file pone.0070687.s001.zip › FLIMFit 4_3_3 Source/GlobalProcessingFrontEnd/HelperFunctions/GUILayout/layoutHelp/User guide4_2.html]

Setting defaults for all windows


|  |  |
| --- | --- |
| **User guide** |  |

  

## 4.2: Setting defaults for all windows

As well as modifying the defaults used in one window,
it is possible to override the defaults used for all windows. This makes use of
the special "0" handle that roughly corresponds to the MATLAB environment. Note, however,
that defaults set at the window level take precedence.

The example below shows how defaults can be set for the panel title-bar color
and font for all windows, but the font size is overridden for the second window:

```
uiextras.set( 0, 'DefaultHBoxBackgroundColor', [0.3 0.3 0.3] );
uiextras.set( 0, 'DefaultBoxPanelTitleColor', 'y' );
uiextras.set( 0, 'DefaultBoxPanelFontSize', 14 );
f1 = figure();
h = uiextras.HBox( 'Parent', f1, 'Padding', 10, 'Spacing', 10 );
p1 = uiextras.BoxPanel( 'Parent', h, 'Title', 'Panel 1' );
p2 = uiextras.BoxPanel( 'Parent', h, 'Title', 'Panel 2' );
f2 = figure();
uiextras.set( f2, 'DefaultBoxPanelFontSize', 20 );
p3 = uiextras.BoxPanel( 'Parent', f2, 'Title', 'Panel 3' );
```

It is important to note that although the default has been set
system-wide, it is not preserved between MATLAB sessions.

  

|  |  |  |  |  |
| --- | --- | --- | --- | --- |
|  | Setting defaults for one window | [Top] | Advanced maneuvers with panels |  |

© 2013 The MathWorks Ltd
• Terms of Use
• Patents
• Trademarks
