# Supplementary material for: Rapid Global Fitting of Large Fluorescence Lifetime Imaging Microscopy Datasets
Source: PLoS One. 2013 Aug 5;8(8):e70687. doi: 10.1371/journal.pone.0070687 (PMC3734241; doi:10.1371/journal.pone.0070687)
Supplement: File S1 — Compressed archive of the source code for FLIMfit used in the present study. Please note that up to date source code and compiled binaries are available from the Open Microscopy website [54]. (ZIP) [file pone.0070687.s001.zip › FLIMFit 4_3_3 Source/GlobalProcessingFrontEnd/HelperFunctions/GUILayout/layoutHelp/User guide5.html]

 


Advanced maneuvers with panels


|  |  |
| --- | --- |
| **User guide** |  |

## 5: Advanced maneuvers with panels

The `uiextras.BoxPanel` provides some extra buttons
and callbacks that can be used to design advanced user-interface
layouts. In particular, the sections below illustrate how to create
interfaces with context help, and panels that can be minimized or undocked.

### Section contents:


- 5.1. Context help
- 5.2. Minimize and maximize
- 5.3. Dock and undock

  

|  |  |  |  |  |
| --- | --- | --- | --- | --- |
|  | Setting defaults for all windows | [Top] | Context help |  |

© 2013 The MathWorks Ltd
• Terms of Use
• Patents
• Trademarks
