# Supplementary material for: Rapid Global Fitting of Large Fluorescence Lifetime Imaging Microscopy Datasets
Source: PLoS One. 2013 Aug 5;8(8):e70687. doi: 10.1371/journal.pone.0070687 (PMC3734241; doi:10.1371/journal.pone.0070687)
Supplement: File S1 — Compressed archive of the source code for FLIMfit used in the present study. Please note that up to date source code and compiled binaries are available from the Open Microscopy website [54]. (ZIP) [file pone.0070687.s001.zip › FLIMFit 4_3_3 Source/GlobalProcessingFrontEnd/HelperFunctions/GUILayout/layoutHelp/User guide5_1.html]

Context help


|  |  |
| --- | --- |
| **User guide** |  |

  

## 5.1: Context help

When a `uiextras.BoxPanel` has its "HelpFcn"
filled in, a help button () is shown in the upper-right of the
title-bar. When the user clicks this button the specified function
is called.

### For example:

Here we hookup the HelpFcn to simply bring up
the MATLAB documentation for each command. Clicking any of the "?" buttons
will cause the MATLAB help browser to open.

```
f = figure( 'Name', 'uiextras.BoxPanel Help Example' );
b = uiextras.HBox( 'Parent', f );
uiextras.BoxPanel( 'Parent', b, 'Title', 'sin', 'HelpFcn', @(a,b) doc('sin') );
uiextras.BoxPanel( 'Parent', b, 'Title', 'cos', 'HelpFcn', @(a,b) doc('cos') );
uiextras.BoxPanel( 'Parent', b, 'Title', 'tan', 'HelpFcn', @(a,b) doc('tan') );
```

  

|  |  |  |  |  |
| --- | --- | --- | --- | --- |
|  | Advanced maneuvers with panels | [Top] | Minimize and maximize |  |

© 2013 The MathWorks Ltd
• Terms of Use
• Patents
• Trademarks
