# Supplementary material for: Rapid Global Fitting of Large Fluorescence Lifetime Imaging Microscopy Datasets
Source: PLoS One. 2013 Aug 5;8(8):e70687. doi: 10.1371/journal.pone.0070687 (PMC3734241; doi:10.1371/journal.pone.0070687)
Supplement: File S1 — Compressed archive of the source code for FLIMfit used in the present study. Please note that up to date source code and compiled binaries are available from the Open Microscopy website [54]. (ZIP) [file pone.0070687.s001.zip › FLIMFit 4_3_3 Source/GlobalProcessingFrontEnd/HelperFunctions/GUILayout/layoutHelp/User guide5_2.html]

Minimize and maximize


|  |  |
| --- | --- |
| **User guide** |  |

  

## 5.2: Minimize and maximize

When a `uiextras.BoxPanel` has its "MinimizeFcn"
filled in, a minimize/maximize button ( / ) is shown in the upper-right of the
title-bar. When the user clicks this button the specified function
is called. Since the behaviour of the parent container is
different in different use-cases, it is up to the user to write
some code to actually resize the panel. Note that minimizing
a panel to its title-bar only really makes sense inside a
`uiextras.VBox` or `uiextras.VBoxFlex`.

The following simple example shows how to add minimize/maximize
functionality to a box full of panels. Save the code into
a file called "dockexample.m" to run it.

(The code for this example can be found here:
[ view
| edit
| run ]
)

#### Create the layout with three panels

Open a new figure window and add three panels.

```
function minimizeexample()
width = 200;
pheightmin = 20;
pheightmax = 100;

% Create the window and main layout
fig = figure( 'Name', 'Collapsable GUI example', ...
              'NumberTitle', 'off', ...
              'Toolbar', 'none', ...
              'MenuBar', 'none' );
box = uiextras.VBox( 'Parent', fig );

panel{1} = uiextras.BoxPanel( 'Title', 'Panel 1', 'Parent', box );
panel{2} = uiextras.BoxPanel( 'Title', 'Panel 2', 'Parent', box );
panel{3} = uiextras.BoxPanel( 'Title', 'Panel 3', 'Parent', box );
set( box, 'Sizes', pheightmax*ones(1,3) );

% Add some contents.
uicontrol( 'Style', 'PushButton', 'String', 'Button 1', 'Parent', panel{1} );
uicontrol( 'Style', 'PushButton', 'String', 'Button 2', 'Parent', panel{2} );
uicontrol( 'Style', 'PushButton', 'String', 'Button 3', 'Parent', panel{3} );

% Resize the window
pos = get( fig, 'Position' );
set( fig, 'Position', [pos(1,1:2),width,sum(box.Sizes)] );
```

#### Add the minimize/maximize callback

We set each panel to call the same minimize/maximize function.
This function is nested inside the main function so that it has access
to the main function's variables. A better way to do this is to make the
main function into a class, but this nested-function approach is fine
for simple applications.

Note that as soon as we set the "MinimizeFcn" property the minimize/maximize
icon appears in the top-right of each panel. We use a cell-array to pass an
extra argument, the panel number, to the minimize function. This extra argument appears after the usual
`eventSource` and `eventData` arguments.

```
% Hook up the minimize callback.
set( panel{1}, 'MinimizeFcn', {@nMinimize, 1} );
set( panel{2}, 'MinimizeFcn', {@nMinimize, 2} );
set( panel{3}, 'MinimizeFcn', {@nMinimize, 3} );

%-------------------------------------------------------------------------%  
 
    function nMinimize( eventSource, eventData, whichpanel )
        % A panel has been maximized/minimized
        s = get( box, 'Sizes' );
        pos = get( fig, 'Position' );
        panel{whichpanel}.IsMinimized = ~panel{whichpanel}.IsMinimized;
        if panel{whichpanel}.IsMinimized
            s(whichpanel) = pheightmin;
        else
            s(whichpanel) = pheightmax;
        end 
        set( box, 'Sizes', s );
        
        % Resize the figure, keeping the top stationary
        delta_height = pos(1,4) - sum( box.Sizes );
        set( fig, 'Position', pos(1,:) + [0 delta_height 0 -delta_height] );
    end % Minimize 

end % Main function
```

#### Click the minimize buttons

Minimizing the middle panel causes it to shrink to just its
title-bar and the window shrinks accordingly. The
"Minimize" icon is replaced by a "Maximise" icon.


Re-maximizing the panel would
cause it to re-appear in full and the window to grow again.

  

|  |  |  |  |  |
| --- | --- | --- | --- | --- |
|  | Context help | [Top] | Dock and undock |  |

© 2013 The MathWorks Ltd
• Terms of Use
• Patents
• Trademarks
