# Supplementary material for: Rapid Global Fitting of Large Fluorescence Lifetime Imaging Microscopy Datasets
Source: PLoS One. 2013 Aug 5;8(8):e70687. doi: 10.1371/journal.pone.0070687 (PMC3734241; doi:10.1371/journal.pone.0070687)
Supplement: File S1 — Compressed archive of the source code for FLIMfit used in the present study. Please note that up to date source code and compiled binaries are available from the Open Microscopy website [54]. (ZIP) [file pone.0070687.s001.zip › FLIMFit 4_3_3 Source/GlobalProcessingFrontEnd/HelperFunctions/GUILayout/layoutHelp/User guide6.html]

 


Using layouts inside GUIDE GUIs


|  |  |
| --- | --- |
| **User guide** |  |

## 6: Using layouts inside GUIDE GUIs

Whilst the layouts in this toolbox do not integrate into the
MATLAB Graphical User Interface Design Environment (`GUIDE`), it
is possible to insert layouts into a GUIDE-built GUI as follows:

#### Create the GUIDE GUI

Create you GUIDE application as usual, placing a panel where you
want to insert the layout. You can turn the panel border and title
off if you do not want them.

#### Insert the layout

Edit the "OpeningFcn" in the GUIDE-created code and insert your
layout into the panel, making it fill the space. In the example below
four boxpanels are inserted into a grid, which itself is placed inside
`uipanel1`:

```
% --- Executes just before guideApp is made visible. 
function guideApp_OpeningFcn(hObject, eventdata, handles, varargin)

% Choose default command line output for guideApp 
handles.output = hObject;

% Update handles structure 
guidata(hObject, handles);

% Put a layout in the panel 
g = uiextras.GridFlex( 'Parent', handles.uipanel1, ...
    'Units', 'Normalized', 'Position', [0 0 1 1], ...
    'Spacing', 5 );
uiextras.BoxPanel( 'Parent', g, 'Title', 'Panel 1' );
uiextras.BoxPanel( 'Parent', g, 'Title', 'Panel 2' );
uiextras.BoxPanel( 'Parent', g, 'Title', 'Panel 3' );
uiextras.BoxPanel( 'Parent', g, 'Title', 'Panel 4' );
g.RowSizes = [-1 -1];
```

(Full source code for this application is available here:
[ view
| edit
| run ]
)

  

|  |  |  |  |  |
| --- | --- | --- | --- | --- |
|  | Dock and undock | [Top] | Deploying GUIs using the MATLAB Compiler |  |

© 2013 The MathWorks Ltd
• Terms of Use
• Patents
• Trademarks
