# Supplementary material for: Rapid Global Fitting of Large Fluorescence Lifetime Imaging Microscopy Datasets
Source: PLoS One. 2013 Aug 5;8(8):e70687. doi: 10.1371/journal.pone.0070687 (PMC3734241; doi:10.1371/journal.pone.0070687)
Supplement: File S1 — Compressed archive of the source code for FLIMfit used in the present study. Please note that up to date source code and compiled binaries are available from the Open Microscopy website [54]. (ZIP) [file pone.0070687.s001.zip › FLIMFit 4_3_3 Source/GlobalProcessingFrontEnd/HelperFunctions/GUILayout/layoutHelp/User guide7.html]

 


Deploying GUIs using the MATLAB Compiler


|  |  |
| --- | --- |
| **User guide** |  |

## 7: Deploying GUIs using the MATLAB Compiler

Applications built using these layout tools can be deployed as standalone
executables using the MATLAB Compiler in
the same way as any other MATLAB application. There is, however, one thing to watch out for:

- You must explicitly include the uiextras.Resources folder

#### Example

Below is a screen-shot of the `deploytool` setup
used to build the example application.
The "Resources" folder from the toolbox has been explicitly added as a
shared resource so that the mouse-pointers and panel icons continue to work.


  

|  |  |  |  |  |
| --- | --- | --- | --- | --- |
|  | Using layouts inside GUIDE GUIs | [Top] | A complete example |  |

© 2013 The MathWorks Ltd
• Terms of Use
• Patents
• Trademarks
