# Supplementary material for: Rapid Global Fitting of Large Fluorescence Lifetime Imaging Microscopy Datasets
Source: PLoS One. 2013 Aug 5;8(8):e70687. doi: 10.1371/journal.pone.0070687 (PMC3734241; doi:10.1371/journal.pone.0070687)
Supplement: File S1 — Compressed archive of the source code for FLIMfit used in the present study. Please note that up to date source code and compiled binaries are available from the Open Microscopy website [54]. (ZIP) [file pone.0070687.s001.zip › FLIMFit 4_3_3 Source/GlobalProcessingFrontEnd/HelperFunctions/GUILayout/layoutHelp/User guide8.html]

 


A complete example


|  |  |
| --- | --- |
| **User guide** |  |

## 8: A complete example

The following example application uses many of the layout features
discussed above in order to create a good-looking user interface that
scales well when resized. It is not designed to showcase all the
layout functionality, but shows how callbacks are added to provide
user interaction. It also exemplifies separating the data from the GUI,
a fundamental part of creating modular and maintainable applications.

The full application is available here:

- View demoBrowser.m
- Edit demoBrowser.m
- Run demoBrowser.m

### Section contents:


- 8.1. Application structure
- 8.2. createInterface
- 8.3. updateInterface
- 8.4. onListSelection
- 8.5. Running it
- 8.6. Scalability

  

|  |  |  |  |  |
| --- | --- | --- | --- | --- |
|  | Deploying GUIs using the MATLAB Compiler | [Top] | Application structure |  |

© 2013 The MathWorks Ltd
• Terms of Use
• Patents
• Trademarks
