# Supplementary material for: Rapid Global Fitting of Large Fluorescence Lifetime Imaging Microscopy Datasets
Source: PLoS One. 2013 Aug 5;8(8):e70687. doi: 10.1371/journal.pone.0070687 (PMC3734241; doi:10.1371/journal.pone.0070687)
Supplement: File S1 — Compressed archive of the source code for FLIMfit used in the present study. Please note that up to date source code and compiled binaries are available from the Open Microscopy website [54]. (ZIP) [file pone.0070687.s001.zip › FLIMFit 4_3_3 Source/GlobalProcessingFrontEnd/HelperFunctions/GUILayout/layoutHelp/User guide8_2.html]

createInterface


|  |  |
| --- | --- |
| **User guide** |  |

  

## 8.2: createInterface

The interface creation is handled in the `createInterface`
subfunction. This has two distinct sections: menu building and widget arrangement.
The menus are built using the standard MATLAB menu building command `uimenu`,
so let's concentrate on the widget arrangement.

The top-level layout is a horizontal arrangement, placing the controls
to the left of the main plot. We make the layout draggable by using the
"flex" variant of HBox, and put a panel in each side. Note that setting the
"HelpFcn" for the view panel adds a small "?" icon for bringing up help. See
here for more details.

```
% Add the contents
mainLayout = uiextras.HBoxFlex( 'Parent', gui.Window, 'Spacing', 3 );

% Create the panels
controlPanel = uiextras.BoxPanel( ...
   'Parent', mainLayout, ...
   'Title', 'Select a demo:' );
gui.ViewPanel = uiextras.BoxPanel( ...
   'Parent', mainLayout, ...
   'Title', 'Viewing: ???', ...
   'HelpFcn', @onDemoHelp );

% Adjust the main layout  

set( mainLayout, 'Sizes', [-1,-2]  );
```

The controls panel is filled with a vertical layout containing the listbox
and a button. Note the callbacks that are specified for both the list and button. These
both call further subfunctions that are able to access the common "data" and "gui"
shared structures.

```
% Create the controls
controlLayout = uiextras.VBox( 'Parent', controlPanel, ...
   'Padding', 3, 'Spacing', 3 );
gui.ListBox = uicontrol( 'Style', 'list', ...
   'BackgroundColor', 'w', ...
   'Parent', controlLayout, ...
   'String', demoList(:), ...
   'Value', 1, ...
   'Callback', @onListSelection);
gui.HelpButton = uicontrol( 'Style', 'PushButton', ...
   'Parent', controlLayout, ...
   'String', 'Help for <demo>', ...
   'Callback', @onDemoHelp );
set( controlLayout, 'Sizes', [-1 28] ); % Make the list fill the space
```

Finally, the view itself is simply an axes placed inside the view panel:

```
% Create the view
gui.ViewAxes = axes( 'Parent', gui.ViewPanel );
```

(Full source code for this application is available here:
[ view
| edit
| run ]
)

  

|  |  |  |  |  |
| --- | --- | --- | --- | --- |
|  | Application structure | [Top] | updateInterface |  |

© 2013 The MathWorks Ltd
• Terms of Use
• Patents
• Trademarks
