# Supplementary material for: Rapid Global Fitting of Large Fluorescence Lifetime Imaging Microscopy Datasets
Source: PLoS One. 2013 Aug 5;8(8):e70687. doi: 10.1371/journal.pone.0070687 (PMC3734241; doi:10.1371/journal.pone.0070687)
Supplement: File S1 — Compressed archive of the source code for FLIMfit used in the present study. Please note that up to date source code and compiled binaries are available from the Open Microscopy website [54]. (ZIP) [file pone.0070687.s001.zip › FLIMFit 4_3_3 Source/GlobalProcessingFrontEnd/HelperFunctions/GUILayout/layoutHelp/User guide8_3.html]

updateInterface


|  |  |
| --- | --- |
| **User guide** |  |

  

## 8.3: updateInterface

The second subfunction we will look at, `updateInterface`, uses the
current selections to update the interface. This uses the structure
"data" to update various parts of the interface. For this simple example this just means:

**1. Update the selected item in the listbox**

```
set( gui.ListBox, 'Value', data.SelectedDemo );
```

**2. Update the help button label**

```
demoName = data.DemoNames{ data.SelectedDemo };  
set( gui.HelpButton, 'String', ['Help for ',demoName] );
```

**3. Update the view panel title**

```
set( gui.ViewPanel, 'Title', sprintf( 'Viewing: %s', demoName ) );
```

**4. Update the ticked menu**

```
menus = get( gui.ViewMenu, 'Children' );
set( menus, 'Checked', 'off' );
% Use the name to work out which menu item should be ticked
whichMenu = strcmpi( demoName, get( menus, 'Label' ) );
set( menus(whichMenu), 'Checked', 'on' );
```


In general, this update function is called whenever the underlying
shared "data" structure is changed. This happens when the user clicks
a button, selects a list item or a menu. Next we will look at a typical
callback.

(Full source code for this application is available here:
[ view
| edit
| run ]
)

  

|  |  |  |  |  |
| --- | --- | --- | --- | --- |
|  | createInterface | [Top] | onListSelection |  |

© 2013 The MathWorks Ltd
• Terms of Use
• Patents
• Trademarks
