# Supplementary material for: Rapid Global Fitting of Large Fluorescence Lifetime Imaging Microscopy Datasets
Source: PLoS One. 2013 Aug 5;8(8):e70687. doi: 10.1371/journal.pone.0070687 (PMC3734241; doi:10.1371/journal.pone.0070687)
Supplement: File S1 — Compressed archive of the source code for FLIMfit used in the present study. Please note that up to date source code and compiled binaries are available from the Open Microscopy website [54]. (ZIP) [file pone.0070687.s001.zip › FLIMFit 4_3_3 Source/GlobalProcessingFrontEnd/HelperFunctions/GUILayout/layoutHelp/User guide8_4.html]

onListSelection


|  |  |
| --- | --- |
| **User guide** |  |

  

## 8.4: onListSelection

Finally, let's have a look at how one of the callbacks works: "onListSelection", the function
that is called when an item is selected in the listbox. The other
callbacks obey the same pattern.

The basic rule of thumb is that a callback should not update any part of
the user interface directly - it's job is to respond to user interaction by changing the "data" structure.
In this example, each callback changes the underlying data structure
then asks the interface to refresh. This might mean that things update in the
interface that don't need to, but ensures the callbacks remain simple and that all
interface update logic is in one place. Extending this to more granular interface updates
is straightforward. See Scalability for more details.

For the listbox callback, the "src" argument is a handle to the
listbox and we simply need to update the SelectedDemo field of "data"
to reflect the new selection. We then ask the rest of the interface to
update in response to the change.

```
                % User selected a demo from the list - update "data" and refresh
data.SelectedDemo = get( src, 'Value' );
updateInterface();
redrawDemo();
```

(Full source code for this application is available here:
[ view
| edit
| run ]
)

  

|  |  |  |  |  |
| --- | --- | --- | --- | --- |
|  | updateInterface | [Top] | Running it |  |

© 2013 The MathWorks Ltd
• Terms of Use
• Patents
• Trademarks
