# Supplementary material for: Rapid Global Fitting of Large Fluorescence Lifetime Imaging Microscopy Datasets
Source: PLoS One. 2013 Aug 5;8(8):e70687. doi: 10.1371/journal.pone.0070687 (PMC3734241; doi:10.1371/journal.pone.0070687)
Supplement: File S1 — Compressed archive of the source code for FLIMfit used in the present study. Please note that up to date source code and compiled binaries are available from the Open Microscopy website [54]. (ZIP) [file pone.0070687.s001.zip › FLIMFit 4_3_3 Source/GlobalProcessingFrontEnd/HelperFunctions/GUILayout/layoutHelp/User guide8_5.html]

Running it


|  |  |
| --- | --- |
| **User guide** |  |

  

## 8.5: Running it

When the main function is launched (click
here)
it first creates the data, then the GUI, then updates the GUI using the data. At this point the
function exits and control is returned to the command prompt. Note, however,
that the GUI is still onscreen and will still respond to user interaction.

This works because the "shared" variables in the main function are not cleared when the
function exits. They are only cleared once the GUI is closed. This is a slightly
unusual, but very useful, feature of using nested functions for building
applications.

### For example:

```
>> demoBrowser
```

(Full source code for this application is available here:
[ view
| edit
| run ]
)

  

|  |  |  |  |  |
| --- | --- | --- | --- | --- |
|  | onListSelection | [Top] | Scalability |  |

© 2013 The MathWorks Ltd
• Terms of Use
• Patents
• Trademarks
