# Supplementary material for: Rapid Global Fitting of Large Fluorescence Lifetime Imaging Microscopy Datasets
Source: PLoS One. 2013 Aug 5;8(8):e70687. doi: 10.1371/journal.pone.0070687 (PMC3734241; doi:10.1371/journal.pone.0070687)
Supplement: File S1 — Compressed archive of the source code for FLIMfit used in the present study. Please note that up to date source code and compiled binaries are available from the Open Microscopy website [54]. (ZIP) [file pone.0070687.s001.zip › FLIMFit 4_3_3 Source/GlobalProcessingFrontEnd/HelperFunctions/GUILayout/layoutHelp/User guide8_6.html]

Scalability


|  |  |
| --- | --- |
| **User guide** |  |

  

## 8.6: Scalability

As applications get bigger the code gets more complex. The simple
application structure used here does not scale well to large applications,
however some small adjustments can make life much better:

- Convert the "data" structure into a handle object. This allows
  a single "data" object to be shared between mutliple graphical interfaces, and in turn means that the interface
  need not be built as a single monolithic entity.
- Use the events system to trigger updates
  to specific parts of the GUI in response to bits of the data object changing. This removes the need
  for a single large "UpdateInterface" function and reduces coupling between parts of the interface. For example,
  the "SelectedDemo" property would have an associated event such that when it is changed by a callback (or from the command-line) it
  notifies other interface components of the change. Each interface component (or group thereof) can
  just listen for the events that affect it.

Advice on how to build large-scale applications is beyond the scope
of this document. If you need help in this area, please contact your MathWorks
account manager who will be able to put you in touch with a technical specialist.

  

|  |  |  |  |  |
| --- | --- | --- | --- | --- |
|  | Running it | [Top] | Front page |  |

© 2013 The MathWorks Ltd
• Terms of Use
• Patents
• Trademarks
>
