# Supplementary material for: Rapid Global Fitting of Large Fluorescence Lifetime Imaging Microscopy Datasets
Source: PLoS One. 2013 Aug 5;8(8):e70687. doi: 10.1371/journal.pone.0070687 (PMC3734241; doi:10.1371/journal.pone.0070687)
Supplement: File S1 — Compressed archive of the source code for FLIMfit used in the present study. Please note that up to date source code and compiled binaries are available from the Open Microscopy website [54]. (ZIP) [file pone.0070687.s001.zip › FLIMFit 4_3_3 Source/GlobalProcessingFrontEnd/HelperFunctions/html/determine_photon_stats.html]

determine\_photon\_stats 

```
function [counts_per_photon offset] = determine_photon_stats(data,fit_offset,display_progress)

    if nargin < 2
        fit_offset = true;
    end

    if nargin < 3
        display_progress = false;
    end

    figure();
    ax = axes();

    % Number of points we will use to calculate the variance
    n_req = 100;

    % Number of time to repeat the calculation at diffent histogram
    % positions
    N = 50;

    % Reshape data if required, e.g. if images are passed
    sz = size(data);
    data = reshape(data,[sz(1) prod(sz(2:end))]);

    % Calculate intensity
    I = squeeze(sum(data,1));

    % Number of repeats for each different intensity
    n = length(I);

    % The fraction of those points we need either side of the chosen pt
    frac = n_req/n/2;

    % The histogram positions to use
    pos = linspace(0.2,0.8,N);

    h = waitbar(0,'Computing...');
    for i=1:N

        % select data around the histogram position
        centre = pos(i);
        Q = quantile(I,[centre-frac,centre+frac]);
        sel = I >= (Q(1)) & I <= (Q(2));
        sel_data = data(:,sel);
        sel_data = sel_data(2:end,:);

        c = 0;

        if fit_offset
            initial_guess = [1 1];
        else
            initial_guess = 1;
        end

        [o,f] = fminsearch(@objt,initial_guess);

        counts_per_photon(i) = o(1);
        if fit_offset
            offset(i) = o(2);
        else
            offset(i) = 0;
        end
        waitbar(i/N,h);
    end
    close(h);

    st = ['Counts per photon = ' num2str(mean(counts_per_photon),'%.2f') ' +/- ' num2str(std(counts_per_photon),2)];
    disp(st);
    disp(['           Offset = ' num2str(mean(offset),'%.2f') ' +/- ' num2str(std(offset),2)])

    hold(ax,'off')
    plot(ax,pos,counts_per_photon);
    xlabel(ax,'Sample')
    ylabel(ax,'Counts per photon');
    title(ax,st)

    counts_per_photon = mean(counts_per_photon);
    offset = mean(offset);

    function r = objt(m)

        if fit_offset
            offset = m(2);
        else
            offset = 0;
        end

        % Transform data
        tr = (sel_data-offset)/m(1);

        % Apply anscrombe transform
        antr = 2*sqrt(tr+3/8);

        % Calculate mean and std
        s = std(antr,0,2);
        mn = mean(antr,2);

        % Determine sum of square residuals
        r = norm(s-1).^2;

        if display_progress && mod(c,10) == 0
            hold(ax,'off');
            plot(ax,mn,ones(size(s)),'k');
            %ylim([0.8 1.2]);
            hold(ax,'on');
            plot(ax,mn,s.*s,'o');
            ylabel(ax,'Variance');
            xlabel(ax,'Corrected Photon Count');
            title(ax,['N = ' num2str(m(1)) ', Z = ' num2str(offset)]);
            drawnow;
        end
        c = c + 1;
    end

end
```

```
Error using determine_photon_stats (line 22)
Not enough input arguments.
```

Published with MATLAB® 7.13
