# Supplementary material for: Rapid Global Fitting of Large Fluorescence Lifetime Imaging Microscopy Datasets
Source: PLoS One. 2013 Aug 5;8(8):e70687. doi: 10.1371/journal.pone.0070687 (PMC3734241; doi:10.1371/journal.pone.0070687)
Supplement: File S1 — Compressed archive of the source code for FLIMfit used in the present study. Please note that up to date source code and compiled binaries are available from the Open Microscopy website [54]. (ZIP) [file pone.0070687.s001.zip › FLIMFit 4_3_3 Source/GlobalProcessingFrontEnd/HelperFunctions/xml_io_toos/html/xml_tutorial_script.html]

Tutorial for xml\_io\_tools Package 

# Tutorial for xml\_io\_tools Package

**By Jarek Tuszynski**

Package xml\_io\_tools can read XML files into MATLAB struct and writes MATLAB data types to XML files with help of simple interface to MATLAB's xmlwrite and xmlread functions.

Two function to simplify reading and writing XML files from MATLAB:

- Function xml\_read first calls MATLAB's xmlread function and than converts its output ('Document Object Model' tree of Java objects) to tree of MATLAB struct's. The output is in the format of nested structs and cells. In the output data structure field names are based on XML tags.

- Function xml\_write first convert input tree of MATLAB structs and cells and other types to tree of 'Document Object Model' nodes, and then writes resulting object to XML file using MATLAB's xmlwrite function. .

## Contents

- This package can:
- This package does not:
- Change History
- Licence
- Write XML file based on a Struct using "xml\_write"
- Read XML file producing a Struct using "xml\_read"
- "Pref.XmlEngine" flag in "xml\_write"
- Writing Struct with different type MATLAB arrays
- Read Struct with MATLAB arrays
- "Pref.StructItem" flag in "xml\_write" (controls 1D arrays of structs)
- "Pref.CellItem" flag in "xml\_write" (controls 1D arrays of cells)
- "Pref.NoCells" flag in "xml\_read"
- "Pref.ItemName" flag in "xml\_write" (customize 1D arrays of structs and cells)
- "Pref.ItemName" flag in "xml\_read"
- "Pref.CellItem" flag in "xml\_read"
- "Pref.CellTable" flag in "xml\_write" (controls 2D arrays of cells)
- Write 2D cell array in HTML format
- "Pref.StructTable" flag in "xml\_write" (controls 2D arrays of structs)
- "Pref.TableName" flag in "xml\_write" (controls encoding tags used for 2D arrays)
- "Pref.TableName" flag in "xml\_read"
- "Pref.Str2Num" flag in xml\_read (control conversion to numbers while reading)
- "Pref.PreserveSpace" flag in xml\_write (control handling of strings with leading/trailing spaces)
- "Pref.PreserveSpace" flag in xml\_read
- Write XML files with ATTRIBUTEs
- "Pref.Str2Num" flag in file with ATTRIBUTEs
- Write XML files with COMMENTs
- Write XML files with PROCESSING\_INSTRUCTIONs
- Write XML files with CDATA Sections
- Write XML files with special characters in TAG names
- Write XML files with Namespaces
- "Pref.KeepNS" flag in "xml\_read"
- Read XML files with special node types
- "Pref.ReadAttr" flag in "xml\_read" (control handling of nodes with attributes)
- "Pref.ReadSpec" flag in "xml\_read"
- "Pref.RootOnly" flag in "xml\_read"
- "Pref.RootOnly" flag in "xml\_write"
- "Pref.NumLevels" flag in "xml\_read"
- Create DOM object based on a Struct using "xml\_write"
- Convert DOM object to Struct using "xml\_read"
- Write XML file based on a DOM using "xml\_write\_xerces"
- Write XML to string instead of a file
- Write XML file with embedded binary data encoded as Base64 (using java version)
- Read XML file with embedded binary data encoded as Base64 (using java version)
- Write XML file with embedded binary data encoded as Base64 (simpler version using only matlab code
- Read XML file with embedded binary data encoded as Base64 (simpler version using only matlab code

## This package can:

- Read most XML files, created inside and outside of MATLAB environment, and convert them to MATLAB data structures.
- Write any MATLAB's struct tree to XML file
- Handle XML attributes and special XML nodes like comments, processing instructions and CDATA sections
- Supports base64 encoding and decoding to allow handling embeded binary data
- Be studied, modified, customized, rewritten and used in other packages without any limitations. All code is included and documented. Software is distributed under BSD Licence (included).

## This package does not:

- Guarantee to recover the same Matlab objects that were saved. If you need to be able to recover carbon copy of the structure that was saved than you will have to use one of the packages that uses special set of tags saved as xml attributes that help to guide the parsing of XML code. This package does not use those tags.
- Guarantee to work with older versions of MATLAB. Functions do not work with versions of MATLAB prior to 7.1 (26-Jul-2005).

## Change History

- 2006-11-06 - original version
- 2006-11-26 - corrected xml\_write to handle writing Matlab's column arrays to xml files. Bug discovered and diagnosed by Kalyan Dutta.
- 2006-11-28 - made changes to handle special node types like: COMMENTS and CDATA sections
- 2007-03-12 - Writing CDATA sections still did not worked. The problem was diagnosed and fixed by Alberto Amaro. The fix involved rewriting xmlwrite to use Apache Xerces java files directly instead of MATLAB's XMLUtils java class.
- 2007-06-21 - Fixed problem reported by Anna Kelbert in Reviews about not writing attributes of ROOT node. Also: added support for Processing Instructions, added support for global text nodes: Processing Instructions and comments, allowed writing tag names with special characters
- 2007-07-20 - Added tutorial script file. Extended support for global text nodes. Added more Preference fields.
- 2008-01-23 - Fixed problem reported by Anna Krewet of converting dates in format '2007-01-01' to numbers. Improved and added warning messages. Added detection of old Matlab versions incompatible with the library. Expanded documentation.
- 2008-06-23 - Fixed problem with writing 1D array reported by Mark Neil. Extended xml\_read's Pref.Num2Str to 3 settings (never, smart and always) for better control. Added parameter Pref.KeepNS for keeping or ignoring namespace data when reading. Fixed a bug related to writing 2D cell arrays brought up by Andrej's Mosat review.
- 2008-09-11 - Resubmitting last upload - zip file is still old
- 2009-02-26 - Small changes. More error handling. More robust in case of large binary objects. Added support for Base64 encoding/decoding of binary objects (using functions by Peter J. Acklam).
- 2009-06-26 - changes to xml\_read: added CellItem parameter to allow better control of reading files with 'item' notation (see comment by Shlomi); changed try-catch statements so xml\_read would work for mablab versions prior to 7.5 (see Thomas Pilutti comment)
- 2009-12-03 - added PreserveSpace parameter for contolling empty string handling as suggested by Sebastiaan. Fix suggested by Michael Murphy. Fixed number recognition code as suggested by Yuan Ren.
- 2010-05-04 - implemented fixes suggested by Dylan Reynolds from Airbus.
- 2010-07-28 - implemented support for 2D arrays of cells and structs suggested by Rodney Behn from MIT Lincoln Laboratory. Also attempted large scale cleanup of xml\_write function
- 2010-08-18 - minor extension to allow better handling of logical scalars and arrays and function handles suggested by Andreas Richter and others
- 2010-09-20 - allow reading and writing of sparse matrices. Improve reading of 1D boolean arrays.
- 2010-11-05 - Fix problem with empty cells reported by Richard Cotton; fixed issues with reading boolean arrays reported by Zohar Bar-Yehuda; Improved speed of base64 coding and decoding by switching to java based code.

## Licence

The package is distributed under BSD License

```
format compact; % viewing preference
clear variables;
type('license.txt')
```

```
Copyright (c) 2007, Jaroslaw Tuszynski
All rights reserved.

Redistribution and use in source and binary forms, with or without 
modification, are permitted provided that the following conditions are 
met:

    * Redistributions of source code must retain the above copyright 
      notice, this list of conditions and the following disclaimer.
    * Redistributions in binary form must reproduce the above copyright 
      notice, this list of conditions and the following disclaimer in 
      the documentation and/or other materials provided with the distribution
      
THIS SOFTWARE IS PROVIDED BY THE COPYRIGHT HOLDERS AND CONTRIBUTORS "AS IS" 
AND ANY EXPRESS OR IMPLIED WARRANTIES, INCLUDING, BUT NOT LIMITED TO, THE 
IMPLIED WARRANTIES OF MERCHANTABILITY AND FITNESS FOR A PARTICULAR PURPOSE 
ARE DISCLAIMED. IN NO EVENT SHALL THE COPYRIGHT OWNER OR CONTRIBUTORS BE 
LIABLE FOR ANY DIRECT, INDIRECT, INCIDENTAL, SPECIAL, EXEMPLARY, OR 
CONSEQUENTIAL DAMAGES (INCLUDING, BUT NOT LIMITED TO, PROCUREMENT OF 
SUBSTITUTE GOODS OR SERVICES; LOSS OF USE, DATA, OR PROFITS; OR BUSINESS 
INTERRUPTION) HOWEVER CAUSED AND ON ANY THEORY OF LIABILITY, WHETHER IN 
CONTRACT, STRICT LIABILITY, OR TORT (INCLUDING NEGLIGENCE OR OTHERWISE) 
ARISING IN ANY WAY OUT OF THE USE OF THIS SOFTWARE, EVEN IF ADVISED OF THE 
POSSIBILITY OF SUCH DAMAGE.
```

## Write XML file based on a Struct using "xml\_write"

Any MATLAB data struct can be saved to XML file.

```
MyTree=[];
MyTree.MyNumber = 13;
MyTree.MyString = 'Hello World';
xml_write('test.xml', MyTree);
type('test.xml')
```

```
<?xml version="1.0" encoding="utf-8"?>
<MyTree>
   <MyNumber>13</MyNumber>
   <MyString>Hello World</MyString>
</MyTree>
```

## Read XML file producing a Struct using "xml\_read"

```
[tree treeName] = xml_read ('test.xml');
disp([treeName{1} ' ='])
gen_object_display(tree)
```

```
MyTree =
    MyNumber: [13]
    MyString: 'Hello World'
```

## "Pref.XmlEngine" flag in "xml\_write"

Occasionaly some operations are performed better by Apache Xerces XML engine than default xmlread function. That is why xml\_write provide an option for choosing the underlaying xml engine. Code below performs the same operation as the previous section but using Apache Xerces XML engine. Notice that in this case name of root element was passed as variable and not extracted from the variable name.

```
Pref=[]; Pref.XmlEngine = 'Xerces';  % use Xerces xml generator directly
xml_write('test.xml', MyTree, 'TreeOfMine', Pref);
type('test.xml')
```

```
<?xml version="1.0" encoding="UTF-8"?>
<TreeOfMine>
    <MyNumber>13</MyNumber>
    <MyString>Hello World</MyString>
</TreeOfMine>
```

## Writing Struct with different type MATLAB arrays

```
MyTree=[];
MyTree.Empty   = [];                   % Empty variable
MyTree.Num_1x1 = 13;                   % simple scalar
MyTree.Vec_1x3 = [1 2 3];              % horizontal vector
MyTree.Vec_4x1 = [1; 2; 3; 4];         % vertical vector
MyTree.Mat_2x2 = [1, 2; 3, 4];         % 2D matrix
MyTree.Cube_3D = reshape(1:8,[2 2 2]); % 3D array
MyTree.String1 = '[2003 10 30]';       % number string with    [] brackets
MyTree.String2 = ' 2003 10 30 ';       % number string without [] brackets
MyTree.Logical_1x1 = false;            % single logical
MyTree.Logical_2x2 = [false, true; true, false]; % 2D matrix of logicals
MyTree.Logical_Str = 'False False	True True';
MyTree.Int_2x2 = uint8([1 2;3 4]);     % 2D matrix of uint8 integers
MyTree.Complex_1x1 = complex(1, 7);    % complex scalar
MyTree.Complex_2x2 = complex([1 2;3 4],[2 2;7 7]); % 2D matrix of complex numbers
MyTree.Sparse_9x9 = sparse(1:9,1:9,1); % sparse 9x9 matrix
MyTree.Function = @sum;                % function handle
xml_write('test.xml', MyTree);
type('test.xml')
```

```
<?xml version="1.0" encoding="utf-8"?>
<MyTree>
   <Empty/>
   <Num_1x1>13</Num_1x1>
   <Vec_1x3>[1 2 3]</Vec_1x3>
   <Vec_4x1>[1;2;3;4]</Vec_4x1>
   <Mat_2x2>[1 2;3 4]</Mat_2x2>
   <Cube_3D>[1;2;3;4;5;6;7;8]</Cube_3D>
   <String1>[2003 10 30]</String1>
   <String2>2003 10 30</String2>
   <Logical_1x1>[false]</Logical_1x1>
   <Logical_2x2>[false true;true false]</Logical_2x2>
   <Logical_Str>False False True True</Logical_Str>
   <Int_2x2>[uint8([1 2;3 4])]</Int_2x2>
   <Complex_1x1>1+i*7</Complex_1x1>
   <Complex_2x2>[1+i*2 2+i*2;3+i*7 4+i*7]</Complex_2x2>
   <Sparse_9x9>[sparse([1;2;3;4;5;6;7;8;9], [1;2;3;4;5;6;7;8;9], [1;1;1;1;1;1;1;1;1], 9, 9)]</Sparse_9x9>
   <Function>[@sum]</Function>
</MyTree>
```

## Read Struct with MATLAB arrays

Notice that 'Cube\_3D' did not preserve original dimentions

```
[tree treeName] = xml_read ('test.xml');
disp([treeName{1} ' ='])
gen_object_display(tree)
```

```
MyTree =
          Empty: [0x0 double]
        Num_1x1: [13]
        Vec_1x3: [1  2  3]
        Vec_4x1: [4x1 double]
        Mat_2x2: [2x2 double]
        Cube_3D: [8x1 double]
        String1: [2003    10    30]
        String2: [2003    10    30]
    Logical_1x1: false
    Logical_2x2: [2x2 logical]
    Logical_Str: [1x4 logical]
        Int_2x2: [2x2 double]
    Complex_1x1: [1+7i]
    Complex_2x2: [2x2 double]
     Sparse_9x9: [9x9 double]
       Function: [No method to display type]
```

## "Pref.StructItem" flag in "xml\_write" (controls 1D arrays of structs)

**Create a simple structure with 1D array of struct's**

```
MyTree = [];
MyTree.a(1).b = 'jack';
MyTree.a(2).b = 'john';
gen_object_display(MyTree)
```

```
    a: [1x2 struct]
       b: 'jack'

       b: 'john'
```

**Write XML with "StructItem = true" (default). Notice single 'a' section and multiple 'item' sub-sections. Those subsections are used to store array elements**

```
wPref.StructItem = true;
xml_write('test.xml', MyTree, 'MyTree',wPref);
type('test.xml')
fprintf('\nxml_read output:\n')
gen_object_display(xml_read ('test.xml'))
```

```
<?xml version="1.0" encoding="utf-8"?>
<MyTree>
   <a>
      <item>
         <b>jack</b>
      </item>
      <item>
         <b>john</b>
      </item>
   </a>
</MyTree>

xml_read output:
    a: [2x1 struct]
       b: 'jack'

       b: 'john'
```

**Write XML with "StructItem = false". Notice multiple 'a' sections**

```
wPref.StructItem = false;
xml_write('test.xml', MyTree, 'MyTree',wPref);
type('test.xml')
fprintf('\nxml_read output:\n')
gen_object_display(xml_read ('test.xml'))
```

```
<?xml version="1.0" encoding="utf-8"?>
<MyTree>
   <a>
      <b>jack</b>
   </a>
   <a>
      <b>john</b>
   </a>
</MyTree>

xml_read output:
    a: [2x1 struct]
       b: 'jack'

       b: 'john'
```

**Notice that xml\_read function produced the same struct when reading both files**

**Potential problems with "StructItem = true":**

```
wPref.StructItem = true;
MyTree1 = []; MyTree1.a.b    = 'jack';
MyTree2 = []; MyTree2.a(1).b = 'jack';
MyTree3 = []; MyTree3.a(2).b = 'jack';
xml_write('test.xml', MyTree1, [], wPref); type('test.xml');
xml_write('test.xml', MyTree2, [], wPref); type('test.xml');
xml_write('test.xml', MyTree3, [], wPref); type('test.xml');
```

```
<?xml version="1.0" encoding="utf-8"?>
<MyTree1>
   <a>
      <b>jack</b>
   </a>
</MyTree1>

<?xml version="1.0" encoding="utf-8"?>
<MyTree2>
   <a>
      <b>jack</b>
   </a>
</MyTree2>

<?xml version="1.0" encoding="utf-8"?>
<MyTree3>
   <a>
      <item>
         <b/>
      </item>
      <item>
         <b>jack</b>
      </item>
   </a>
</MyTree3>
```

**Notice that MyTree1 and MyTree2 produce identical files with no 'items', while MyTree2 and MyTree3 produce very different file structures. It was pointed out to me that files produced from MyTree2 and MyTree3 can not belong to the same schema, which can be a problem. The solution is to use cells.**

```
wPref.CellItem = true;
wPref.NoCells  = true;
MyTree2 = []; MyTree2.a{1}.b = 'jack';
MyTree3 = []; MyTree3.a{2}.b = 'jack';
xml_write('test.xml', MyTree2, [], wPref); type('test.xml');
xml_write('test.xml', MyTree3, [], wPref); type('test.xml');
```

```
<?xml version="1.0" encoding="utf-8"?>
<MyTree2>
   <a>
      <item>
         <b>jack</b>
      </item>
   </a>
</MyTree2>

<?xml version="1.0" encoding="utf-8"?>
<MyTree3>
   <a>
      <item/>
      <item>
         <b>jack</b>
      </item>
   </a>
</MyTree3>
```

## "Pref.CellItem" flag in "xml\_write" (controls 1D arrays of cells)

**Create a simple structure with cell arrays**

```
MyTree = [];
MyTree.a = {'jack', 'john'};
disp(MyTree)
```

```
    a: {'jack'  'john'}
```

**Write XML with "CellItem = true" (default). Notice single 'a' section and multiple 'item' sections**

```
Pref=[]; Pref.CellItem = true;
xml_write('test.xml', MyTree, 'MyTree',Pref);
type('test.xml')
fprintf('\nxml_read output:\n');
disp(xml_read ('test.xml'))
```

```
<?xml version="1.0" encoding="utf-8"?>
<MyTree>
   <a>
      <item>jack</item>
      <item>john</item>
   </a>
</MyTree>

xml_read output:
    a: {'jack'  'john'}
```

**Write XML with "CellItem = false". Notice multiple 'a' sections**

```
Pref=[]; Pref.CellItem = false;
xml_write('test.xml', MyTree, 'MyTree',Pref);
type('test.xml')
fprintf('\nxml_read output:\n');
disp(xml_read ('test.xml'))
```

```
<?xml version="1.0" encoding="utf-8"?>
<MyTree>
   <a>jack</a>
   <a>john</a>
</MyTree>

xml_read output:
    a: {'jack'  'john'}
```

**Notice that xml\_read function produced the same struct when reading both files**

## "Pref.NoCells" flag in "xml\_read"

**Create a cell/struct mixture object**

```
MyTree = [];
MyTree.a{1}.b = 'jack';
MyTree.a{2}.b = [];
MyTree.a{2}.c = 'john';
gen_object_display(MyTree);
```

```
    a: [1x2 cell] = 
       b: 'jack'

       b: [0x0 double]
       c: 'john'
```

**Save it to xml file**

```
Pref=[]; Pref.CellItem = false;
xml_write('test.xml', MyTree, 'MyTree',Pref);
type('test.xml')
```

```
<?xml version="1.0" encoding="utf-8"?>
<MyTree>
   <a>
      <b>jack</b>
   </a>
   <a>
      <b/>
      <c>john</c>
   </a>
</MyTree>
```

**Read above file with "Pref.NoCells=true" (default) - output is quite different then input** By default program is trying to convert everything to struct's and arrays of structs. In case arrays of structs all the structs in array need to have the same fields, and if they are not than MATLAB creates empty fields.

```
Pref=[]; Pref.NoCells=true;
gen_object_display(xml_read('test.xml', Pref))
```

```
    a: [2x1 struct]
       b: 'jack'
       c: [0x0 double]

       b: [0x0 double]
       c: 'john'
```

**Read above file with "Pref.NoCells=false" - now input and output are the same** Cell arrays of structs allow structs in array to have different fields.

```
Pref=[]; Pref.NoCells=false;
gen_object_display(xml_read('test.xml', Pref))
```

```
    a: [1x2 cell] = 
       b: 'jack'

       b: [0x0 double]
       c: 'john'
```

## "Pref.ItemName" flag in "xml\_write" (customize 1D arrays of structs and cells)

**Create a cell/struct mixture object**

```
MyTree = [];
MyTree.a{1}.b = 'jack';
MyTree.a{2}.c = 'john';
gen_object_display(MyTree);
```

```
    a: [1x2 cell] = 
       b: 'jack'

       c: 'john'
```

**Save it to xml file, using 'item' notation but with different name**

```
Pref=[];
Pref.CellItem = true;
Pref.ItemName = 'MyItem';
xml_write('test.xml', MyTree, 'MyTree',Pref);
type('test.xml')
```

```
<?xml version="1.0" encoding="utf-8"?>
<MyTree>
   <a>
      <MyItem>
         <b>jack</b>
      </MyItem>
      <MyItem>
         <c>john</c>
      </MyItem>
   </a>
</MyTree>
```

## "Pref.ItemName" flag in "xml\_read"

**Read above file with default settings ("Pref.ItemName = 'item'")** The results do not match the original structure

```
Pref=[]; Pref.NoCells  = false;
gen_object_display(xml_read('test.xml', Pref))
```

```
    a: [1x1 struct]
       MyItem: [1x2 cell] = 
               b: 'jack'

               c: 'john'
```

**Read above file with "Pref.ItemName = 'MyItem'" - now saved and read MATLAB structures are the same**

```
Pref=[];
Pref.ItemName = 'MyItem';
Pref.NoCells  = false;
gen_object_display(xml_read('test.xml', Pref))
```

```
    a: [1x2 cell] = 
       b: 'jack'

       c: 'john'
```

## "Pref.CellItem" flag in "xml\_read"

"Pref.ItemName" is used to create xml files with clearly marked arrays "Pref.CellItem" flag in "xml\_read" ensures that they are always read as arrays by forcing output to stay in cell format. In cell format s{1} is different than s, while s(1) is indistinguishable from s.

**Create a test file**

```
MyTree = [];
MyTree.a1{1}.b = 'jack'; % a1 - single struct
MyTree.a2{1}.b = 'jack'; % a2 - cell array of structs with the same fields
MyTree.a2{2}.b = 'john';
MyTree.a3{1}.b = 'jack'; % a3 - cell array of structs with the different fields
MyTree.a3{2}.c = 'john';
Pref=[];
Pref.CellItem = true;
Pref.Debug = true;
xml_write('test.xml', MyTree, 'MyTree',Pref);
type('test.xml')
```

```
<?xml version="1.0" encoding="utf-8"?>
<MyTree>
   <a1>
      <item>
         <b>jack</b>
      </item>
   </a1>
   <a2>
      <item>
         <b>jack</b>
      </item>
      <item>
         <b>john</b>
      </item>
   </a2>
   <a3>
      <item>
         <b>jack</b>
      </item>
      <item>
         <c>john</c>
      </item>
   </a3>
</MyTree>
```

**Read above file with "Pref.CellItem = true" (default)** All outputs are in cell format

```
Pref=[];
Pref.NoCells  = false;  % allow cell output
Pref.CellItem = true;   % keep 'item' arrays as cells
gen_object_display(xml_read('test.xml', Pref))
```

```
    a1: [1x1 cell] = 
        b: 'jack'

    a2: [1x1 cell] = 
            [1x1 struct]    [1x1 struct]

    a3: [1x1 cell] = 
            [1x1 struct]    [1x1 struct]
```

**Read above file with "Pref.CellItem = false"** Outputs format is determined by content

```
Pref=[];
Pref.NoCells  = false; % allow cell output
Pref.CellItem = false; % allow 'item' arrays to beheave like other fields
gen_object_display(xml_read('test.xml', Pref))
```

```
    a1: [1x1 struct]
        b: 'jack'
    a2: [2x1 struct]
        b: 'jack'

        b: 'john'

    a3: [1x2 cell] = 
        b: 'jack'

        c: 'john'
```

**Read above file with "Pref.CellItem = false" and "Pref.NoCells = true"** All outputs are in struct format

```
Pref=[];
Pref.NoCells  = true;  % don't allow cell output
Pref.CellItem = false; % allow 'item' arrays to beheave like other fields
gen_object_display(xml_read('test.xml', Pref))
```

```
    a1: [1x1 struct]
        b: 'jack'
    a2: [2x1 struct]
        b: 'jack'

        b: 'john'

    a3: [2x1 struct]
        b: 'jack'
        c: [0x0 double]

        b: [0x0 double]
        c: 'john'
```

## "Pref.CellTable" flag in "xml\_write" (controls 2D arrays of cells)

**Create a structure with 2D arrays of cells**

```
MyTree = [];
MyTree.M = {[1,2;3,4], 'M12'; struct('a','jack'), {11, 'N12'; 21, 'N22'}};
gen_object_display(MyTree)
```

```
    M: [2x2 cell] = 
           [2x2 double]    'M12'
           [1x1 struct]    {2x2 cell}
```

**Write XML with "CellTable = 'Html" (default). This option mimics use of HTML "tr" and "td" tags to encode 2D tables. Tag names can be changed using TableName parameter (see below)**

```
wPref = [];
wPref.CellTable = 'Html';
xml_write('test.xml', MyTree, 'MyTree',wPref);
type('test.xml')
fprintf('\nxml_read output:\n')
rPref=[]; rPref.NoCells=false;
gen_object_display(xml_read('test.xml', rPref))
```

```
<?xml version="1.0" encoding="utf-8"?>
<MyTree>
   <M>
      <tr>
         <td>[1 2;3 4]</td>
         <td>M12</td>
      </tr>
      <tr>
         <td>
            <a>jack</a>
         </td>
         <td>
            <tr>
               <td>11</td>
               <td>N12</td>
            </tr>
            <tr>
               <td>21</td>
               <td>N22</td>
            </tr>
         </td>
      </tr>
   </M>
</MyTree>

xml_read output:
    M: [2x2 cell] = 
           [2x2 double]    'M12'
           [1x1 struct]    {2x2 cell}
```

**Write XML with "CellTable = 'Vector'".** Converts 2D arrays to 1D array and item or regular notation. This option is mostly provided for backward compatibility since this was the behavior in prior verions of the code

```
wPref = [];
wPref.CellTable = 'Vector';
xml_write('test.xml', MyTree, 'MyTree',wPref);
type('test.xml')
fprintf('\nxml_read output:\n')
rPref=[]; rPref.NoCells=false;
gen_object_display(xml_read('test.xml', rPref))
```

```
<?xml version="1.0" encoding="utf-8"?>
<MyTree>
   <M>
      <item>[1 2;3 4]</item>
      <item>
         <a>jack</a>
      </item>
      <item>M12</item>
      <item>
         <item>11</item>
         <item>21</item>
         <item>N12</item>
         <item>N22</item>
      </item>
   </M>
</MyTree>

xml_read output:
    M: [1x4 cell] = 
            1     2
     3     4

       a: 'jack'

       M12

           [11]    [21]    'N12'    'N22'
```

**Create a simpler structure without struct's**

```
MyTree = [];
MyTree.M = {[1,2;3,4], 'M12'; 'M21', {11, 'N12'; 21, 'N22'}};
gen_object_display(MyTree)
```

```
    M: [2x2 cell] = 
           [2x2 double]    'M12'
           'M21'    {2x2 cell}
```

**Write XML with "CellTable = 'Matlab". This option encodes tables consisting of numbers, strings and other cell arrays as MATLAB command string. Unlike 'Html' option it does not work if one of the cells is a struct**

```
wPref = [];
wPref.CellTable = 'Matlab';
xml_write('test.xml', MyTree, 'MyTree',wPref);
type('test.xml')
fprintf('\nxml_read output:\n')
rPref=[]; rPref.NoCells=false;
gen_object_display(xml_read('test.xml', rPref))
```

```
<?xml version="1.0" encoding="utf-8"?>
<MyTree>
   <M>{[1 2;3 4],'M12';'M21',{11,'N12';21,'N22';};}</M>
</MyTree>

xml_read output:
    M: [2x2 cell] = 
           [2x2 double]    'M12'
           'M21'    {2x2 cell}
```

## Write 2D cell array in HTML format

```
MyTree = [];
MyTree.table.ATTRIBUTE.border=1;
MyTree.table.CONTENT = {'Apples', '44%'; 'Bannanas', '23%'; 'Oranges', '13%'; 'Other', '10%'};
xml_write('html/test.html', MyTree);
type('html/test.html')
```

```
<?xml version="1.0" encoding="utf-8"?>
<MyTree>
   <table border="1">
      <tr>
         <td>Apples</td>
         <td>44%</td>
      </tr>
      <tr>
         <td>Bannanas</td>
         <td>23%</td>
      </tr>
      <tr>
         <td>Oranges</td>
         <td>13%</td>
      </tr>
      <tr>
         <td>Other</td>
         <td>10%</td>
      </tr>
   </table>
</MyTree>
```

Click on test.html to opened this file with a web brouwser

## "Pref.StructTable" flag in "xml\_write" (controls 2D arrays of structs)

**Create a simple structure with arrays of struct's**

```
MyTree = [];
MyTree.a(1,1).b = 'jack';
MyTree.a(1,2).b = 'john';
MyTree.a(2,1).b = 'jim';
MyTree.a(2,2).b = 'jill';
gen_object_display(MyTree)
```

```
    a: [2x2 struct]
      a(1,1) =
        b: 'jack'
      a(1,2) =
        b: 'john'
      a(2,1) =
        b: 'jim'
      a(2,2) =
        b: 'jill'
```

**Write XML with "StructTable = 'Html" (default). This option mimics use of HTML "tr" and "td" tags to encode 2D tables. Tag names can be changed using TableName parameter (see below)**

```
wPref = [];
wPref.StructTable = 'Html';
xml_write('test.xml', MyTree, 'MyTree',wPref);
type('test.xml')
fprintf('\nxml_read output:\n')
gen_object_display(xml_read ('test.xml'))
```

```
<?xml version="1.0" encoding="utf-8"?>
<MyTree>
   <a>
      <tr>
         <td>
            <b>jack</b>
         </td>
         <td>
            <b>john</b>
         </td>
      </tr>
      <tr>
         <td>
            <b>jim</b>
         </td>
         <td>
            <b>jill</b>
         </td>
      </tr>
   </a>
</MyTree>

xml_read output:
    a: [2x2 struct]
      a(1,1) =
        b: 'jack'
      a(1,2) =
        b: 'john'
      a(2,1) =
        b: 'jim'
      a(2,2) =
        b: 'jill'
```

**Write XML with "CellTable = 'Vector'".** Converts 2D arrays to 1D array and item or regular notation. This option is mostly provided for backward compatibility since this was the behavior in prior verions of the code

```
wPref = [];
wPref.StructTable = 'Vector';
xml_write('test.xml', MyTree, 'MyTree',wPref);
type('test.xml')
fprintf('\nxml_read output:\n')
gen_object_display(xml_read ('test.xml'))
```

```
<?xml version="1.0" encoding="utf-8"?>
<MyTree>
   <a>
      <item>
         <b>jack</b>
      </item>
      <item>
         <b>jim</b>
      </item>
      <item>
         <b>john</b>
      </item>
      <item>
         <b>jill</b>
      </item>
   </a>
</MyTree>

xml_read output:
    a: [4x1 struct]
       b: 'jack'

       b: 'jim'

       b: 'john'

       b: 'jill'
```

## "Pref.TableName" flag in "xml\_write" (controls encoding tags used for 2D arrays)

**Create a cell object**

```
MyTree = [];
MyTree.M = {[1,2;3,4], 'M12'; 21, {11, 'N12'; 21, 'N22'}};
gen_object_display(MyTree);
```

```
    M: [2x2 cell] = 
           [2x2 double]    'M12'
           [21]    {2x2 cell}
```

**Save it to xml file, using 'Html' notation but with different names for rows and cells**

```
Pref=[]; Pref.TableName = {'row','cell'};
xml_write('test.xml', MyTree, 'MyTree',Pref);
type('test.xml')
```

```
<?xml version="1.0" encoding="utf-8"?>
<MyTree>
   <M>
      <row>
         <cell>[1 2;3 4]</cell>
         <cell>M12</cell>
      </row>
      <row>
         <cell>21</cell>
         <cell>
            <row>
               <cell>11</cell>
               <cell>N12</cell>
            </row>
            <row>
               <cell>21</cell>
               <cell>N22</cell>
            </row>
         </cell>
      </row>
   </M>
</MyTree>
```

## "Pref.TableName" flag in "xml\_read"

**Read above file with default settings ("Pref.TableName = {'tr','td'}")** The results do not match the original structure

```
Pref=[]; Pref.NoCells  = false;
gen_object_display(xml_read('test.xml', Pref))
```

```
    M: [1x1 struct]
       row: [2x1 struct]
            cell: [1x2 cell] = 
                       1     2
     3     4

                  M12


            cell: [1x2 cell] = 
                      21

                  row: [2x1 struct]
                       cell: [1x2 cell] = 
                                 11

                             N12


                       cell: [1x2 cell] = 
                                 21

                             N22
```

**Read above file with "Pref.TableName = {'row','cell'}" - now saved and read MATLAB structures are the same**

```
Pref=[];
Pref.TableName = {'row','cell'};
Pref.NoCells  = false;
gen_object_display(xml_read('test.xml', Pref))
```

```
    M: [2x2 cell] = 
           [2x2 double]    'M12'
           [21]    {2x2 cell}
```

## "Pref.Str2Num" flag in xml\_read (control conversion to numbers while reading)

**Create a cell/struct mixture object**

```
MyTree = [];
MyTree.str     = 'sphere';
MyTree.num1    =  123;
MyTree.num2    = '123';
MyTree.num3    = '[Inf,NaN]';
MyTree.calc    = '1+2+3+4';
MyTree.func    = 'sin(pi)/2';
MyTree.String1 = '[2003 10 30]';
MyTree.String2 = '2003 10 30';   % array resembling date
MyTree.ISO8601 = '2003-10-30';   % date in ISO 8601 format
MyTree.US_date = '2003/10/30';   % US style date format
MyTree.complex = '2003i-10e-30'; % complex number resembling a date
gen_object_display(MyTree);
```

```
        str: 'sphere'
       num1: [123]
       num2: '123'
       num3: '[Inf,NaN]'
       calc: '1+2+3+4'
       func: 'sin(pi)/2'
    String1: '[2003 10 30]'
    String2: '2003 10 30'
    ISO8601: '2003-10-30'
    US_date: '2003/10/30'
    complex: '2003i-10e-30'
```

**Save it to xml file**

```
xml_write('test.xml', MyTree);
type('test.xml')
```

```
<?xml version="1.0" encoding="utf-8"?>
<MyTree>
   <str>sphere</str>
   <num1>123</num1>
   <num2>123</num2>
   <num3>[Inf,NaN]</num3>
   <calc>1+2+3+4</calc>
   <func>sin(pi)/2</func>
   <String1>[2003 10 30]</String1>
   <String2>2003 10 30</String2>
   <ISO8601>2003-10-30</ISO8601>
   <US_date>2003/10/30</US_date>
   <complex>2003i-10e-30</complex>
</MyTree>
```

**Read above file with default settings** ("Pref.Str2Num = true" or "Pref.Str2Num = 'smart'"). Under this setting all strings that look like numbers are converted to numbers, except for strings that are recognized by MATLAB 'datenum' function as dates

```
gen_object_display(xml_read('test.xml'))
```

```
        str: 'sphere'
       num1: [123]
       num2: [123]
       num3: [Inf  NaN]
       calc: [10]
       func: 'sin(pi)/2'
    String1: [2003    10    30]
    String2: [2003    10    30]
    ISO8601: '2003-10-30'
    US_date: '2003/10/30'
    complex: [-1e-029+2003i]
```

**Note that all the fields of 'MyTree' can be converted to numbers (even 'sphere') but by default the function is trying to 'judge' if a string should be converted to a number or not**

```
MyCell = {'sphere','1+2+3+4','sin(pi)/2','2003 10 30','2003-10-30','2003/10/30','2003i-10e-30'};
cellfun(@str2num, MyCell, 'UniformOutput', false)
```

```
ans = 
  Columns 1 through 6
    [21x21 double]    [10]    [6.1232e-017]    [1x3 double]    [1963]    [6.6767]
  Column 7
    [-1.0000e-029 +2.0030e+003i]
```

**Read above file with "Pref.Str2Num = false" or "Pref.Str2Num = 'never'" to keep all the fields in string format**

```
Pref=[]; Pref.Str2Num = false;
gen_object_display(xml_read('test.xml', Pref))
```

```
        str: 'sphere'
       num1: '123'
       num2: '123'
       num3: '[Inf,NaN]'
       calc: '1+2+3+4'
       func: 'sin(pi)/2'
    String1: '[2003 10 30]'
    String2: '2003 10 30'
    ISO8601: '2003-10-30'
    US_date: '2003/10/30'
    complex: '2003i-10e-30'
```

**Read above file with "Pref.Str2Num = always" to convert all strings that look like numbers to numbers** note the likelly unintendet conversion of 'ISO8601'

```
Pref=[]; Pref.Str2Num   = 'always';
gen_object_display(xml_read('test.xml', Pref))
```

```
        str: 'sphere'
       num1: [123]
       num2: [123]
       num3: [Inf  NaN]
       calc: [10]
       func: 'sin(pi)/2'
    String1: [2003    10    30]
    String2: [2003    10    30]
    ISO8601: [1963]
    US_date: '2003/10/30'
    complex: [-1e-029+2003i]
```

**Notice that all three settings will produce the same output for "num1" and "num2" and there is no way to reproduce the original "MyTree" structure.**

## "Pref.PreserveSpace" flag in xml\_write (control handling of strings with leading/trailing spaces)

**Create a struct with strings**

```
MyTree=[];
MyTree.Empty     = '';
MyTree.OneSpace  = ' ';
MyTree.TwoSpaces = '  ';
MyTree.String1   = ' Hello      World ';
```

**Write XML with "PreserveSpace = false" (default).**

```
Pref=[]; Pref.PreserveSpace = false; % (default setting)
xml_write('test.xml', MyTree, [], Pref);
type('test.xml')
```

```
<?xml version="1.0" encoding="utf-8"?>
<MyTree>
   <Empty/>
   <OneSpace/>
   <TwoSpaces/>
   <String1>Hello World</String1>
</MyTree>
```

**Write XML with "PreserveSpace = true".**

```
Pref=[]; Pref.PreserveSpace = true;
xml_write('test.xml', MyTree, [], Pref);
type('test.xml')
```

```
<?xml version="1.0" encoding="utf-8"?>
<MyTree>
   <Empty/>
   <OneSpace> </OneSpace>
   <TwoSpaces>  </TwoSpaces>
   <String1> Hello      World </String1>
</MyTree>
```

## "Pref.PreserveSpace" flag in xml\_read

**Read file while using "PreserveSpace = false" (default).**

```
Pref=[]; Pref.PreserveSpace = false; % (default setting)
gen_object_display(xml_read('test.xml',Pref))
```

```
        Empty: [0x0 double]
     OneSpace: [0x0 double]
    TwoSpaces: [0x0 double]
      String1: 'Hello      World'
```

**Read file while using "PreserveSpace = true".**

```
Pref=[]; Pref.PreserveSpace = true;
gen_object_display(xml_read('test.xml',Pref))
```

```
        Empty: [0x0 double]
     OneSpace: ' '
    TwoSpaces: '  '
      String1: ' Hello      World '
```

## Write XML files with ATTRIBUTEs

In order to add node attributes a special ATTRIBUTE field is used. ATTRIBUTEs have to be of simple types like numbers or strings (not struct or cells). Attributes are easy to attach to structs nodes like MyTree below.

```
MyTree=[];
MyTree.MyNumber = 13;
MyTree.MyString = 'Hello World'; % simple case
MyTree.ATTRIBUTE.Num = 2;
xml_write('test.xml', MyTree);
type('test.xml')
```

```
<?xml version="1.0" encoding="utf-8"?>
<MyTree Num="2">
   <MyNumber>13</MyNumber>
   <MyString>Hello World</MyString>
</MyTree>
```

In case when one needs to attach attributes to nodes which are not structs (for example strings, numbers or calls) then special CONTENT field needs to be used to make the node a struct node.

```
MyTree=[];
MyTree.MyNumber = 13;
MyTree.MyString.CONTENT = 'Hello World'; % simple case
MyTree.MyString.ATTRIBUTE.Num = 2;
xml_write('test.xml', MyTree);
type('test.xml')
```

```
<?xml version="1.0" encoding="utf-8"?>
<MyTree>
   <MyNumber>13</MyNumber>
   <MyString Num="2">Hello World</MyString>
</MyTree>
```

## "Pref.Str2Num" flag in file with ATTRIBUTEs

**Create a cell/struct mixture object**

```
MyTree = [];
MyTree.X.ATTRIBUTE.str     = 'sphere';
MyTree.X.ATTRIBUTE.num1    =  123;
MyTree.X.ATTRIBUTE.num2    = '123';
MyTree.X.ATTRIBUTE.num3    = '[Inf,NaN]';
MyTree.X.ATTRIBUTE.calc    = '1+2+3+4';
MyTree.X.ATTRIBUTE.func    = 'sin(pi)/2';
MyTree.X.ATTRIBUTE.String1 = '[2003 10 30]';
MyTree.X.ATTRIBUTE.String2 = '2003 10 30';   % array resembling date
MyTree.X.ATTRIBUTE.ISO8601 = '2003-10-30';   % date in ISO 8601 format
MyTree.X.ATTRIBUTE.US_date = '2003/10/30';   % US style date format
MyTree.X.ATTRIBUTE.complex = '2003i-10e-30'; % complex number resembling a date
gen_object_display(MyTree);
```

```
    X: [1x1 struct]
       ATTRIBUTE: [1x1 struct]
                      str: 'sphere'
                     num1: [123]
                     num2: '123'
                     num3: '[Inf,NaN]'
                     calc: '1+2+3+4'
                     func: 'sin(pi)/2'
                  String1: '[2003 10 30]'
                  String2: '2003 10 30'
                  ISO8601: '2003-10-30'
                  US_date: '2003/10/30'
                  complex: '2003i-10e-30'
```

**Save it to xml file**

```
xml_write('test.xml', MyTree);
type('test.xml')
```

```
<?xml version="1.0" encoding="utf-8"?>
<MyTree>
   <X ISO8601="2003-10-30" String1="[2003 10 30]" String2="2003 10 30" US_date="2003/10/30" calc="1+2+3+4" complex="2003i-10e-30" func="sin(pi)/2" num1="123" num2="123" num3="[Inf,NaN]" str="sphere"/>
</MyTree>
```

**Read above file with default settings** ("Pref.Str2Num = true" or "Pref.Str2Num = 'smart'"). Under this setting all strings that look like numbers are converted to numbers, except for strings that are recognized by MATLAB 'datenum' function as dates

```
gen_object_display(xml_read('test.xml'))
```

```
    X: [1x1 struct]
         CONTENT: [0x0 double]
       ATTRIBUTE: [1x1 struct]
                  ISO8601: '2003-10-30'
                  String1: '[2003 10 30]'
                  String2: '2003 10 30'
                  US_date: '2003/10/30'
                     calc: '1+2+3+4'
                  complex: [-1e-029+2003i]
                     func: 'sin(pi)/2'
                     num1: [123]
                     num2: [123]
                     num3: '[Inf,NaN]'
                      str: 'sphere'
```

**Read above file with "Pref.Str2Num = false" or "Pref.Str2Num = 'never'" to keep all the fields in string format**

```
Pref=[]; Pref.Str2Num = false;
gen_object_display(xml_read('test.xml', Pref))
```

```
    X: [1x1 struct]
         CONTENT: [0x0 double]
       ATTRIBUTE: [1x1 struct]
                  ISO8601: '2003-10-30'
                  String1: '[2003 10 30]'
                  String2: '2003 10 30'
                  US_date: '2003/10/30'
                     calc: '1+2+3+4'
                  complex: '2003i-10e-30'
                     func: 'sin(pi)/2'
                     num1: '123'
                     num2: '123'
                     num3: '[Inf,NaN]'
                      str: 'sphere'
```

**Read above file with "Pref.Str2Num = always" to convert all strings that look like numbers to numbers**

```
Pref=[]; Pref.Str2Num   = 'always';
gen_object_display(xml_read('test.xml', Pref))
```

```
    X: [1x1 struct]
         CONTENT: [0x0 double]
       ATTRIBUTE: [1x1 struct]
                  ISO8601: '2003-10-30'
                  String1: '[2003 10 30]'
                  String2: '2003 10 30'
                  US_date: '2003/10/30'
                     calc: '1+2+3+4'
                  complex: [-1e-029+2003i]
                     func: 'sin(pi)/2'
                     num1: [123]
                     num2: [123]
                     num3: '[Inf,NaN]'
                      str: 'sphere'
```

**Notice that all three settings will produce the same output for "num1" and "num2" and there is no way to reproduce the original "MyTree" structure.**

## Write XML files with COMMENTs

Insertion of Comments is done with help of special COMMENT field. Note that MATLAB's xmlwrite is less readable due to lack of end-of-line characters around comment section.

```
MyTree=[];
MyTree.COMMENT = 'This is a comment';
MyTree.MyNumber = 13;
MyTree.MyString.CONTENT = 'Hello World';
xml_write('test.xml', MyTree);
type('test.xml')
```

```
<?xml version="1.0" encoding="utf-8"?>
<MyTree><!--This is a comment-->
   <MyNumber>13</MyNumber>
   <MyString>Hello World</MyString>
</MyTree>
```

**Same operation using Apache Xerces XML engine** gives the same result

```
Pref=[]; Pref.XmlEngine = 'Xerces';  % use Xerces xml generator directly
xml_write('test.xml', MyTree, 'MyTree', Pref);
type('test.xml')
```

```
<?xml version="1.0" encoding="UTF-8"?>
<MyTree>
    <!--This is a comment-->
    <MyNumber>13</MyNumber>
    <MyString>Hello World</MyString>
</MyTree>
```

**Comments in XML top level (method #1)** This method uses cell array

```
MyTree=[];
MyTree.MyNumber = 13;
MyTree.MyString = 'Hello World';
xml_write('test.xml', MyTree, {'MyTree', [], 'This is a global comment'});
type('test.xml')
```

```
<?xml version="1.0" encoding="utf-8"?><!--This is a global comment-->
<MyTree>
   <MyNumber>13</MyNumber>
   <MyString>Hello World</MyString>
</MyTree>
```

**Same operation using Apache Xerces XML engine** gives even nicer results.

```
Pref=[]; Pref.XmlEngine = 'Xerces';  % use Xerces xml generator directly
xml_write('test.xml', MyTree, {'MyTree', [], 'This is a global comment'}, Pref);
type('test.xml')
```

```
<?xml version="1.0" encoding="UTF-8"?>
<!--This is a global comment-->
<MyTree>
    <MyNumber>13</MyNumber>
    <MyString>Hello World</MyString>
</MyTree>
```

**Comments in XML top level (method #2)** This method adds an extra top layer to the struct 'tree' and sets "Pref.RootOnly = false", which informs the function about the extra layer. Notice that RootName is also saved as a part of the 'tree', and does not have to be passed in separately.

```
MyTree=[];
MyTree.COMMENT = 'This is a global comment';
MyTree.MyTest.MyNumber = 13;
MyTree.MyTest.MyString = 'Hello World';
Pref=[]; Pref.RootOnly = false;
xml_write('test.xml', MyTree, [], Pref);
type('test.xml')
```

```
<?xml version="1.0" encoding="utf-8"?><!--This is a global comment-->
<MyTest>
   <MyNumber>13</MyNumber>
   <MyString>Hello World</MyString>
</MyTest>
```

**Same operation using Apache Xerces XML engine**

```
Pref=[]; Pref.XmlEngine = 'Xerces';  % use Xerces xml generator directly
Pref.RootOnly  = false;
xml_write('test.xml', MyTree, [], Pref);
type('test.xml')
```

```
<?xml version="1.0" encoding="UTF-8"?>
<!--This is a global comment-->
<MyTest>
    <MyNumber>13</MyNumber>
    <MyString>Hello World</MyString>
</MyTest>
```

## Write XML files with PROCESSING\_INSTRUCTIONs

Insertion of Processing Instructions is done through use of special PROCESSING\_INSTRUCTION field, which stores the instruction string. The string has to be in 'target data' format separated by space.

```
MyTree=[];
MyTree.PROCESSING_INSTRUCTION = 'xml-stylesheet type="a" href="foo"';
MyTree.MyNumber = 13;
MyTree.MyString = 'Hello World';
xml_write('test.xml', MyTree);
type('test.xml')
```

```
<?xml version="1.0" encoding="utf-8"?>
<MyTree><?xml-stylesheet type="a" href="foo"?>
   <MyNumber>13</MyNumber>
   <MyString>Hello World</MyString>
</MyTree>
```

**Same operation using Apache Xerces XML engine**

```
Pref=[]; Pref.XmlEngine = 'Xerces';  % use Xerces xml generator directly
xml_write('test.xml', MyTree, 'MyTree', Pref);
type('test.xml')
```

```
<?xml version="1.0" encoding="UTF-8"?>
<MyTree><?xml-stylesheet type="a" href="foo"?>
    <MyNumber>13</MyNumber>
    <MyString>Hello World</MyString>
</MyTree>
```

**PROCESSING\_INSTRUCTIONs in XML top level (method #1)** This method uses cell array

```
MyTree=[];
MyTree.MyNumber = 13;
MyTree.MyString = 'Hello World';
xml_write('test.xml', MyTree, {'MyTree', 'xml-stylesheet type="a" href="foo"'});
type('test.xml')
```

```
<?xml version="1.0" encoding="utf-8"?><?xml-stylesheet type="a" href="foo"?>
<MyTree>
   <MyNumber>13</MyNumber>
   <MyString>Hello World</MyString>
</MyTree>
```

**Same operation using Apache Xerces XML engine**

```
Pref=[]; Pref.XmlEngine = 'Xerces';  % use Xerces xml generator directly
xml_write('test.xml', MyTree, {'MyTree', 'xml-stylesheet type="a" href="foo"'}, Pref);
type('test.xml')
```

```
<?xml version="1.0" encoding="UTF-8"?>
<?xml-stylesheet type="a" href="foo"?>
<MyTree>
    <MyNumber>13</MyNumber>
    <MyString>Hello World</MyString>
</MyTree>
```

**PROCESSING\_INSTRUCTIONs in XML top level (method #2)** This method adds an extra top layer to the struct 'tree' and sets pref.RootOnly=false, which informs the function about the extra layer. Notice that RootName is also saved as a part of the 'tree', and does not have to be passed in separately.

```
MyTree=[];
MyTree.PROCESSING_INSTRUCTION =  'xml-stylesheet type="a" href="foo"';
MyTree.MyTest.MyNumber = 13;
MyTree.MyTest.MyString = 'Hello World';
Pref=[]; Pref.RootOnly = false;
xml_write('test.xml', MyTree, [], Pref);
type('test.xml')
```

```
<?xml version="1.0" encoding="utf-8"?><?xml-stylesheet type="a" href="foo"?>
<MyTest>
   <MyNumber>13</MyNumber>
   <MyString>Hello World</MyString>
</MyTest>
```

**Same operation using Apache Xerces XML engine**

```
Pref=[]; Pref.XmlEngine = 'Xerces';  % use Xerces xml generator directly
Pref.RootOnly  = false;
xml_write('test.xml', MyTree, 'MyTree', Pref);
type('test.xml')
```

```
<?xml version="1.0" encoding="UTF-8"?>
<?xml-stylesheet type="a" href="foo"?>
<MyTest>
    <MyNumber>13</MyNumber>
    <MyString>Hello World</MyString>
</MyTest>
```

## Write XML files with CDATA Sections

"In an XML document a CDATA (Character DATA) section is a section of element content that is marked for the parser to interpret as only character data, not markup." (from Wikipedia) To insert CDATA Sections one use special CDATA\_SECTION field, which stores the instruction string. Note that MATLAB's xmlwrite created wrong xml code for CDATA section

```
MyTree=[];
MyTree.CDATA_SECTION = '<A>txt</A>';
MyTree.MyNumber = 13;
MyTree.MyString = 'Hello World';
xml_write('test.xml', MyTree);
type('test.xml')
```

```
<?xml version="1.0" encoding="utf-8"?>
<MyTree>&lt;A&gt;txt&lt;/A&gt;<MyNumber>13</MyNumber>
   <MyString>Hello World</MyString>
</MyTree>
```

**Same operation using Apache Xerces XML engine produces correct results**

```
Pref=[]; Pref.XmlEngine = 'Xerces';  % use Xerces xml generator directly
xml_write('test.xml', MyTree, 'MyTree', Pref);
type('test.xml')
```

```
<?xml version="1.0" encoding="UTF-8"?>
<MyTree><![CDATA[<A>txt</A>]]><MyNumber>13</MyNumber>
    <MyString>Hello World</MyString>
</MyTree>
```

## Write XML files with special characters in TAG names

The input to xml\_write requires that all tags one wants in XML document have to be encoded as field names of MATLAB's struct's. Matlab has a lot of restrictions on variable names. This section is about XML tags with names not allowed as MATLAB variables, or more specifically with characters allowed as xml tag names but not allowed as MATLAB variable names. Characters like that can be replaced by their hexadecimal representation just as it is done by genvarname function. Alternative way of writing the first example is:

```
MyTree=[];
MyTree.('MyNumber') = 13;               % same as MyTree.MyNumber = 13;
MyTree.MyString.CONTENT = 'Hello World';
MyTree.MyString.ATTRIBUTE.('Num') = 2;  % same as MyTree.MyString.ATTRIBUTE.Num = 2;
xml_write('test.xml', MyTree);
type('test.xml')
```

```
<?xml version="1.0" encoding="utf-8"?>
<MyTree>
   <MyNumber>13</MyNumber>
   <MyString Num="2">Hello World</MyString>
</MyTree>
```

**This approach fails for some characters like dash '-', colon ':', and international characters.**

```
MyTree=[];
try
  MyTree.('My-Number') = 13;
  MyTree.MyString.CONTENT = 'Hello World';
  MyTree.MyString.ATTRIBUTE.('Num_ö') = 2;
catch  %#ok<CTCH>
  err = lasterror; %#ok<LERR>
  disp(err.message);
end
```

```
Invalid field name: 'My-Number'.
```

It can be overcome by replacing offending characters with their hexadecimal representation. That can be done manually or with use of genvarname function. Note that MATLAB 'type' function does not show correctly 'ö' letter in xml file, but opening the file in editor shows that it is correct.

```
MyTree=[];
MyTree.(genvarname('My-Number')) = 13;
MyTree.MyString.CONTENT = 'Hello World';
MyTree.MyString.ATTRIBUTE.Num_0xF6 = 2;
gen_object_display(MyTree);
xml_write('test.xml', MyTree);
type('test.xml')
```

```
    My0x2DNumber: [13]
        MyString: [1x1 struct]
                    CONTENT: 'Hello World'
                  ATTRIBUTE: [1x1 struct]
                             Num_0xF6: [2]

<?xml version="1.0" encoding="utf-8"?>
<MyTree>
   <My-Number>13</My-Number>
   <MyString Num_Ã¶="2">Hello World</MyString>
</MyTree>
```

**Also two of the characters '-' and ':' can be encoded by a special strings: '\_DASH\_' and '\_COLON\_' respectively**

```
MyTree=[];
MyTree.My_DASH_Number = 13;
MyTree.MyString.CONTENT = 'Hello World';
MyTree.MyString.ATTRIBUTE.Num0xF6 = 2;
xml_write('test.xml', MyTree);
type('test.xml')
```

```
<?xml version="1.0" encoding="utf-8"?>
<MyTree>
   <My-Number>13</My-Number>
   <MyString NumÃ¶="2">Hello World</MyString>
</MyTree>
```

## Write XML files with Namespaces

No extra special fields are needed to define XML namespaces, only colon character written using '0x3A' or '\_COLON\_'. Below is an example of a namespace definition

```
MyTree=[];
MyTree.f_COLON_child.ATTRIBUTE.xmlns_COLON_f = 'http://www.foo.com';
MyTree.f_COLON_child.f_COLON_MyNumber = 13;
MyTree.f_COLON_child.f_COLON_MyString = 'Hello World';
xml_write('test.xml', MyTree, 'MyTree');
type('test.xml')
```

```
<?xml version="1.0" encoding="utf-8"?>
<MyTree>
   <f:child xmlns:f="http://www.foo.com">
      <f:MyNumber>13</f:MyNumber>
      <f:MyString>Hello World</f:MyString>
   </f:child>
</MyTree>
```

**Same operation using Apache Xerces XML engine**

```
Pref=[]; Pref.XmlEngine = 'Xerces';  % use Xerces xml generator directly
xml_write('test.xml', MyTree, 'f_COLON_MyTree', Pref);
type('test.xml')
```

```
<?xml version="1.0" encoding="UTF-8"?>
<f:MyTree>
    <f:child xmlns:f="http://www.foo.com">
        <f:MyNumber>13</f:MyNumber>
        <f:MyString>Hello World</f:MyString>
    </f:child>
</f:MyTree>
```

## "Pref.KeepNS" flag in "xml\_read"

Thise option allow keeping or exclusion of namespaces in tag names. By default the namespace data is kept but it produces much longer field names in the output structure. Ignoring namespace will produce more readible output. Perform default read of file with namespace

```
tree = xml_read('test.xml');
gen_object_display(tree);
```

```
    f_COLON_child: [1x1 struct]
                   f_COLON_MyNumber: [13]
                   f_COLON_MyString: 'Hello World'
                          ATTRIBUTE: [1x1 struct]
                                     xmlns_COLON_f: 'http://www.foo.com'
```

Now the same operation with KeepNS = false.

```
Pref=[]; Pref.KeepNS = false; % do not read attributes
tree = xml_read('test.xml', Pref);
gen_object_display(tree);
```

```
    child: [1x1 struct]
            MyNumber: [13]
            MyString: 'Hello World'
           ATTRIBUTE: [1x1 struct]
                      f: 'http://www.foo.com'
```

## Read XML files with special node types

Display and read the file, then show the data structure. Note that MATLAB 'type' function shows 'ö' letter incorrectly as 'A¶' in xml file, but opening the file in editor shows that it is correct.

```
fprintf('Test xml file:\n');
type('test_file.xml')
```

```
Test xml file:

<?xml version="1.0" encoding="utf-8" ?> 
<?xml-stylesheet type="text/css" href="foo.css"?>
<!-- This is a Global Comment -->
<aaa xmlns:xsi="http://www.foo.org">
  <?ProcInst type="local processing instruction"?>
  <!-- local comment 1 -->
  bbb
  <!-- local comment 2 -->
  ccc
  <matrix bad-name='fff'>
    5e3+2*i, Inf
    NaN,     pi
  </matrix>
  <ee_e> ee_e </ee_e>
  <ff-f> ff-f </ff-f>
  <ggÃ¶g> ggÃ¶g </ggÃ¶g>
  <![CDATA[
    Here <ddd>xml</ddd> tags are treated as ...
    ... text
	]]>
</aaa>
```

Read only the Root Element (default)

```
[tree GlobalTextNodes] = xml_read('test_file.xml');
fprintf('Global Data (Root name, Global Processing Instructions and Global Comments):\n');
disp(GlobalTextNodes')
fprintf('\nStructure read from the file (uncludes COMMENT and CDATA sections):\n');
gen_object_display(tree);
```

```
Global Data (Root name, Global Processing Instructions and Global Comments):
    'aaa'
    'xml-stylesheet type="text/css" href="foo.css"'
    'This is a Global Comment'

Structure read from the file (uncludes COMMENT and CDATA sections):
    PROCESSING_INSTRUCTION: 'ProcInst type="local processing instruction"'
                   COMMENT: [1x2 cell] = 
                            local comment 1

                            local comment 2

                   CONTENT: [1x2 cell] = 
                            bbb

                            ccc

                    matrix: [1x1 struct]
                              CONTENT: [2x2 double]
                            ATTRIBUTE: [1x1 struct]
                                       bad_DASH_name: 'fff'
                      ee_e: 'ee_e'
                 ff_DASH_f: 'ff-f'
                   gg0xF6g: 'ggög'
             CDATA_SECTION: 'Here <ddd>xml</ddd> tags are treated as ...
    ... text'
                 ATTRIBUTE: [1x1 struct]
                            xmlns_COLON_xsi: 'http://www.foo.org'
```

Read the whole tree including global Comments and Processing Instructions

```
Pref=[]; Pref.RootOnly = false;
[tree GlobalTextNodes] = xml_read('test_file.xml', Pref);
fprintf('Global Data (Root name, Global Processing Instructions and Global Comments):\n');
disp(GlobalTextNodes')
fprintf('\nStructure read from the file (uncludes COMMENT and CDATA sections):\n');
gen_object_display(tree);
```

```
Global Data (Root name, Global Processing Instructions and Global Comments):
    'aaa'
    'xml-stylesheet type="text/css" href="foo.css"'
    'This is a Global Comment'

Structure read from the file (uncludes COMMENT and CDATA sections):
    PROCESSING_INSTRUCTION: 'xml-stylesheet type="text/css" href="foo.css"'
                   COMMENT: 'This is a Global Comment'
                       aaa: [1x1 struct]
                            PROCESSING_INSTRUCTION: 'ProcInst type="local processing instruction"'
                                           COMMENT: [1x2 cell] = 
                                                    local comment 1

                                                    local comment 2

                                           CONTENT: [1x2 cell] = 
                                                    bbb

                                                    ccc

                                            matrix: [1x1 struct]
                                                      CONTENT: [2x2 double]
                                                    ATTRIBUTE: [1x1 struct]
                                                               bad_DASH_name: 'fff'
                                              ee_e: 'ee_e'
                                         ff_DASH_f: 'ff-f'
                                           gg0xF6g: 'ggög'
                                     CDATA_SECTION: 'Here <ddd>xml</ddd> tags are treated as ...
    ... text'
                                         ATTRIBUTE: [1x1 struct]
                                                    xmlns_COLON_xsi: 'http://www.foo.org'
```

## "Pref.ReadAttr" flag in "xml\_read" (control handling of nodes with attributes)

Those option allow exclusion of attributes

```
Pref=[]; Pref.ReadAttr = false; % do not read attributes
tree = xml_read('test_file.xml', Pref);
gen_object_display(tree);
```

```
    PROCESSING_INSTRUCTION: 'ProcInst type="local processing instruction"'
                   COMMENT: [1x2 cell] = 
                            local comment 1

                            local comment 2

                   CONTENT: [1x2 cell] = 
                            bbb

                            ccc

                    matrix: [2x2 double]
                      ee_e: 'ee_e'
                 ff_DASH_f: 'ff-f'
                   gg0xF6g: 'ggög'
             CDATA_SECTION: 'Here <ddd>xml</ddd> tags are treated as ...
    ... text'
```

## "Pref.ReadSpec" flag in "xml\_read"

Those option allow exclusion of special nodes, like comments, processing instructions, CData sections, etc.

```
Pref=[]; Pref.ReadSpec = false; % do not read special node types
tree = xml_read('test_file.xml', Pref);
gen_object_display(tree);
```

```
      CONTENT: [1x2 cell] = 
               bbb

               ccc

       matrix: [1x1 struct]
                 CONTENT: [2x2 double]
               ATTRIBUTE: [1x1 struct]
                          bad_DASH_name: 'fff'
         ee_e: 'ee_e'
    ff_DASH_f: 'ff-f'
      gg0xF6g: 'ggög'
    ATTRIBUTE: [1x1 struct]
               xmlns_COLON_xsi: 'http://www.foo.org'
```

## "Pref.RootOnly" flag in "xml\_read"

As it was shown in previous examples RootOnly parameter can be used to capture global (top level) special nodes (like COMMENTs and PROCESSING\_INSTRUCTIONs) which are ignored by default

```
Pref=[]; Pref.RootOnly = false; % do not read special node types
tree = xml_read('test_file.xml', Pref);
gen_object_display(tree);
```

```
    PROCESSING_INSTRUCTION: 'xml-stylesheet type="text/css" href="foo.css"'
                   COMMENT: 'This is a Global Comment'
                       aaa: [1x1 struct]
                            PROCESSING_INSTRUCTION: 'ProcInst type="local processing instruction"'
                                           COMMENT: [1x2 cell] = 
                                                    local comment 1

                                                    local comment 2

                                           CONTENT: [1x2 cell] = 
                                                    bbb

                                                    ccc

                                            matrix: [1x1 struct]
                                                      CONTENT: [2x2 double]
                                                    ATTRIBUTE: [1x1 struct]
                                                               bad_DASH_name: 'fff'
                                              ee_e: 'ee_e'
                                         ff_DASH_f: 'ff-f'
                                           gg0xF6g: 'ggög'
                                     CDATA_SECTION: 'Here <ddd>xml</ddd> tags are treated as ...
    ... text'
                                         ATTRIBUTE: [1x1 struct]
                                                    xmlns_COLON_xsi: 'http://www.foo.org'
```

## "Pref.RootOnly" flag in "xml\_write"

Writing previously read tree with default "Pref.RootOnly = true" gives wrong output file

```
Pref=[]; Pref.RootOnly = true; % do not read special node types
xml_write('test.xml', tree, [], Pref);
fprintf('Test xml file:\n');
type('test.xml')
```

```
Test xml file:

<?xml version="1.0" encoding="utf-8"?>
<tree><?xml-stylesheet type="text/css" href="foo.css"?><!--This is a Global Comment-->
   <aaa xmlns:xsi="http://www.foo.org"><?ProcInst type="local processing instruction"?><!--local comment 1--><!--local comment 2-->
      <item>bbb</item>
      <item>ccc</item>
      <matrix bad-name="fff">[5000+i*2 Inf;NaN 3.14159265358979]</matrix>
      <ee_e>ee_e</ee_e>
      <ff-f>ff-f</ff-f>
      <ggÃ¶g>ggÃ¶g</ggÃ¶g>Here &lt;ddd&gt;xml&lt;/ddd&gt; tags are treated as ...
    ... text</aaa>
</tree>
```

Writing the same tree with "Pref.RootOnly = false" gives correct output

```
Pref=[]; Pref.RootOnly = false; % do not read special node types
xml_write('test.xml', tree, [], Pref);
fprintf('Test xml file:\n');
type('test.xml')
```

```
Test xml file:

<?xml version="1.0" encoding="utf-8"?><?xml-stylesheet type="text/css" href="foo.css"?><!--This is a Global Comment-->
<aaa xmlns:xsi="http://www.foo.org"><?ProcInst type="local processing instruction"?><!--local comment 1--><!--local comment 2-->
   <item>bbb</item>
   <item>ccc</item>
   <matrix bad-name="fff">[5000+i*2 Inf;NaN 3.14159265358979]</matrix>
   <ee_e>ee_e</ee_e>
   <ff-f>ff-f</ff-f>
   <ggÃ¶g>ggÃ¶g</ggÃ¶g>Here &lt;ddd&gt;xml&lt;/ddd&gt; tags are treated as ...
    ... text</aaa>
```

## "Pref.NumLevels" flag in "xml\_read"

This parameter allows user to skip parts of the tree in order to save time and memory. Usefull only in a rare case when a small portion of large XML file is needed.

Create test tile

```
MyTree = [];
MyTree.Level1 = 1;
MyTree.Level1_.Level2 = 2;
MyTree.Level1_.Level2_.Level3 = 3;
MyTree.Level1_.Level2_.Level3_.Level4 = 4;
xml_write('test.xml', MyTree);
fprintf('Test xml file:\n');
type('test.xml')
```

```
Test xml file:

<?xml version="1.0" encoding="utf-8"?>
<MyTree>
   <Level1>1</Level1>
   <Level1_>
      <Level2>2</Level2>
      <Level2_>
         <Level3>3</Level3>
         <Level3_>
            <Level4>4</Level4>
         </Level3_>
      </Level2_>
   </Level1_>
</MyTree>
```

**Use Default ("Pref.NumLevels = infinity") setting**

```
tree = xml_read('test.xml');
gen_object_display(tree);
```

```
     Level1: [1]
    Level1_: [1x1 struct]
              Level2: [2]
             Level2_: [1x1 struct]
                       Level3: [3]
                      Level3_: [1x1 struct]
                               Level4: [4]
```

**Limit the read to only 2 levels**

```
Pref=[]; Pref.NumLevels = 2;
tree = xml_read('test.xml', Pref);
gen_object_display(tree);
```

```
     Level1: [1]
    Level1_: [1x1 struct]
              Level2: [2]
             Level2_: [0x0 double]
```

## Create DOM object based on a Struct using "xml\_write"

**Create Struct tree**

```
MyTree=[];
MyTree.MyNumber = 13;
MyTree.MyString = 'Hello World';
```

**Convert Struct to DOM object using xml\_write**

```
DOM = xml_write([], MyTree);
xmlwrite('test.xml', DOM);   % Save DOM object using MATLAB function
type('test.xml')
```

```
<?xml version="1.0" encoding="utf-8"?>
<MyTree>
   <MyNumber>13</MyNumber>
   <MyString>Hello World</MyString>
</MyTree>
```

## Convert DOM object to Struct using "xml\_read"

```
DOM = xmlread('test.xml');       % Read DOM object using MATLAB function
[tree treeName] = xml_read(DOM); % Convert DOM object to Struct
disp([treeName{1} ' ='])
gen_object_display(tree)
```

```
MyTree =
    MyNumber: [13]
    MyString: 'Hello World'
```

## Write XML file based on a DOM using "xml\_write\_xerces"

```
xmlwrite_xerces('test.xml', DOM); % Save DOM object using Xerces library
type('test.xml')
```

```
<?xml version="1.0" encoding="UTF-8"?>
<MyTree>
    <MyNumber>13</MyNumber>
    <MyString>Hello World</MyString>
</MyTree>
```

## Write XML to string instead of a file

```
DOM = xml_write([], MyTree);
str = xmlwrite(DOM);
disp(str)
```

```
<?xml version="1.0" encoding="utf-8"?>
<MyTree>
   <MyNumber>13</MyNumber>
   <MyString>Hello World</MyString>
</MyTree>
```

## Write XML file with embedded binary data encoded as Base64 (using java version)

```
fid = fopen('football.jpg', 'rb');
raw1 = uint8(fread(fid, 'uint8'));                % read image file as a raw binary
fclose(fid);

MyTree=[];
MyTree.Size = 13;
MyTree.MyString = 'Hello World'; % simple case
MyTree.MyImage.ATTRIBUTE.EncodingMIMEType = 'base64';
MyTree.MyImage.CONTENT = base64encode(raw1,'java');% perform base64 encoding of the binary data
xml_write('test.xml', MyTree);             % write xml file
```

## Read XML file with embedded binary data encoded as Base64 (using java version)

```
tree = xml_read('test.xml', Pref);         % read xml file
raw  = base64decode(tree.MyImage.CONTENT, '', 'java');   % convert xml image to raw binary
fid = fopen('MyFootball.jpg', 'wb');
fwrite(fid, raw, 'uint8');                 % dumb the raw binary to the hard disk
fclose(fid);
I = imread('MyFootball.jpg');              % read it as an image
imshow(I);
```

## Write XML file with embedded binary data encoded as Base64 (simpler version using only matlab code

Notice that process of writing to xml stripped all end-of-lie characters from base64 code.

```
isChunked = true; % break into chunks 76 characters long
url_safe  = true; % 'base64url' encoding
code = base64encode('license.txt', 'matlab', isChunked, url_safe);
disp(code)
MyTree=[];
MyTree.Size = 13;
MyTree.MyString = 'Hello World';
MyTree.MyImage.ATTRIBUTE.EncodingMIMEType = 'base64';
MyTree.MyImage.CONTENT = code;   % perform base64 encoding of the binary data
xml_write('test.xml', MyTree);   % write xml file
type('test.xml')
```

```
Q29weXJpZ2h0IChjKSAyMDA3LCBKYXJvc2xhdyBUdXN6eW5za2kKQWxsIHJpZ2h0cyByZXNlcnZl
ZC4KClJlZGlzdHJpYnV0aW9uIGFuZCB1c2UgaW4gc291cmNlIGFuZCBiaW5hcnkgZm9ybXMsIHdp
dGggb3Igd2l0aG91dCAKbW9kaWZpY2F0aW9uLCBhcmUgcGVybWl0dGVkIHByb3ZpZGVkIHRoYXQg
dGhlIGZvbGxvd2luZyBjb25kaXRpb25zIGFyZSAKbWV0OgoKICAgICogUmVkaXN0cmlidXRpb25z
IG9mIHNvdXJjZSBjb2RlIG11c3QgcmV0YWluIHRoZSBhYm92ZSBjb3B5cmlnaHQgCiAgICAgIG5v
dGljZSwgdGhpcyBsaXN0IG9mIGNvbmRpdGlvbnMgYW5kIHRoZSBmb2xsb3dpbmcgZGlzY2xhaW1l
ci4KICAgICogUmVkaXN0cmlidXRpb25zIGluIGJpbmFyeSBmb3JtIG11c3QgcmVwcm9kdWNlIHRo
ZSBhYm92ZSBjb3B5cmlnaHQgCiAgICAgIG5vdGljZSwgdGhpcyBsaXN0IG9mIGNvbmRpdGlvbnMg
YW5kIHRoZSBmb2xsb3dpbmcgZGlzY2xhaW1lciBpbiAKICAgICAgdGhlIGRvY3VtZW50YXRpb24g
YW5kL29yIG90aGVyIG1hdGVyaWFscyBwcm92aWRlZCB3aXRoIHRoZSBkaXN0cmlidXRpb24KICAg
ICAgClRISVMgU09GVFdBUkUgSVMgUFJPVklERUQgQlkgVEhFIENPUFlSSUdIVCBIT0xERVJTIEFO
RCBDT05UUklCVVRPUlMgIkFTIElTIiAKQU5EIEFOWSBFWFBSRVNTIE9SIElNUExJRUQgV0FSUkFO
VElFUywgSU5DTFVESU5HLCBCVVQgTk9UIExJTUlURUQgVE8sIFRIRSAKSU1QTElFRCBXQVJSQU5U
SUVTIE9GIE1FUkNIQU5UQUJJTElUWSBBTkQgRklUTkVTUyBGT1IgQSBQQVJUSUNVTEFSIFBVUlBP
U0UgCkFSRSBESVNDTEFJTUVELiBJTiBOTyBFVkVOVCBTSEFMTCBUSEUgQ09QWVJJR0hUIE9XTkVS
IE9SIENPTlRSSUJVVE9SUyBCRSAKTElBQkxFIEZPUiBBTlkgRElSRUNULCBJTkRJUkVDVCwgSU5D
SURFTlRBTCwgU1BFQ0lBTCwgRVhFTVBMQVJZLCBPUiAKQ09OU0VRVUVOVElBTCBEQU1BR0VTIChJ
TkNMVURJTkcsIEJVVCBOT1QgTElNSVRFRCBUTywgUFJPQ1VSRU1FTlQgT0YgClNVQlNUSVRVVEUg
R09PRFMgT1IgU0VSVklDRVM7IExPU1MgT0YgVVNFLCBEQVRBLCBPUiBQUk9GSVRTOyBPUiBCVVNJ
TkVTUyAKSU5URVJSVVBUSU9OKSBIT1dFVkVSIENBVVNFRCBBTkQgT04gQU5ZIFRIRU9SWSBPRiBM
SUFCSUxJVFksIFdIRVRIRVIgSU4gCkNPTlRSQUNULCBTVFJJQ1QgTElBQklMSVRZLCBPUiBUT1JU
IChJTkNMVURJTkcgTkVHTElHRU5DRSBPUiBPVEhFUldJU0UpIApBUklTSU5HIElOIEFOWSBXQVkg
T1VUIE9GIFRIRSBVU0UgT0YgVEhJUyBTT0ZUV0FSRSwgRVZFTiBJRiBBRFZJU0VEIE9GIFRIRSAK
UE9TU0lCSUxJVFkgT0YgU1VDSCBEQU1BR0UuCg==


<?xml version="1.0" encoding="utf-8"?>
<MyTree>
   <Size>13</Size>
   <MyString>Hello World</MyString>
   <MyImage EncodingMIMEType="base64">Q29weXJpZ2h0IChjKSAyMDA3LCBKYXJvc2xhdyBUdXN6eW5za2kKQWxsIHJpZ2h0cyByZXNlcnZl ZC4KClJlZGlzdHJpYnV0aW9uIGFuZCB1c2UgaW4gc291cmNlIGFuZCBiaW5hcnkgZm9ybXMsIHdp dGggb3Igd2l0aG91dCAKbW9kaWZpY2F0aW9uLCBhcmUgcGVybWl0dGVkIHByb3ZpZGVkIHRoYXQg dGhlIGZvbGxvd2luZyBjb25kaXRpb25zIGFyZSAKbWV0OgoKICAgICogUmVkaXN0cmlidXRpb25z IG9mIHNvdXJjZSBjb2RlIG11c3QgcmV0YWluIHRoZSBhYm92ZSBjb3B5cmlnaHQgCiAgICAgIG5v dGljZSwgdGhpcyBsaXN0IG9mIGNvbmRpdGlvbnMgYW5kIHRoZSBmb2xsb3dpbmcgZGlzY2xhaW1l ci4KICAgICogUmVkaXN0cmlidXRpb25zIGluIGJpbmFyeSBmb3JtIG11c3QgcmVwcm9kdWNlIHRo ZSBhYm92ZSBjb3B5cmlnaHQgCiAgICAgIG5vdGljZSwgdGhpcyBsaXN0IG9mIGNvbmRpdGlvbnMg YW5kIHRoZSBmb2xsb3dpbmcgZGlzY2xhaW1lciBpbiAKICAgICAgdGhlIGRvY3VtZW50YXRpb24g YW5kL29yIG90aGVyIG1hdGVyaWFscyBwcm92aWRlZCB3aXRoIHRoZSBkaXN0cmlidXRpb24KICAg ICAgClRISVMgU09GVFdBUkUgSVMgUFJPVklERUQgQlkgVEhFIENPUFlSSUdIVCBIT0xERVJTIEFO RCBDT05UUklCVVRPUlMgIkFTIElTIiAKQU5EIEFOWSBFWFBSRVNTIE9SIElNUExJRUQgV0FSUkFO VElFUywgSU5DTFVESU5HLCBCVVQgTk9UIExJTUlURUQgVE8sIFRIRSAKSU1QTElFRCBXQVJSQU5U SUVTIE9GIE1FUkNIQU5UQUJJTElUWSBBTkQgRklUTkVTUyBGT1IgQSBQQVJUSUNVTEFSIFBVUlBP U0UgCkFSRSBESVNDTEFJTUVELiBJTiBOTyBFVkVOVCBTSEFMTCBUSEUgQ09QWVJJR0hUIE9XTkVS IE9SIENPTlRSSUJVVE9SUyBCRSAKTElBQkxFIEZPUiBBTlkgRElSRUNULCBJTkRJUkVDVCwgSU5D SURFTlRBTCwgU1BFQ0lBTCwgRVhFTVBMQVJZLCBPUiAKQ09OU0VRVUVOVElBTCBEQU1BR0VTIChJ TkNMVURJTkcsIEJVVCBOT1QgTElNSVRFRCBUTywgUFJPQ1VSRU1FTlQgT0YgClNVQlNUSVRVVEUg R09PRFMgT1IgU0VSVklDRVM7IExPU1MgT0YgVVNFLCBEQVRBLCBPUiBQUk9GSVRTOyBPUiBCVVNJ TkVTUyAKSU5URVJSVVBUSU9OKSBIT1dFVkVSIENBVVNFRCBBTkQgT04gQU5ZIFRIRU9SWSBPRiBM SUFCSUxJVFksIFdIRVRIRVIgSU4gCkNPTlRSQUNULCBTVFJJQ1QgTElBQklMSVRZLCBPUiBUT1JU IChJTkNMVURJTkcgTkVHTElHRU5DRSBPUiBPVEhFUldJU0UpIApBUklTSU5HIElOIEFOWSBXQVkg T1VUIE9GIFRIRSBVU0UgT0YgVEhJUyBTT0ZUV0FSRSwgRVZFTiBJRiBBRFZJU0VEIE9GIFRIRSAK UE9TU0lCSUxJVFkgT0YgU1VDSCBEQU1BR0UuCg==</MyImage>
</MyTree>
```

## Read XML file with embedded binary data encoded as Base64 (simpler version using only matlab code

```
tree = xml_read('test.xml', Pref);         % read xml file
base64decode(tree.MyImage.CONTENT, 'license2.txt', 'matlab'); % save xml image as raw binary
type('license2.txt')
```

```
Copyright (c) 2007, Jaroslaw Tuszynski
All rights reserved.

Redistribution and use in source and binary forms, with or without 
modification, are permitted provided that the following conditions are 
met:

    * Redistributions of source code must retain the above copyright 
      notice, this list of conditions and the following disclaimer.
    * Redistributions in binary form must reproduce the above copyright 
      notice, this list of conditions and the following disclaimer in 
      the documentation and/or other materials provided with the distribution
      
THIS SOFTWARE IS PROVIDED BY THE COPYRIGHT HOLDERS AND CONTRIBUTORS "AS IS" 
AND ANY EXPRESS OR IMPLIED WARRANTIES, INCLUDING, BUT NOT LIMITED TO, THE 
IMPLIED WARRANTIES OF MERCHANTABILITY AND FITNESS FOR A PARTICULAR PURPOSE 
ARE DISCLAIMED. IN NO EVENT SHALL THE COPYRIGHT OWNER OR CONTRIBUTORS BE 
LIABLE FOR ANY DIRECT, INDIRECT, INCIDENTAL, SPECIAL, EXEMPLARY, OR 
CONSEQUENTIAL DAMAGES (INCLUDING, BUT NOT LIMITED TO, PROCUREMENT OF 
SUBSTITUTE GOODS OR SERVICES; LOSS OF USE, DATA, OR PROFITS; OR BUSINESS 
INTERRUPTION) HOWEVER CAUSED AND ON ANY THEORY OF LIABILITY, WHETHER IN 
CONTRACT, STRICT LIABILITY, OR TORT (INCLUDING NEGLIGENCE OR OTHERWISE) 
ARISING IN ANY WAY OUT OF THE USE OF THIS SOFTWARE, EVEN IF ADVISED OF THE 
POSSIBILITY OF SUCH DAMAGE.
```

Published with MATLAB® 7.10
